# True Molecular Scale Visualization of Variable Clustering Properties of Ryanodine Receptors

**DOI:** 10.1016/j.celrep.2017.12.045

**Published:** 2018-01-09

**Authors:** Izzy Jayasinghe, Alexander H. Clowsley, Ruisheng Lin, Tobias Lutz, Carl Harrison, Ellen Green, David Baddeley, Lorenzo Di Michele, Christian Soeller

**Affiliations:** 1 Faculty of Biological Sciences, University of Leeds, Leeds, UK; 2 Living Systems Institute, University of Exeter, Exeter, UK; 3 Auckland Bioengineering Institute, University of Auckland, Auckland, New Zealand; 4 Cavendish Laboratory, University of Cambridge, Cambridge, UK

**Keywords:** nanodomains, DNA-PAINT, single-molecule localization microscopy, ryanodine receptor, super-resolution imaging, junctophilin, heart

## Abstract

Signaling nanodomains rely on spatial organization of proteins to allow controlled intracellular signaling. Examples include calcium release sites of cardiomyocytes where ryanodine receptors (RyRs) are clustered with their molecular partners. Localization microscopy has been crucial to visualizing these nanodomains but has been limited by brightness of markers, restricting the resolution and quantification of individual proteins clustered within. Harnessing the remarkable localization precision of DNA-PAINT (<10 nm), we visualized punctate labeling within these nanodomains, confirmed as single RyRs. RyR positions within sub-plasmalemmal nanodomains revealed how they are organized randomly into irregular clustering patterns leaving significant gaps occupied by accessory or regulatory proteins. RyR-inhibiting protein junctophilin-2 appeared highly concentrated adjacent to RyR channels. Analyzing these molecular maps showed significant variations in the co-clustering stoichiometry between junctophilin-2 and RyR, even between nearby nanodomains. This constitutes an additional level of complexity in RyR arrangement and regulation of calcium signaling, intrinsically built into the nanodomains.

## Highlights

• With enhanced resolution, individual RyRs were observed within dense clusters
• RyRs appear to assemble randomly and unconstrained
• The ratio of RyR co-clustering with JPH2 varies considerably between nanodomains
• DNA-based super-resolution enables fully quantitative imaging at ∼10 nm resolution

## Graphical Abstract

**Figure:**
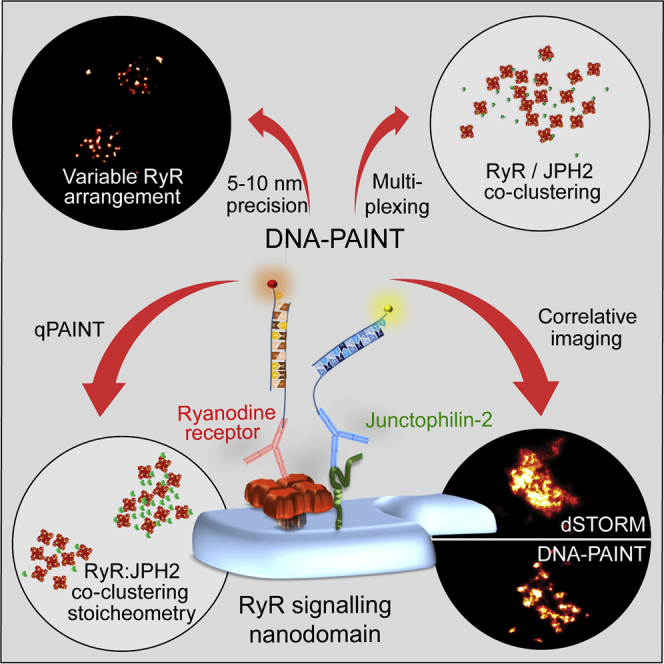

## Teaser

Jayasinghe et al. resolve the distribution of single ryanodine receptors (RyRs) within intracellular signaling domains in cardiac myocytes with DNA-PAINT, a super-resolution microscopy approach. Individual RyRs are resolved within irregular cluster arrays. Quantitative imaging reveals significant variation in the co-clustering stoichiometry between RyRs and the regulatory protein junctophilin-2.

## Introduction

The advent of single-molecule switching and localization-based PALM, STORM, and related super-resolution microscopies (Betzig et al., 2006, Hess et al., 2006, Rust et al., 2006) has greatly advanced insight in cell biology over the last decade. Significant breakthroughs in visualizing nanostructures within cells include optically resolved nuclear pore complexes (Szymborska et al., 2013), microtubules (Mikhaylova et al., 2015), actin-spectrin scaffolds for membranes (Xu et al., 2013), membrane compartments (Shim et al., 2012), and protein ensembles in signaling nanodomains (Gambin et al., 2013). This includes the visualization of the clusters of the giant (∼2 MDa) ryanodine receptor-2 (RyR) Ca^2+^ release channels in cardiomyocytes to characterize the calcium signaling nanodomains, which are the structural units of calcium signaling in cardiac myocytes (Baddeley et al., 2009) using dSTORM (Heilemann et al., 2008).

The cardiac ryanodine receptor, RyR2, is strongly expressed in the heart and brain and provides the molecular basis of a mechanism known as calcium-induced calcium release (CICR), in which RyRs are transiently opened via calcium from adjacent calcium channels (Fabiato, 1985, Franzini-Armstrong and Protasi, 1997). Due to their vital role in physiology and pathology RyRs present a key target for molecular investigations. At the supra-molecular level the clustering of RyRs is of major interest, both because it can dramatically modulate the excitability of RyRs (Walker et al., 2014) and due to their general role in calcium signaling in muscle (Allen et al., 2008, Cannell and Kong, 2012), neurons (Manita and Ross, 2009), and secretory cells like pancreatic beta cells.

RyR clusters are a prototypical system for which the biophysical cluster properties, e.g., cluster excitability, can be directly tied to the spatial cluster arrangement of the RyRs as recently shown (Walker et al., 2014). The regulation of RyR channels is under the acute control of both cytoplasmic and internal store [Ca^2+^] but is also a product of the local configuration of other molecular components. These include accessory proteins such as junctophilin-2 (JPH2), which are anchored in the intracellular store membrane. Local JPH2 can directly modulate the Ca^2+^ released by RyRs as well as adjusting the physical size of RyR clusters (Munro et al., 2016).

In previous work, application of dSTORM (or stimulated emission depletion [STED]) to study the clustering properties of RyRs within cardiomyocytes achieved effective lateral spatial resolution of 40–60 nm, with quantitative analysis based on the assumption that RyRs are arranged on a regular 30 × 30-nm quasi-crystalline lattice (Baddeley et al., 2009). Electron tomography data have recently challenged this assumption and suggested that the arrangement may be more variable (Asghari et al., 2014); due to the complexities of electron tomography, this has not been replicated in other laboratories to date. To resolve such open questions there is a growing need for quantitative super-resolution methods, which can consistently and robustly achieve higher resolution in complex cells.

A recent microscopy approach known as DNA-PAINT utilizes the specificity and predictability of DNA hybridization to localize molecular targets tagged with synthetic single-stranded DNA oligonucleotides (Jungmann et al., 2014). When compared with more conventional localization microscopy approaches using switchable labels DNA-PAINT has two important advantages. It allows the use of extremely bright and stable dyes along with buffer conditions that optimize photon yield (rather than switching performance) leading to significantly improved localization precision. In addition, it is compatible with high accuracy target quantification given suitable calibration (Jungmann et al., 2016).

In this study, we achieve true molecular resolution of RyRs within tightly organized junctional nanodomains, enabled by DNA-PAINT, to understand the *in situ* molecular interactions of RyR and the related junctional molecule JPH2. We developed a nanoscale Monte Carlo simulation of protein cluster assembly, to test hypotheses on mechanisms of protein clustering, and uncertainties in protein labeling. The observed RyR patterns within clusters are consistent with a random and unconstrained cluster assembly process, unlike the crystalline lattices seen in *in vitro* assays and in skeletal muscle cells. Quantitative co-localization analysis of RyRs and JPH2 revealed a fraction of JPH2 within 50 nm of RyRs, consisted with RyR-JPH2 complex formation. Using a qPAINT target counting algorithm, we made the striking observation that the ratio of RyRs co-clustering with its inhibitory partner protein JPH2 can vary manifold. Overall, the data reveal that RyR nanodomains exhibit localized structural variations compatible with extremely different excitability and calcium release properties.

## Results

### Improved Visualization of RyRs within Nanodomains

**Figure 1:**
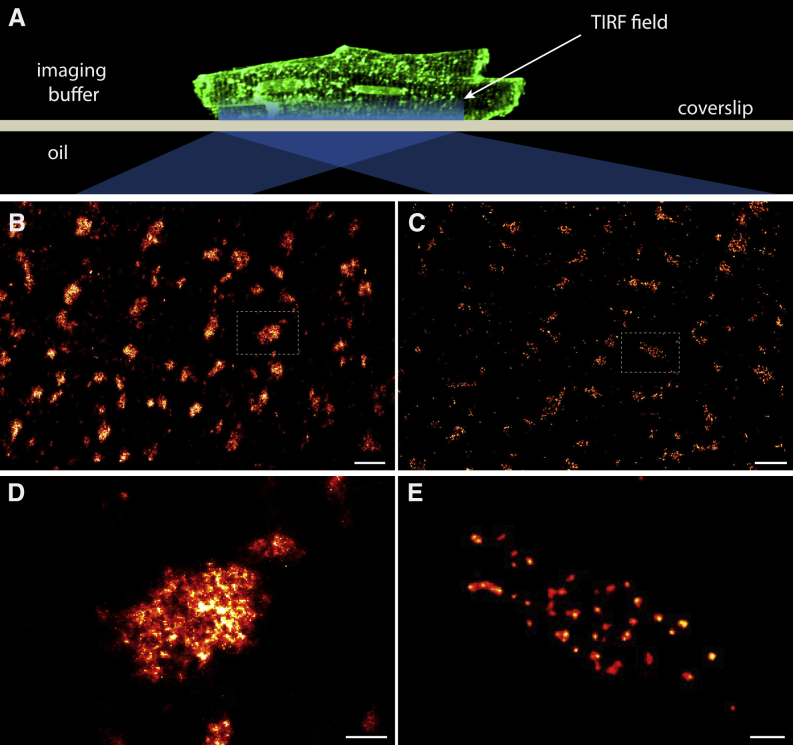
Visualization of RyRs in Peripheral Couplons of Ventricular Myocytes (A) TIRF illumination of peripheral RyR labels adjacent to the coverslip. (B and C) Both dSTORM (B) and DNA-PAINT (C) typically show RyR in clustered nanodomains (which are loosely in a transverse striated pattern) facing the surface plasmalemma (parallel to image plane). (D and E) Magnified views of clusters from these images (boxed regions in B and C) show the unresolved cluster sub-structure in dSTORM data (D), which contrasts with resolved punctate RyR patterns mapped with DNA-PAINT (E). Scale bars in (B) and (C), 1 μm, and in (D) and (E), 100 nm.

To visualize the immuno-labeling of RyRs in peripheral clusters (or “couplons”) of rat ventricular myocytes, cells adhered to coverslips were studied in total internal reflection fluorescence (TIRF) mode (Figures 1A and S1). dSTORM images of surface membrane areas attached to the coverslip revealed clusters of RyR labeling loosely organized in a transversely striated pattern (Figure 1B), as seen before (Baddeley et al., 2009). DNA-PAINT imaging of RyR in similar cells was performed in very similar imaging settings allowing single-marker detection as the “imager” oligonucleotides bind to their complementary “docking” strands within the TIRF field (Figures S1A and S1B). Grayscale rendering of the imager localizations (Figure 1C) produced a labeling pattern, which broadly reflects the periodic arrangement of sarcomeres with most RyRs close to the z-lines (Baddeley et al., 2009), similar to the distribution seen with dSTORM.

At closer inspection, however, the DNA-PAINT images reveal very distinctive puncta within the cluster area unlike in the dSTORM images (Figures 1D and 1E). Even in the primary event localization position maps (Figures S1C and S1D), this pattern was clearly discernible. Examination of the reason for this improved resolution of RyR markers revealed a higher photon yield in DNA-PAINT events (Figures S1E), which contributed to an ∼6-fold improvement in localization error (Figure S1F).

### Reproducibility of RyR Localization with DNA-PAINT

We hypothesized that these puncta reveal individual RyRs within the essentially flat 2D peripheral clusters (Franzini-Armstrong et al., 1999), which dSTORM data could not resolve. We conducted a series of experiments to reconcile this morphology with the previously observed dSTORM images of the same structures as well as experiments to confirm that the morphology observed in the DNA-PAINT data is robust.

Similar results were obtained with directly labeled primary antibodies against RyRs as with the secondary labeling system (Figure S3), due to the relatively large size of RyRs (∼30 nm); we therefore adopted the secondary system for most experiments.

**Figure 2:**
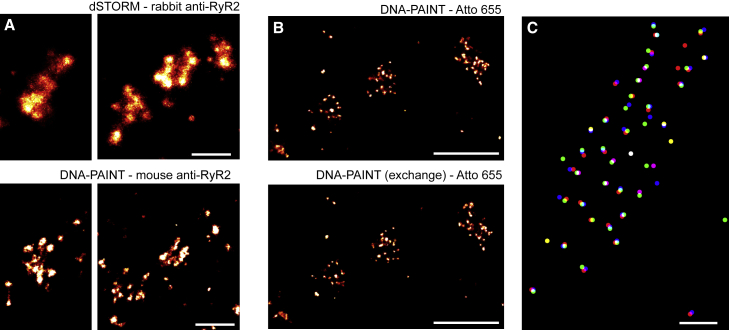
Reproducibility of RyR Cluster Nanostructure with DNA-PAINT Imaging (A) dSTORM images of RyR clusters (upper) in peripheral nanodomains showed clear visual agreement with correlative DNA-PAINT images of the same clusters (lower). (B) Sequential exchange-PAINT images acquired of RyR clusters illustrate the high reproducibility of both cluster shapes and punctate morphology. (C) Overlay of the centroids of each RyR punctum in three independently acquired DNA-PAINT images (red, green, and blue) of the same cell confirms this. Scale bar in (A), 250 nm, in (B), 500 nm, and in (C), 250 nm.

We also assessed the consistency between DNA-PAINT reported RyR clusters and dSTORM cluster data. A correlative imaging experiment where DNA-PAINT (using ATTO 655 imagers) and dSTORM (using Alexa Fluor 647 secondary antibodies) were performed using a mouse monoclonal anti-RyR2 and a rabbit polyclonal anti-RyR2 (previously shown with dSTORM to report >70% spatial agreement [Hou et al., 2015]), respectively. The correlative dSTORM and DNA-PAINT images (Figure 2A) revealed strong spatial agreement of the cluster positions and the shapes. They also confirmed that the punctate RyR labeling in DNA-PAINT images were essentially confined to the cluster area resolved with dSTORM.

DNA-PAINT has “photo-bleaching free” properties due to unlimited replenishment of imagers. We exploited this for sequentially acquiring correlative DNA-PAINT images of the same RyR clusters. This was achieved by washing imager strands matching the RyR secondary antibodies repeatedly in and out again as done in exchange-PAINT (Jungmann et al., 2014) (Figure 2B). These experiments established that the locations and the morphology of the puncta were robustly reproduced with a median error (displacement of a given punctum in a pairwise comparison of images) of 0.94 nm (47 clusters; 5 cells). 94.2% ± 0.9% (mean ± SEM, 5 cells) of puncta locations were reproduced within 10 nm (80.9% within a stringency of 5 nm) across three sets of exchange-PAINT images sets of DNA-PAINT images in the correlated areas (47 clusters; 5 cells). An example of this reproducibility is illustrated in Figure 2C where localized and detected puncta positions from three independent exchange-PAINT repetitions (red, blue, and green dots) confirm strong alignment and reproducibility. These results show that the puncta are a robust feature of the DNA-PAINT RyR data.

Given the localization precision (5–10 nm), individual puncta represent locations of single RyRs as it is unlikely that markers bound to the same RyR can be resolved into distinguishable puncta. As a consistency check, we performed a simulation that confirmed that a regular grid of RyRs spaced as close as 30 nm can be resolved and detected at event densities similar to those shown in our data (e.g., Figure S2A). This is also broadly consistent with imaging of a test sample made from DNA origami on our system that clearly resolved 40-nm distant puncta using localization data with a similar precision as in our RyR data (Figures S3C and S3D).

The above tests hence confirmed that marker positions are robustly reproduced over several DNA-PAINT imaging repetitions and puncta occurred at a spacing compatible with detection based on the high localization precision data.

### RyR Clustering and Quantitative Analysis by *In Situ* Calibrated qPAINT

**Figure 3:**
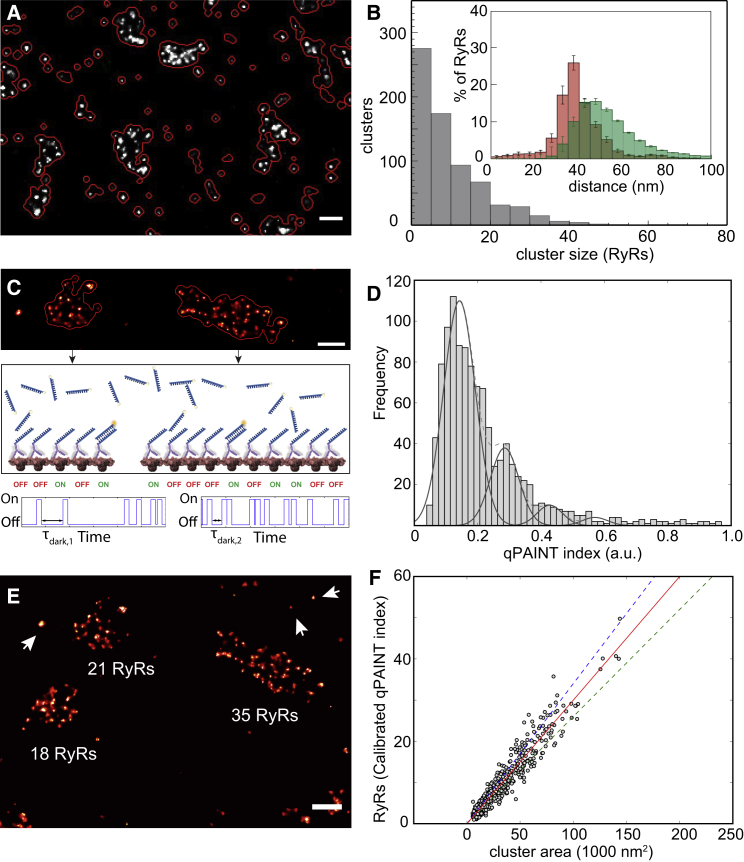
Quantitative Analysis of RyR Cluster Properties (A) Clusters were segmented using an algorithm that contours the image based on local event density (red lines). (B) A frequency histogram of RyR puncta counted within segmented peripheral RyR clusters typically consisted of fewer than 40 RyRs (mean ± SD = 8.81 ± 3.56 RyR/cluster; n = 17 cells, 10 animals); the distribution of nearest neighbor puncta centroid distances within clusters with 5 or more RyRs (see inset; red) showed a mean of 40.1 ± 0.9 nm (mean ± SEM; n = 1,802 clusters, 8 animals). The average of the 4 nearest neighbor distances (green) was a right-shifted distribution (58.9 ± 0.9 nm). Error bars (inset): SEM for n = 17 cells. (C) qPAINT estimates the number of docking sites by analyzing the temporal sequence of single-molecule event detections recorded at a given cluster. Two clusters are shown (upper panel), one larger than the other. The rate at which imagers bind to RyRs in a cluster is proportional to the number of RyRs in the cluster (middle and lower panels). (D) The “qPAINT indices” of small clusters containing ∼1 punctum provided a calibration to “count RyRs”; its histogram exhibits equidistant peaks characteristic of quantal increments in imager strand binding sites with a primary maximum of 0.14 qPAINT index units, equivalent to a single RyR. (E) Examples of the RyR counts estimated from calibrating the qPAINT index values for three nearby large clusters show the close correspondence with counted puncta numbers. (F) Scatterplot of cluster area versus qPAINT estimated RyR counts. A linear regression (solid line) of the scattergram provides an estimate of the two-dimensional area of packing as 1 RyR per 3,300 nm^2^ for this dataset from one cell (391 clusters). Dashed lines indicate 15% variation of slope. Scale bars in (A) and (E), 200 nm.

The reproducibility and the static positions of the punctate event densities observed in DNA-PAINT data are strong evidence that these reflect the positions of RyRs within the underlying cluster. The resolved RyR positions were useful in investigating clustering patterns and spatial densities of RyR arrangement, which critically determine their cross signaling via calcium (Walker et al., 2014). RyR numbers detected as in Figures S1D within 2,062 clusters (17 cells) segmented clusters (red outlines in Figure 3A), revealing a frequency histogram of cluster size with an approximately exponential distribution (Figure 3B). This distribution, in shape, was similar to that constructed previously with dSTORM data (Baddeley et al., 2009); however, with a smaller average size of 8.8 ± 0.86 (n = 17 cells) RyRs per cluster. To analyze clustering pattern, we measured the nearest neighbor center-to-center distance (NND) for each RyR punctum in clusters with ≥2 RyRs. From 21 cells analyzed, a near-Gaussian distribution with a mean of 40.1 ± 0.9 nm (Figure 3B inset; red) was observed. In a nanodomain with variable RyR spacing, typified by the DNA-PAINT images, the average of the distances to each of the surrounding neighbors would describe the typical calcium diffusion distances relevant to RyR-RyR communication. The average 4-neighbor distance (4ND) for these data (for clusters with ≥5 RyRs) had a mean of 58.9 ± 0.9 nm and a mode at ∼47 nm (Figure 3B inset; green).

We subjected the same RyR data to an algorithm called qPAINT, which quantifies protein numbers from DNA-PAINT data (Jungmann et al., 2014), as a second, largely independent analysis from the puncta counting approach. This method utilizes the average “off” times absent of an imager binding event within a cluster or nanodomain area (schematically shown in Figure 3C), under fixed imaging conditions. This off-time is proportional to the number of docking sites, i.e., the number of markers (see Figure S4 and Supplemental Experimental Procedures). We conducted the qPAINT analysis on a per-cluster basis (e.g., Figure 3A) to plot cumulative histograms of the dark time durations and obtain the average dark time between binding events unique to each cluster (e.g., Figure S4D). Due to the first-order binding kinetics between imager strands and docking strands, the inverse of the measured dark time, which we term the “qPAINT index,” provides a measure directly proportional to the number of docking strands in the cluster region (for details, see Experimental Procedures). Figure 3D shows a histogram of the qPAINT index of small clusters that were selected based on their measured geometrical area and visually contain ∼1 punctum when rendered (Figures S4E and S4F). The qPAINT index histogram of these small clusters exhibits a number of peaks that are a hallmark of “quantal” behavior representing one, two, three, etc., units characteristic of single RyRs. The mean qPAINT index for single RyR obtained in this way, e.g., 0.14 in Figure 3D, was used to calibrate the cluster qPAINT indices in the corresponding image and turn them into absolute RyR number estimates. Figure 3E illustrates RyR number estimates for 3 larger clusters. The estimated RyR numbers closely agree with the counted number of puncta in these clusters as shown in Figure S4G, further supporting our hypothesis that individual puncta correspond to individual RyRs. When the estimated RyR numbers of each cluster are plotted against the geometrical area of clusters (Figure 3F), a linear relationship was obtained that was used to estimate the apparent RyR density within clusters. The slope of this relationship was 0.32 ± 0.05 RyR / 1,000 nm^2^, (mean ± SEM, 1,507 clusters, 6 cells from 3 animals) equivalent to a mean linear distance between RyRs of ∼56 nm, similar to the distance obtained above as the 4ND (∼59 nm, see above).

This quantitative analysis concludes that in the high-quality DNA-PAINT data we can see markers identifying the location of individual RyRs. It also confirms that RyRs occur at a density within clusters that is lower than that expected in a close packed arrangement of RyRs with a limiting density of ∼1 RyR / 1,000 nm^2^, as seen *in vitro* (Yin and Lai, 2000).

### Analysis of the Organization of RyRs within Clusters

**Figure 4:**
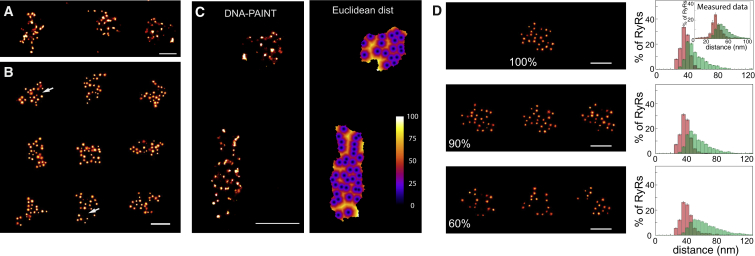
Morphology of RyR Organization within Clusters (A) DNA-PAINT example of three adjacent peripheral RyR clusters with their typical irregular cluster shapes and puncta arrangement. (B) Simulated super-resolution images with similar morphology were reproduced in an *in silico* Monte Carlo simulation of directionally unconstrained RyR cluster self-assembly. Note the gaps (arrows) within the arrays that naturally appeared within the cluster due to the random self-assembly process. (C) Experimental data (left) were further analyzed for the Euclidean distances (color maps in nm) between the centroids of RyR labeling densities. Regions colored in yellow-white were sub-nanodomain boundaries within the RyR cluster where the gaps exceeded 100 nm. (D) Simulation of effect of binding analysis by reducing the detection fraction from top to bottom panel 100%, 90%, and 60% (examples of iterations on left) revealed minor changes to the observed nearest-neighbor (red) and average-4-neighbor distance (green) histograms. Experimentally measured distance distributions are shown in inset. Error bars: SEM for n = clusters; (inset) SEM for n = 17 cells. Scale bars in (A) and (B), 200 nm, and (C), 400 nm, (D), 150 nm.

The clustering pattern of cardiac RyRs within their nanodomains is thought to be an important aspect determining the “excitability” of the nanodomain as a whole, and as the diffusion of Ca^2+^ between RyRs, which steeply depends on distance from open RyRs. The puncta positions analyzed above suggest that the spacings between RyRs are not uniform. This pattern is visually observable in DNA-PAINT data (Figure 3A). We sought to simulate clustering patterns that best captured these patterns. Experimental clusters could be closely mimicked by assembling simulated clusters according to a rule that constructed clusters by placing new RyRs in a random direction and with a distance that varied slightly around a mean distance of 40 nm according to a Gaussian distribution with a sigma of 7.4 nm (Figure 4B), a distribution that closely matches the observed distance distribution in mean and width (see also Figure 3B). This self-assembly process led to the appearance of some larger “gaps” in the clusters similar to those observed in our data as highlighted in distance maps between RyRs in experimentally observed clusters as shown in Figure 4C.

This simulation allowed us to investigate additional considerations in analyzing RyR patterns with a method such as DNA-PAINT. For example, the binding of antibodies to RyRs may be incomplete, which could affect the interpretation of the data. We sought to put bounds on these effects to inform data interpretation. Comparison of simulations based on clusters generated with the random direction cluster model and assuming detection levels of 100%, 90%, and 60% are shown in Figure 4D (extended in Figure S2B). Increasingly lower detection fractions shift the average of local distances to longer values and especially give rise to a marked tail in the 4ND distribution. By contrast, the NND stays approximately constant. Comparison suggests that our observations are consistent with a model where we detect >80% of all RyRs as otherwise gaps and 4NDs would markedly increase. The simulated clusters, the shapes, and modes of their histograms also confirmed that the 40-nm RyR spacing we observed in the experimental data (Figure 3B inset) could not be the result of lower detection efficiency (50%) of more closely spaced RyRs (30 nm). See Figure S2C.

### Investigation of RyR-JPH Co-clusters with Exchange-PAINT

**Figure 5:**
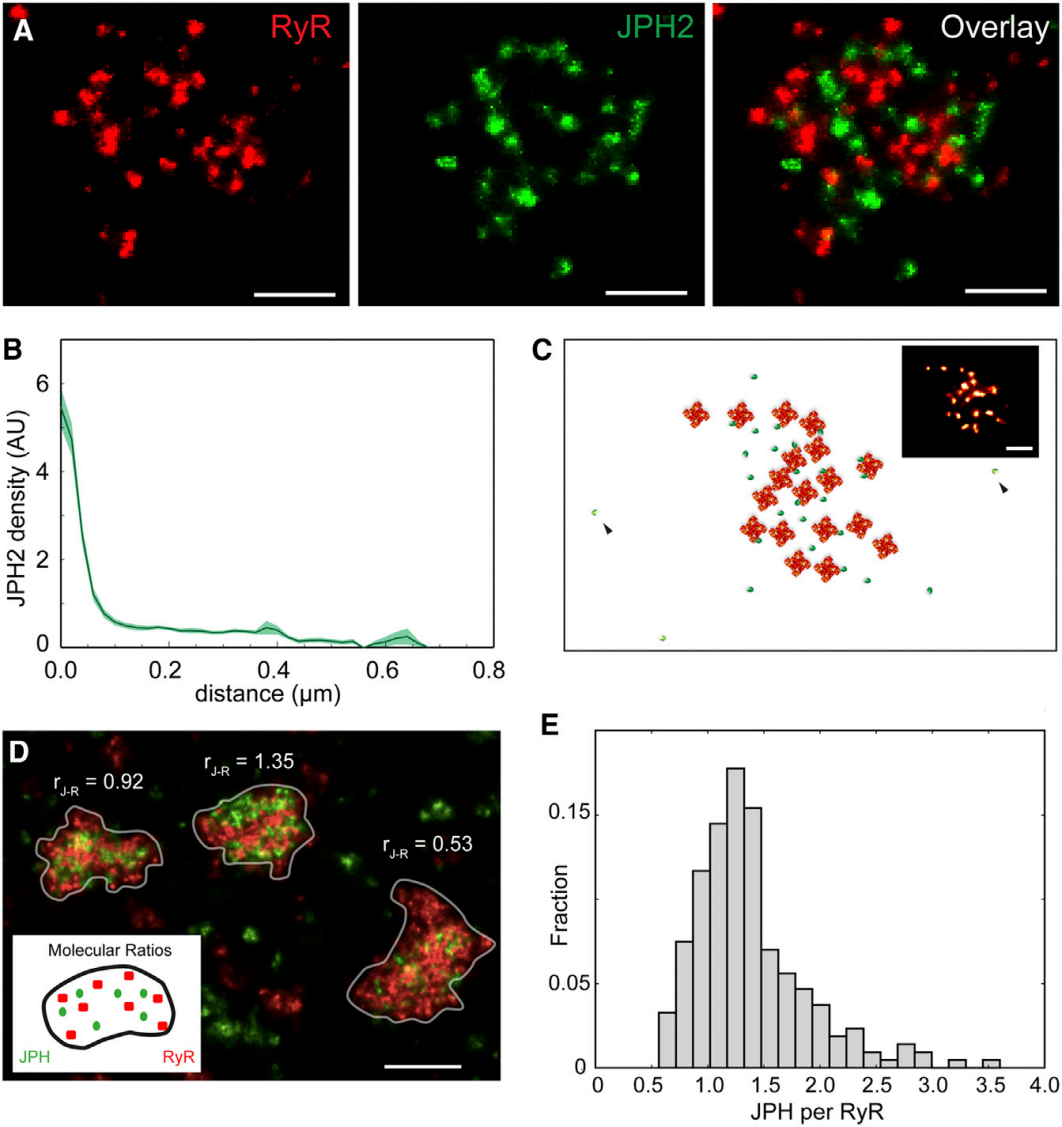
Exchange-PAINT of JPH2 Interaction with RyR in Peripheral Clusters (A) Example exchange-PAINT images of RyR (red), JPH2 (green), and their overlay (right). (B) Analysis of the JPH2 labeling density as a function of the distance from the centroids of RyR puncta resolved a high density of JPH2 markers within 50 nm of the RyRs. Error bars: SEM for n = 8 cells. (C) A schematic reconstruction of protein arrangements compatible with the DNA-PAINT data (inset): an irregular RyR array (orange) and populations of densely co-localized or bound JPH2 (dark green) and sparsely distributed JPH2 (light green). (D) Molecular ratios by exchange qPAINT of RyR and JPH2 determined from calibrated qPAINT data of RyR-JPH2 co-clusters (inset shows schematic), shown in three adjacent clusters exhibiting ratios between 0.53 and 1.35 JPH2/RyR. (E) Histogram illustrating the distribution of JPH2 to RyR ratios (mean 1.38, mode at 1.25, and a width of 0.5 (SD), n = 250 clusters (containing ≥15 RyRs), 3 cells, 2 animals). Scale bar in (A), 200 nm, (C), 100 nm, and (D) 250 nm.

The apparent irregular organization of RyRs within clusters prompted us to investigate how this nanostructure was compatible with co-clustering with JPH2, a regulator of RyR opening as well as a key molecular tether of junctions. Applying exchange-PAINT (i.e., sequential imaging of two populations of docking-strand markers) (Jungmann et al., 2014) revealed JPH2 within the same nanodomains as RyR clusters (Figure 5A, left), in co-clustered regions of JPH2 labeling (Figure 5A, mid). Overlay of the two images confirmed intimate tessellation of both RyR and JPH2 labeling densities (Figure 5A, right). Analysis of a large ensemble of clusters exhibited a high density of JPH2 labeling within just 50 nm of the centroids of RyR puncta (Figure 5B). By superimposing the RyR data with simulated DNA-PAINT images that contained a random spatially uniform distribution of JPH2 labeling, we confirmed that this increased density of JPH2 in the immediate regions adjacent to RyRs reflects a preferential (non-random) co-clustering behavior (Figure S5A). Visual comparisons (Figure S5B) were striking how this pattern of molecular arrangement was not apparent from dSTORM data. When investigated within cluster boundaries, as expected, dSTORM and exchange PAINT data reported arrangements between RyR and JPH2 that were quantitatively consistent. For example, the mean percentage of JPH2 overlapping within the area of the RyR cluster was similar between dSTORM and DNA-PAINT images (72.0% versus 70.3%; df = 11; p = 0.49 in Student’s t test).

Further to the high-density fraction of JPH2s close to RyRs (<50 nm), a non-zero JPH2 density was present at distances of >50 nm in Figure 5B. Examining the DNA-PAINT data with a Euclidean distance analysis revealed that this sparse subset of JPH2 occupied some of the gaps in the RyR arrays illustrated in Figure 4C (see Figure S5C for analysis). Taken together, these observations are highly suggestive of a sub-population of JPH2 molecules located within RyR clusters that are unlikely to be directly bound to RyRs (labeling at distances >50 nm), as shown schematically in Figure 5C, while another sub-population is close enough to be conceivably in a molecular complex with individual RyRs.

The high degree of co-clustering motivated an additional analysis that was enabled by the quantitative nature of qPAINT. By combining qPAINT analysis with the exchange-PAINT of RyR and JPH2, we determined molecular ratios of JPH2 to RyRs on a per cluster basis. The analysis was conducted for larger RyR clusters (>15 RyRs) that are likely located in larger junctions between surface and SR membranes. Figure 5D shows three typical clusters with ratios r_J-R_ of JPH2 to RyR between r_J-R_ = 0.53 and r_J-R_ = 1.35. It is notable that clusters with considerably different molecular ratios are observed in close proximity. An ensemble analysis (214 large clusters, n = 3 cells) revealed a distribution in which ratios varied between ∼0.5 and 3.5 with a mean at 1.38 JPH2 per RyR, a mode at 1.25 and a histogram width of 0.5 (quantified by the SD) as shown in Figure 5E. The molecular ratio determined *in situ* using qPAINT analysis, presents a measurement that is spatially more sensitive, particularly for analysis of nanodomains, than bulk (*in vitro*) biochemical analysis (e.g., immuno-co-precipitation).

## Discussion

The use of DNA-PAINT-based super-resolution imaging enabled fully quantitative imaging of clusters of RyRs in peripheral couplings of ventricular myocytes with molecular resolution. Improved localization precisions were critical in resolving individual RyRs and revealing clustering patterns at an unprecedented level of spatial detail. The data revealed apparently irregular arrangements of RyRs with considerable gaps within clusters and an apparent RyR density lower than expected for close-packed RyR arrays. The improved resolution showed that a sub-population of JPH2 was in molecular proximity to RyRs, compatible with being in a complex with individual RyRs, while per-cluster ratios of JPH2 to RyRs are highly variable.

### Clusters of RyRs at True Molecular Resolution

The data presented here show individually resolved RyRs and their arrangements into clusters using purely optical methods. Previous super-resolution imaging using dSTORM (Baddeley et al., 2009) and STED (Walker et al., 2014) had been able to resolve cluster outlines, but the lower resolution (∼50–60 nm full width at half maximum [FWHM]) prohibited determination of the density and arrangement of RyRs within individual clusters. Using DNA-PAINT, distinct puncta were observed that appear to arise from markers bound to individual RyRs. The puncta were highly reproducible in location and number; their observed distance is compatible with the expected ability of our DNA-PAINT imaging system to resolve objects ≤30 nm apart. With our labeling system and since RyRs as homo-tetramers provide several binding sites for antibodies (ABs), individual puncta may arise from more than one marker. A single RyR should not give rise to more than one punctum that can be spatially resolved since RyRs are less than 30 nm in diameter (Peng et al., 2016); if markers were bound in different places of a single RyR, these would be unlikely to be resolved into several puncta to give rise to the observed ≥40-nm neighbor spacings. The idea of each punctum representing a RyR is further supported by a second analysis that used the recent qPAINT analysis method (Jungmann et al., 2016). The qPAINT calibration measurements (Figure S4H) are compatible with a quantized distribution of groups of docking sites. The size of the quantal groups corresponded closely with individual puncta, compatible with a scenario where each punctum arises from markers complexed with a single RyR.

### DNA-PAINT Achieves Molecular Resolution with Relatively Low Experimental Complexity

Our data confirm that DNA-PAINT can provide very high spatial localization, with comparatively low optical complexity and demands on dye photo-physics, so that we could achieve high precision routinely in relatively complex biological preparations. As shown recently (Dai et al., 2016, Jungmann et al., 2014), the photon yields for imaging with DNA-PAINT improve the localization precision to <10 nm, and provided drift can be compensated, which we achieved with a transmitted light tracking system.

We made some modifications to the labeling system as described by Jungmann et al. (2014), notably by adding dyes to the docking strands so that successful staining and determination of suitable imaging areas was as straightforward as with conventional immuno-fluorescence imaging. This increased throughput and success rates in our experimental processing pipeline.

Another important advantage of DNA-PAINT is the ability to quantify the number of binding sites and calibrate the number of RyRs. qPAINT as described by Jungmann et al. (2016) relies only on binding between complementary DNA strands rather than models of dye photo-physics, which can be a source of uncertainty. We adapted qPAINT as an independent counting approach for RyRs and, in combination with exchange-PAINT, extended it to determine protein ratios between RyRs and JPH2. The DNA-based technology makes the scheme very flexible and delivered a robust quantitative super-resolution approach. The quantitative ratio analysis performed here, benefiting from the resolution to accurately recognize discrete nanodomains, provides information well beyond the sensitivity of classical analyses such as co-immunoprecipitation.

### RyR Cluster Properties

The key properties of RyR molecular expression as revealed by our data are summarized in Table 1. Both puncta counting and qPAINT analysis provided estimates of an RyR density within clusters of ∼0.3 RyRs/1,000 nm^2^. This is about 3 times lower than the dense packing in artificial lipid membranes where RyR density was ∼1 per 31.5^2^ nm^2^ = 1 per 992 nm^2^ (Yin and Lai, 2000). This is consistent with the apparent distance between RyR puncta in our data, which was >40 nm, rather than ∼32 nm as expected for dense packing. While it is possible that some RyRs were not detected because no markers were bound, such an effect is unlikely to explain the differences. We have verified that the marker system can detect proteins at considerably higher density, as demonstrated with CAV3, which was detected at a >3-fold higher density (Figure S6). In addition, we carried out simulations that suggest that our detection efficiency is high (discussed below).

**Table 1:** RyR Parameter Properties Estimated from DNA-PAINT Data

RyR cluster size^a^^,^^b^	8.81 ± 3.56 (n = 17 cells, 10 animals)
Nearest neighbor distance^a^^,^^c^	40.1 ± 0.9 nm (n = 1,802 clusters, 8 animals)
Average of distances to 4 nearest neighbors^a^^,^^c^	58.9 ± 0.9 nm (n = 1,802 clusters, 8 animals)
RyR packing density within cluster^a^^,^^d^	3,300 nm^–2^ (n = 391 clusters)
JPH2/RyR ratio^e^^,^^f^	1.38 ± 0.5 (n = 250 clusters, 2 animals)

a All measurements shown as mean ± SEM.

b Estimated from counting punctate densities of all RyR-labeled regions in TIRF DNA-PAINT images.

c From clusters containing ≥5 RyR.

d From the slope of the linear slope in scatterplot of cluster area versus qPAINT estimated RyR counts.

e Shown as mean ± SD of histogram.

f From qPAINT analysis of RyR and JPH2 exchange-PAINT data.

Consistent with the lower RyR density the average size of RyR clusters was smaller (∼64%) than earlier estimates, although the reduction was not in direct proportion to the lower density. This could result from over-estimates of cluster areas due to lower resolution in the original dSTORM data (Baddeley et al., 2009).

### RyR Cluster Morphology and RyR Biophysics

The distance between RyRs within clusters and the spatial arrangement of RyRs, the “cluster morphology,” is thought to play a major role for the excitability of a cluster by calcium (Walker et al., 2014, Walker et al., 2015). While EM methods can provide data with sufficient resolution, limits of contrast and throughput have meant that only a small number of clusters could be studied in this way (e.g., Asghari et al., 2014). The data provided here allow for the investigation of many clusters and their detailed RyR arrangement. Our analysis exploited the feature that peripheral couplings run parallel to the surface membrane (Franzini-Armstrong et al., 1999), which is attached to the coverslip and therefore the clusters are essentially flat in 2D. By restricting TIRF excitation to <100 nm from the coverslip interface, we ensured that deeper clusters with more complex orientations were excluded.

The distribution of puncta appeared irregular and could be mimicked using a model in which clusters were generated using placement of new RyRs in random directions and at a variable distance around a mean of ∼40 nm. Indeed, puncta NNDs, important in determining the ability of an open RyR to open adjacent RyRs (Groff and Smith, 2008, Winslow et al., 2006), had a strong mode at ∼40 nm, larger than the distance of ∼32 nm expected for dense packing of RyRs. This is consistent with recent electron tomography data, which showed that RyRs in clusters are not homogeneously packed.

A complication in analyzing distances between RyRs could arise from marker jitter, since we localize dye molecules conjugated to antibodies rather than the RyRs themselves. This could lead to an offset between reported marker positions and actual RyR locations that may be between 0 and ∼12.5 nm (Mikhaylova et al., 2015). Since >1 primary antibody may bind to a single RyR, the center of the resulting punctum may be quite close to the center of the RyR. As a result, the marker jitter may be small, but it is difficult to conclusively identify regularly packed sub-groups of RyRs. Notably, directly labeled primary antibodies do not resolve the jitter as primary antibodies may bind to RyRs with a variable offset depending on the location of epitopes on the large ∼27 × 27-nm protein. For this reason, our analysis focuses on NNDs as a robust lower limit estimate as marker jitter would only reduce the measured NNDs as compared to the true value.

The robustness of the NND estimate (with a prominent mode at ∼40 nm) is also largely unaffected by missed RyRs (Figures S2B and S2C). Simulations suggested that a large fraction of missed RyRs would be expected to increase the tail of the 4ND histogram substantially, more than we observed, our data being consistent with a >80% RyR detection. In support of this estimate, we confirmed that labeling conditions maximized RyR labeling: experiments with additional permeabilization with Triton X did not increase observed RyR density and we chose a saturating concentration of primary antibodies in the experiments.

The observations based on our data have several consequences for RyR cluster biophysics. The density of RyRs within clusters is lower than expected for close packing, which reduces the cross-signaling between RyRs. In addition, there are sizable gaps in RyR clusters, up to 150 nm. Our phenomenological cluster assembly model showed such gaps can arise randomly and do not require any templating mechanisms. Such gaps can dramatically alter the diffusion patterns of the cytoplasmic Ca^2+^ experienced by RyRs, which dictate the probability of concerted cluster activation (Walker et al., 2014, Walker et al., 2015) and termination of local Ca^2+^ release (Laver et al., 2013). Walker et al. (2014) predicted that cluster excitability may be lowered in clusters containing gaps of ∼50% of their internal area. Mathematical models need to be refined to capture the effect of such gaps on excitability using the data provided here.

### Molecular Mapping of JPH2-RyR Co-clusters

The ability to conduct exchange-PAINT enabled high-resolution imaging of the relative location of RyRs and the accessory protein JPH2. JPH2 has been shown to be important for the maintenance of the junctional membrane geometry (Takeshima et al., 2000). In addition, it has been proposed that JPH2 can bind to RyRs and modulate their gating (Beavers et al., 2013). Exchange-PAINT provided a method to image both targets with the same dye but using orthogonal docking and imager pairs so that chromatic aberrations can be eliminated. Distance-based analysis of the exchange-PAINT data showed that a sub-population of JPH2 was within molecular distances to RyRs, as seen by a >5-fold increase in JPH2 densities in areas ≤50 nm around RyR puncta. Such an increase would not be expected if JPH2 were randomly distributed across RyR clusters (Figure S5A). In conjunction with previous immuno-precipitation studies (Beavers et al., 2013), this observation is compatible with some JPH2 bound to RyRs. Direct comparison with dSTORM data showed that this observation critically depended on the higher resolution of DNA-PAINT.

We also found that JPH2 proteins were present in some of the larger gaps observed within RyR clusters, showing that the gaps are not merely reflecting a topological boundary but are genuinely part of peripheral junctions.

With the ability to quantify markers and proteins using calibrated qPAINT analysis, we also conducted qPAINT analysis of exchange-PAINT data to estimate JPH2-RyR protein ratios. The use of qPAINT for protein ratio analysis is a natural combination of the exchange-PAINT and qPAINT concepts. The images show that adjacent clusters often exhibit considerably different ratios. The variable RyR:JPH2 ratios from cluster to cluster add further complexity to regulating units of calcium signaling. Our observations provide a molecular basis for a mechanism in which RyR clusters can be locally regulated by varying the abundance of adjacent structural and inhibitory proteins, i.e., cluster level regulation for signaling in complex cells.

The relative variation between adjacent cluster ratios is clearly visually apparent (Figure 5D) and is independent of antibody binding efficiency. Similarly, the shape of the histogram shown in Figure 5E is an invariant feature of our data regardless of corrections for JPH2 antibody binding efficiencies. In general, the estimation of local protein ratios in structures of biological interest, here co-clusters of the two proteins, lends itself more readily to biological interpretation than often difficult to interpret co-localization values. The ratio of JPH2 to RyRs was critical in a JPH2 overexpression model in which RyR cluster sizes were greatly increased but excitability was stabilized by JPH2 (Munro et al., 2016).

**Figure 6:**
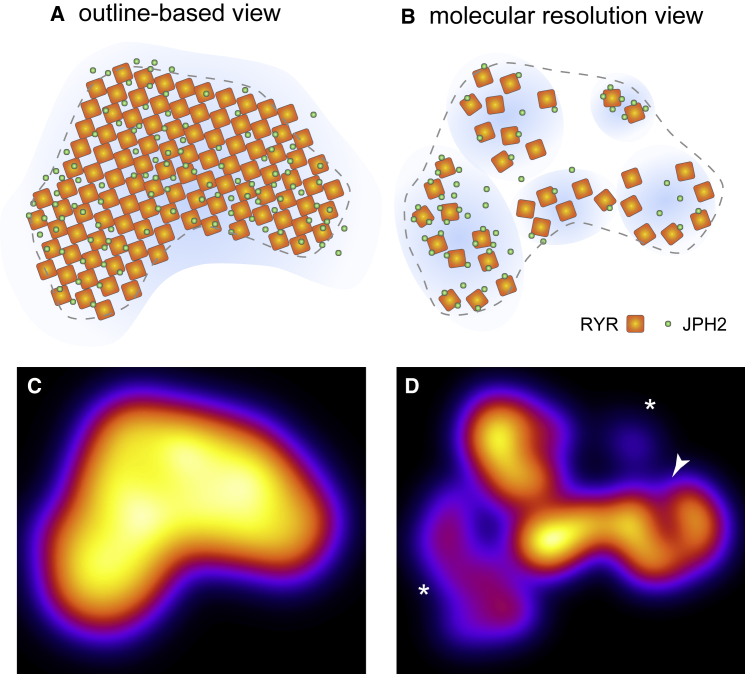
Schematic Comparison of Outline-Based and Molecular Scale Views of RyR Clusters (A and B) Schematic comparison of the outline-based view of RyR clusters (A) and the molecular-scale maps (B). The latter account for variable arrangements of RyRs, gaps in arrays and differential co-clustering with JPH2. (C) Schematic activation probability maps of clusters highlight uniform cluster activation probability in the regularly filled array compared to the heterogeneous activation of clusters (asterisks) and expected intra-nanodomain Ca^2+^ gradients (arrow) in the molecular-scale model.

The picture of RyR clusters and their regulation emerging from our data is schematically summarized in Figure 6. Where dSTORM informed outline-based views assumed a well-filled regular cluster with largely uniform activation properties, the molecular view shows a heterogeneous organization with differential inhibition by JPH2, compatible with a more complex regulation of RyR cluster activity.

### Limitations

The size and binding location of markers on RyRs currently limit the precision with which we can judge the arrangement of RyRs and identify close packed areas unambiguously; the development of small markers binding to well-characterized locations of RyRs should be assisted by recently improved 3D structures of the cardiac ryanodine receptor (Peng et al., 2016).

### Conclusions

The application of refined DNA-PAINT approaches to the molecular characterization of RyR clusters and JPH2-RyR co-clusters demonstrates the potential for molecular resolution quantitative imaging in complex biological samples. Using all optical methods, it is now possible to obtain data previously thought to be limited to the realm of electron microscopy. The optical data revealed that the density of RyRs in clusters is lower than expected for dense packing. Clusters follow an irregular assembly pattern compatible with spontaneous self-assembly and sizable gaps in clusters that will affect their calcium signaling. The high resolution of DNA-PAINT data also revealed a fraction of JPH2 in molecular proximity of RyRs and presents a super-resolution approach to *in situ* biochemistry to probe binding candidates in cells and tissues.

## Experimental Procedures

Further methods details are supplied in the Supplemental Experimental Procedures.

### Cell Preparation

Live myocytes were isolated from hearts freshly dissected from wild-type male Wistar rats according to a protocol approved by the Animal Ethics Approval Committee of the University of Exeter. The suspension of myocytes was filtered and allowed to attach within glass coverslip chambers prior to fixation with 2% paraformaldehyde (w/v) and immunocytochemistry.

### DNA-PAINT Probe Production

Both the “imager” and the “docking” strands (nucleotide sequence and terminal modifications specified in Table S1) were commercially synthesized by Eurofins UK. The two nucleotide designs of the P1 and P3 sequences were obtained from Jungmann et al. (2014). We opted for a direct thiolation to link a 5′ C6 amine of the docking strands and cysteines of the antibody. Respective docking strands were conjugated to either a goat anti-mouse immunoglobulin G (IgG) or a goat anti-rabbit IgG secondary antibody (Jackson ImmunoResearch, PA) or a primary antibody against RyR2, clone C3-33, using a Thunder-Link kit (Innova Biosciences, Cambridge) and spectrophotometric analysis to ensure an oligo: antibody conjugation ratio ≥1:1.

### Imaging Probes, Reagents, and DNA-PAINT Protocol

All primary antibodies used here were previously characterized for both immunofluorescence imaging and *in vitro* analyses of the targets RyR2, JPH2, and CAV3. The RyR2 antibody was a mouse monoclonal IgG (catalog no. MA3-916; Thermo Scientific, DE). See details on antibodies under Supplemental Experimental Procedures.

For dSTORM imaging, a secondary antibody conjugated to Alexa Fluor 647 was used and imaged in switching buffer. In DNA-PAINT imaging, the sample was immersed in a buffer (“buffer C” as in Jungmann et al., 2014) containing typically 200 pM of either an ATTO 655 or ATTO 550 imager strand, complementary to the docking strand linked to the secondary antibody that we aimed to image. The imager strands reversibly bind to the complementary docking strands. Using TIRF illumination the fluorophores of hybridized imager strands were imaged, while transiently immobilized and appeared in the image as transient fluorescent spots whose shape matches the point spread function (PSF) of the microscope in its focal plane. These events were recorded as a series of image frames (Figure S1).

### Image Acquisition

Both DNA-PAINT and dSTORM images were acquired with a modified Ti-E inverted fluorescence microscope (Nikon, Japan) and fully adjustable custom-built optical illumination and detection paths optimized for TIRF and single-molecule localization. The focus was actively stabilized with a feedback system that used transmitted light imaging and image correlation to keep the focus constant by driving a piezo focusing device appropriately (tolerance ≤ 30 nm), similar to a method described in (McGorty et al., 2013). This stabilization system also tracked lateral sample movement that was digitally removed during analysis by correcting event coordinates. The image data were acquired and analyzed in real-time by a quad-core PC using the open source Python Microscopy Environment (PyME) software. Analysis of localization data from the sCMOS camera was performed with algorithms that correct for non-uniform sCMOS pixel properties as described recently (Lin et al., 2017).

### Image Analysis

#### Basic Analysis and Grayscale Rendering

The frame data were analyzed in real-time (during acquisition) using the freely available PyME (http://python-microscopy.org/) developed by the consortium of co-authors. Localized event positions were rendered into a 16-bit grayscale tagged image file format (TIFF) image with a pixel scaling of 1 nm/pixel using an algorithm based on Delaunay triangularization (Baddeley et al., 2010) such that the pixel intensity was linearly proportional to the local density of localized markers, i.e., similar in its information content to a typical grayscale fluorescence micrograph albeit at higher spatial resolution.

#### Analysis of Punctate Nanoscale Densities

The punctate RyR labeling densities in the rendered images were detected using a custom-written analysis algorithm implemented in PyME. The centroids of puncta were used to count the number of observable RyRs within each cluster and to calculate the neighbor distances (e.g., Figure 3) through a Delaunay triangulation and to construct Euclidean distance maps, which were the basis for the distance-based density analysis of JPH2 labeling.

#### Area-Based Analysis of DNA-PAINT Images

The grayscale rendered DNA-PAINT images were also subjected to threshold-based analysis of RyR cluster areas as per previous dSTORM studies (Baddeley et al., 2009, Hou et al., 2015). 2D masks of the labeled regions were constructed and were used for computing the 2D area of RyR clusters and for performing a co-localization analysis between JPH2 and RyR in two-color DNA-PAINT data.

#### Statistical Methods

All mean and SDs of measurements presented in the manuscript were calculated using standard statistics routines in NumPy or Excel.

### Quantification Using qPAINT

#### qPAINT Adaptation

qPAINT analysis was used as a second approach to estimate the number of RyRs underlying the recorded DNA-PAINT signals by statistical analysis of the fluorescence time series data as described recently (Jungmann et al., 2016). Temporal fluorescence time courses were reconstructed from the localized event data after segmenting events based on clustering. qPAINT analysis exploits the fact that due to the reversible binding between docking and imaging strands, the number of docking sites in a region is inversely proportional to the mean “dark time,” i.e., the mean time between imager binding events, estimated through a histogram approach (Figure S4). A qPAINT index (i.e., inverse of the mean dark time) was estimated for each cluster or region, which were then calibrated by determining the qPAINT index of small clusters that contained only one or a few puncta.

#### Measurement of Protein Co-clustering Ratios in DNA Exchange-PAINT Series by qPAINT Analysis

The number *N*_*R*_ of RyRs and *N*_*J*_ of JPH proteins for each cluster by qPAINT analysis was estimated for larger RyR clusters (>15 RyRs, using the RyR cluster mask for segmentation of both RyR and JPH event data) in exchange-PAINT data. This included per channel calibrations of the RyR and JPH signals, respectively. The ratio *r*_*J-R*_ = *N*_*R*_ / *N*_*J*_ was calculated for each large cluster in this way.

### Simulation of Synthetic Data

Centroids of punctate RyR labeling densities were placed iteratively in either (1) a gridded organization at a fixed spacing (Figure S2A) or (2) randomly placed and at a variable spacing to the next nearest neighbor as described by a random sample from a normal distribution with a specified σ. The centroids of the puncta were then convolved with a 2D Gaussian model with a σ of 5 nm. The model was then used in the PyME software to generate single-molecule events within the labeled regions and rendered as grayscale images to match the imaging and localization parameters observed in the dSTORM and DNA-PAINT experimental data for simulation of dSTORM and DNA-PAINT imaging, respectively (Table S2).

## Supplemental Information

Document S1. Experimental Procedures, Figures S1–S6, and Tables S1 and S2

Document S2. Article plus Supplemental Information
