# Supplementary material for: True Molecular Scale Visualization of Variable Clustering Properties of Ryanodine Receptors
Source: Cell Rep. 2018 Jan 9;22(2):557–67. doi: 10.1016/j.celrep.2017.12.045 (PMC5775502; doi:10.1016/j.celrep.2017.12.045)
Supplement: Document S1. Experimental Procedures, Figures S1–S6, and Tables S1 and S2 [file mmc1.pdf]

Cell Reports, Volume *22*

Supplemental Information

True Molecular Scale Visualization of Variable Clustering Properties of Ryanodine Receptors

Izzy Jayasinghe, Alexander H. Clowsley, Ruisheng Lin, Tobias Lutz, Carl Harrison, Ellen Green, David Baddeley, Lorenzo Di Michele, and Christian Soeller

Supplementary Material

Supplementary Figures

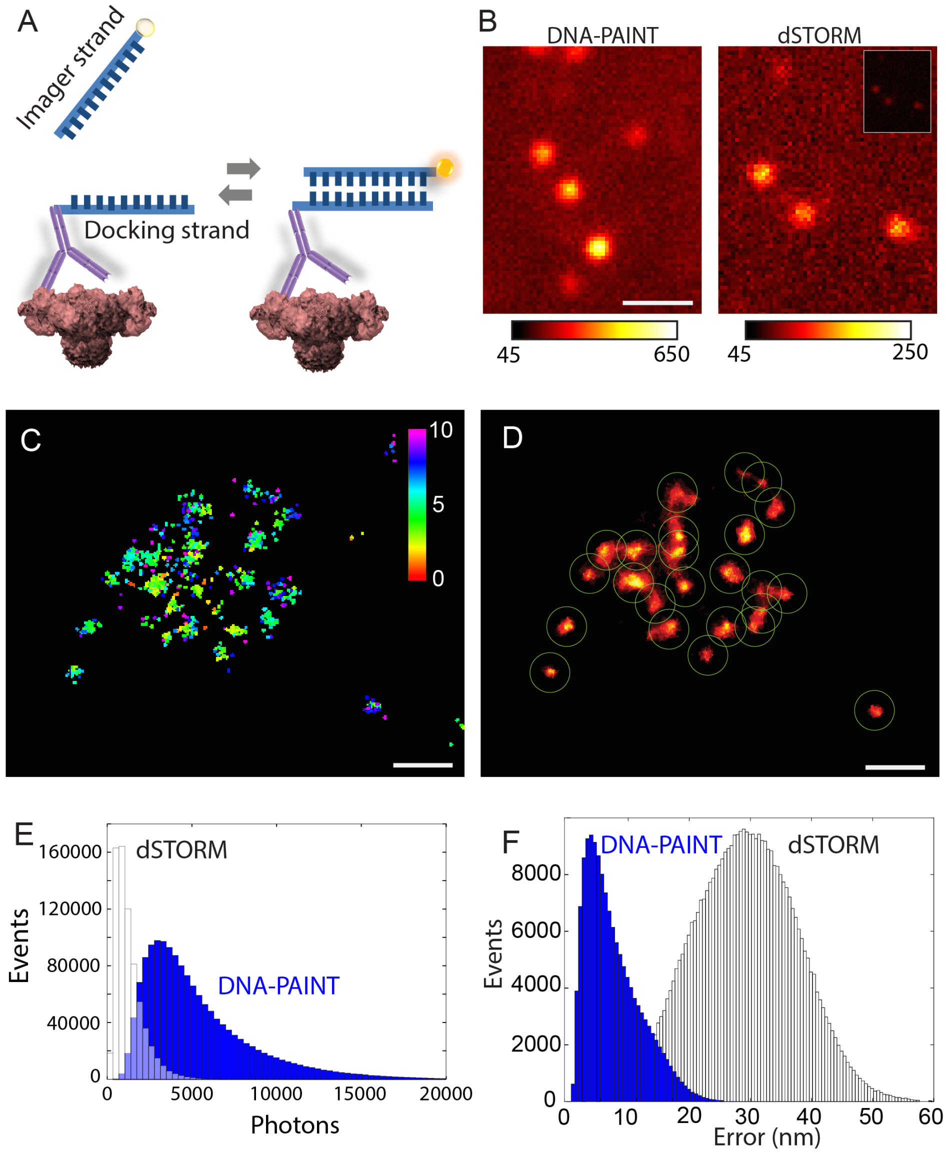

Supplementary Fig S1: DNA-PAINT implementation for improved RyR imaging. Related to Figure 1. (A) The DNA-PAINT principle works by the thermally-driven stochastic and transient hybridisation of the imager strand oligonucleotide conjugated to a fluorophore to the docking strand oligonucleotide marking the target RyR within the TIRF excitation field. **(B)** Binding events were visualised in a series of TIRF images as bright fluorescent spots (single molecule events, left) which were similar in their two-dimensional size and shape to dSTORM events (right), albeit brighter (detected photons per event). For comparison, a facsimile of a dSTORM frame is shown in the inset with the same colour-scaling as the DNA-PAINT image. **(C)** These events were used for localising the marker positions into a series of points whose spatial patterns were characteristic of nanometre-scale punctate structures (localisation error shown in colour-coded bar; scale in nm). **(D)** The point data were rendered into a 2D greyscale 32-bit image which shows the punctate morphology. To discretise these punctate densities, the centroids of each punctum were calculated with an automated puncta detection algorithm. Each detected punctum is indicated with centred circles of 50 nm radii. **(E)** A frequency histogram analysis of typical DNA-PAINT and dSTORM datasets revealed a 3-10 fold higher mode in the estimated total photons collected per event in DNA-PAINT. **(F)** The higher event intensity contributed to a reduced event localisation error in DNA-PAINT events as shown in the frequency histogram from a corresponding image series. Scale bars: (B) 1 µm, (C & D) 50 nm.

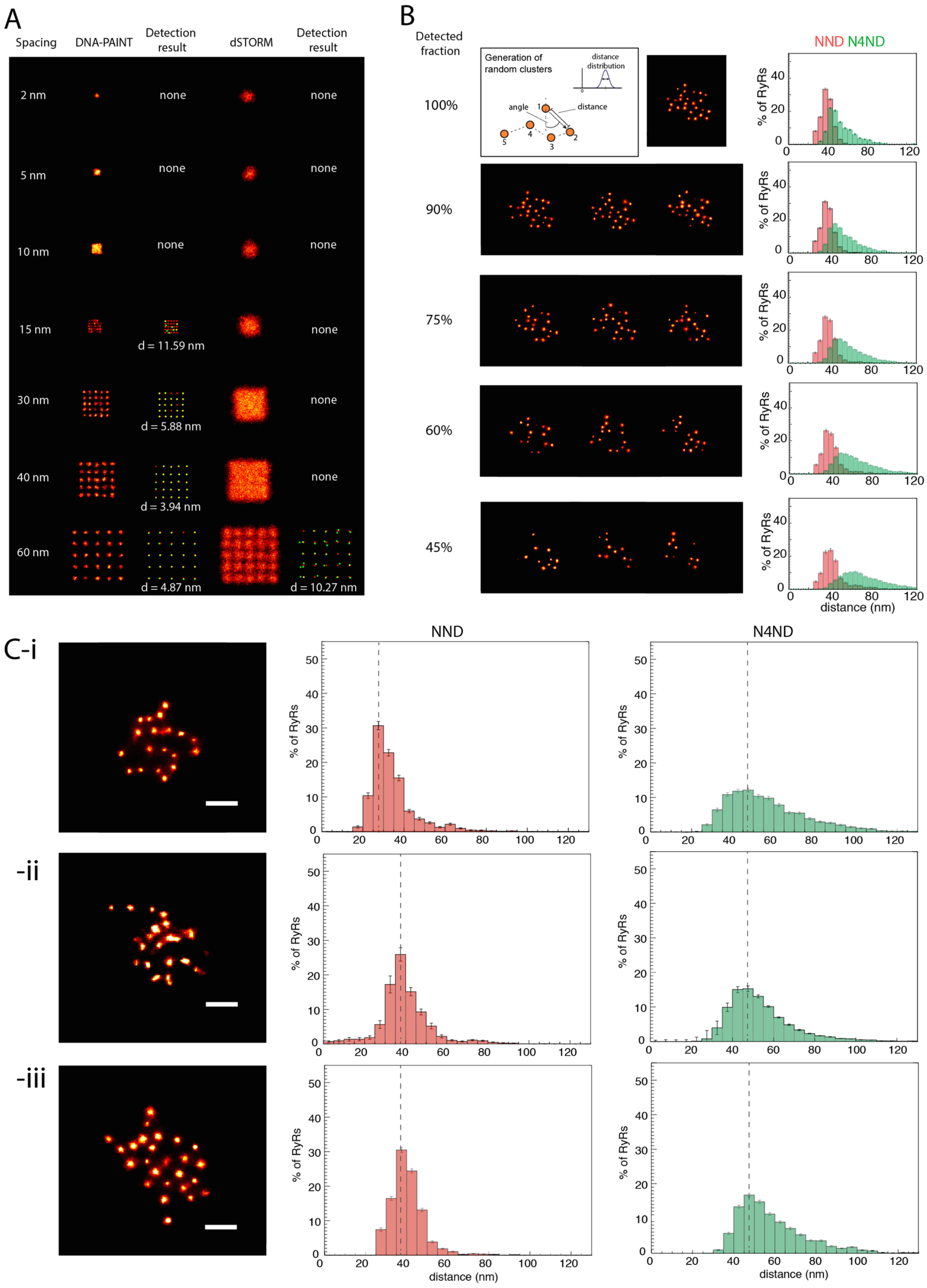

Supplementary Fig S2: Simulation of DNA-PAINT images of RyR clusters as a test model. Related to Figure 4. (A) Investigating the ability to resolve the protein distribution in clusters as a function of the localisation precision and associated imaging parameters utilising simulated target spacing patterns. Greyscale, simulated super-resolution DNA-PAINT (first column) and dSTORM (third) images of a cluster with targets on a square grid at regular spacings (specified on left) were simulated using imaging parameters as in Table S2. The square outline of the cluster is faithfully reported when the target spacing is >5x5 nm for DNA-PAINT parameters and >30x30 nm for dSTORM imaging parameters. The centroids of the punctate marker densities were determined with the method shown in Fig S1 (**green** circles) and overlaid with the centroids used for the simulation (**red**), 2nd and 4th columns. The markers are only resolvable when the marker spacing is >10x10 nm for DNA-PAINT and reliably detectable (efficiency ~84%) when the spacing is >15x15 nm. In dSTORM data, markers within the cluster are not resolvable until spacing is ~60x60 nm, yet including errors in centroid detection. The mean distance *d* between original versus detected target centroid is stated below each panel. **(B)** To investigate whether incomplete labelling of RyRs within a given cluster can distort the measured distribution, clusters with 25 RyRs at variable detection efficiencies (right

column) and nearest neighbour distances (NND) mean = 40 nm, sigma = 7.4 nm (second panel on top row), were simulated. Examples of Monte Carlo simulations with RyR detection efficiency of 90 %, 75 %, 60 % and 45 % are shown in middle panels. The corresponding NND (**red**) and the average of the distances to the nearest four neighbours (N4ND; **green**) are shown on right. Note how the reduction in the detection efficiency hardly altered the detected NND; however, a progressive broadening of the N4ND occurs with the mode being right shifted from 45 nm to 50, 60 and 65 nm, respectively. **(C-i)** To investigate the ability to distinguish between narrow RyR spacing and low labelling efficiency versus wider spacing but high labelling efficiency, a simulated cluster (left panel) from an ensemble generated with a mean RyR spacing of 30 nm and a detection efficiency of 50 % is shown. The NND distribution (middle panel) is only weakly affected by missed RyRs, the mode is still at ~30 nm. The N4ND distance measure is more sensitive and right-shifted. **(C-ii)** By contrast, our measurements of actual clusters exhibit a NND mode at ~40 nm, and N4ND mode at ~50 nm. **(C-iii)** The experimental observations are consistent with a cluster model that has a mean distance between nearest RyRs of 40 nm (middle panel), an example cluster is shown in the left panel. The N4ND (right panel) agrees well with experimental observations when a large detection efficiency >80% is assumed. Scale bars: 100 nm. Error bars: (B, C) NND & N4ND histograms – SEM for n=10 simulated clusters.

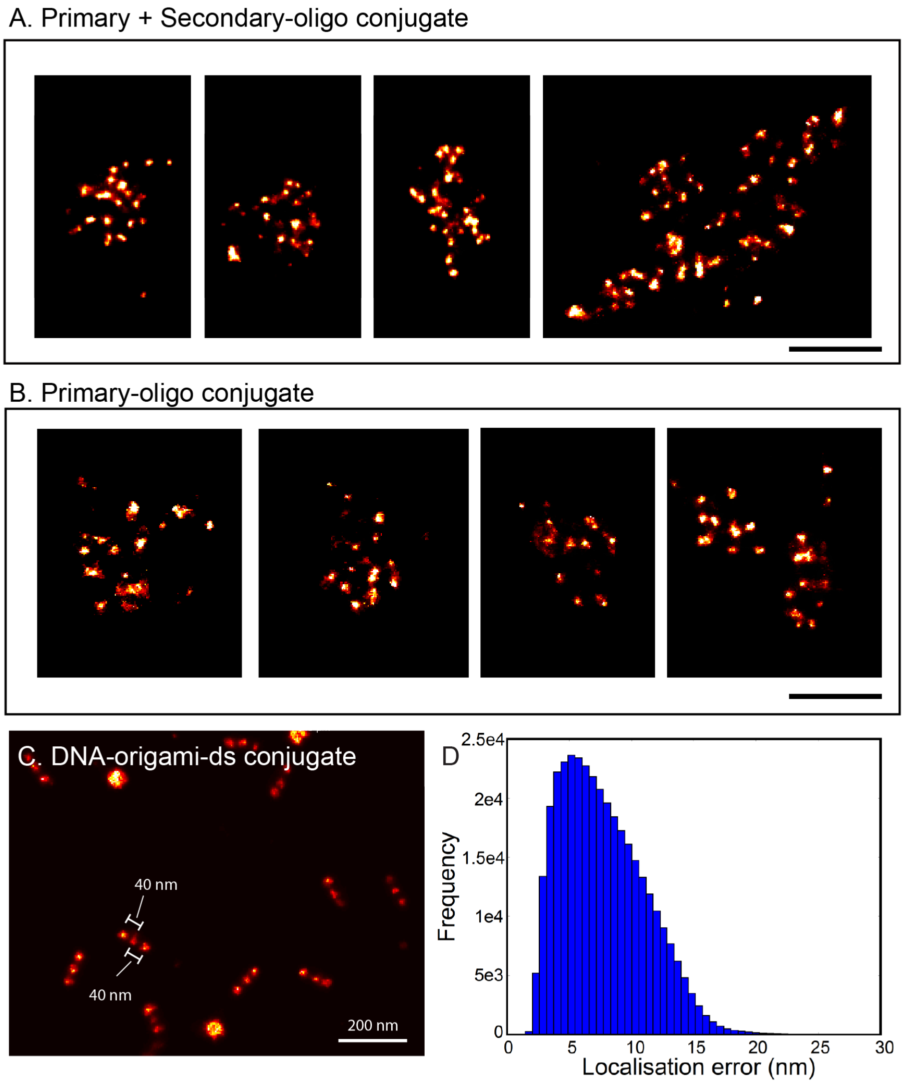

Supplementary Fig S3: Control experiments with DNA-PAINT samples. Related to Figure 2. To verify whether the morphology of the RyR cluster can be reproduced independently of the nature of the marker of the target protein (i.e. whether the morphology was dependent on the primary and secondary antibody interaction), a comparison was made between two labelling methods: **(A)** A gallery of RyR clusters resolved with DNA-PAINT where the RyRs were labelled with a monoclonal anti-RyR IgG primary antibody and then an anti-IgG secondary antibody conjugate of the docking strand. **(B)** A similar series of images of RyR clusters where the RyRs were labelled with the anti-RyR primary antibody directly conjugated to the docking strand revealed a similar punctate morphology, confirming that the punctate labelling densities observed in the two labelling approaches are independent of the size of the labelling complexes. **(C)** To demonstrate the resolvability of targets with 40 nm spacings with the DNA-PAINT protocol, we imaged DNA-origami molecular rulers in a Gattaquant 40R-HiRes slide. The larger round structures are additional, structurally different DNA-origami structures provided for drift correction. Drift correction was performed using transmitted light as described in the methods. **(D)** The estimated localisation errors of the events in the frame data used to generate these images were similar in magnitude to those in the experimental data in myocytes. Scale bars: 200 nm.

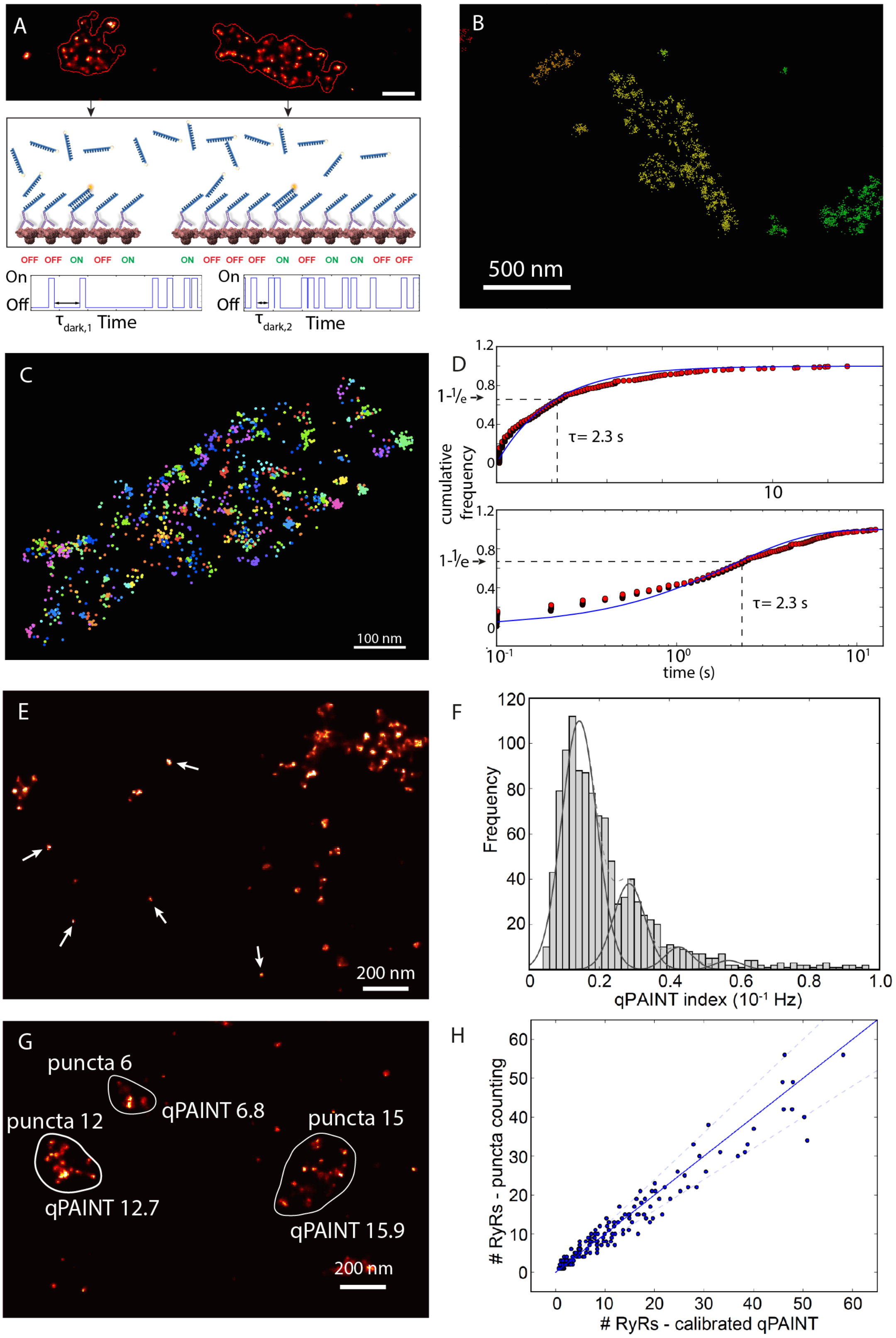

Supplementary Fig S4: Methodology of modified qPAINT analysis to verify RyR cluster size independently of the morphological analysis of the punctate labelling densities. Related to Figure 3. (A) qPAINT analyses the temporal properties of single molecule event detections recorded at a given cluster. Two clusters are shown, one larger than the other. The rate at which imagers bind to RyRs in a cluster is proportional to the number of RyRs in the cluster; the average dark time between detected binding events is measured (lower panels) which shortens as more labelled RyRs provide binding sites for imagers in solution. The RyR numbers are proportional to the inverse of the dark time but to obtain absolute numbers a calibration must be performed to determine the constant of

proportionality. **(B)** A view of detected events (dots) in a section of a DNA-PAINT data set. Events are segmented and coloured according to the cluster they belong to. **(C)** A single cluster is shown; events belonging to this cluster are coloured according to the time at which they were detected. **(D)** From the events belonging to a cluster and their time stamps (frame number) a cumulative histogram of dark times is reconstructed (red dots) which is fit with the expected distribution for a first order binding reaction (see Methods). The average dark time is located at the 1-1/e point of the curve, here $\tau = 2.3$ s. Top panel: linear time scale; bottom panel: logarithmic time scale. **(E)** Small clusters, visually containing only one or a few puncta (arrows), were identified by apparent cluster area (cluster area < 10,000 nm^2) and their dark times and qPAINT indices were determined. **(F)** The qPAINT index (inverse of the measured dark time) histogram exhibited peaks at multiples of a qPAINT index of $0.14 \cdot 10^{-1}$ Hz. We visually confirmed that the clusters corresponding to the fundamental peak typically contained a single punctum. The calibration value was used to calibrate the corresponding data set and obtain estimated RyR numbers for each cluster as the ratio of the cluster qPAINT index q_i and the calibration value q_{i1}. Calibrations were conducted for each data set using a similar in-situ calibration. **(G)** A small section of a DNA-PAINT image of RyR labelling shows three clusters and the comparison of RyR number estimates obtained by puncta counting and calibrated qPAINT analysis indicates good agreement. **(H)** The correlation between RyR cluster sizes determined by puncta counting and qPAINT analysis is relatively tight. The solid line is the line of identity, the dashed lines show ratios of 1.2 and 0.8, respectively. Each data point represents a single cluster.

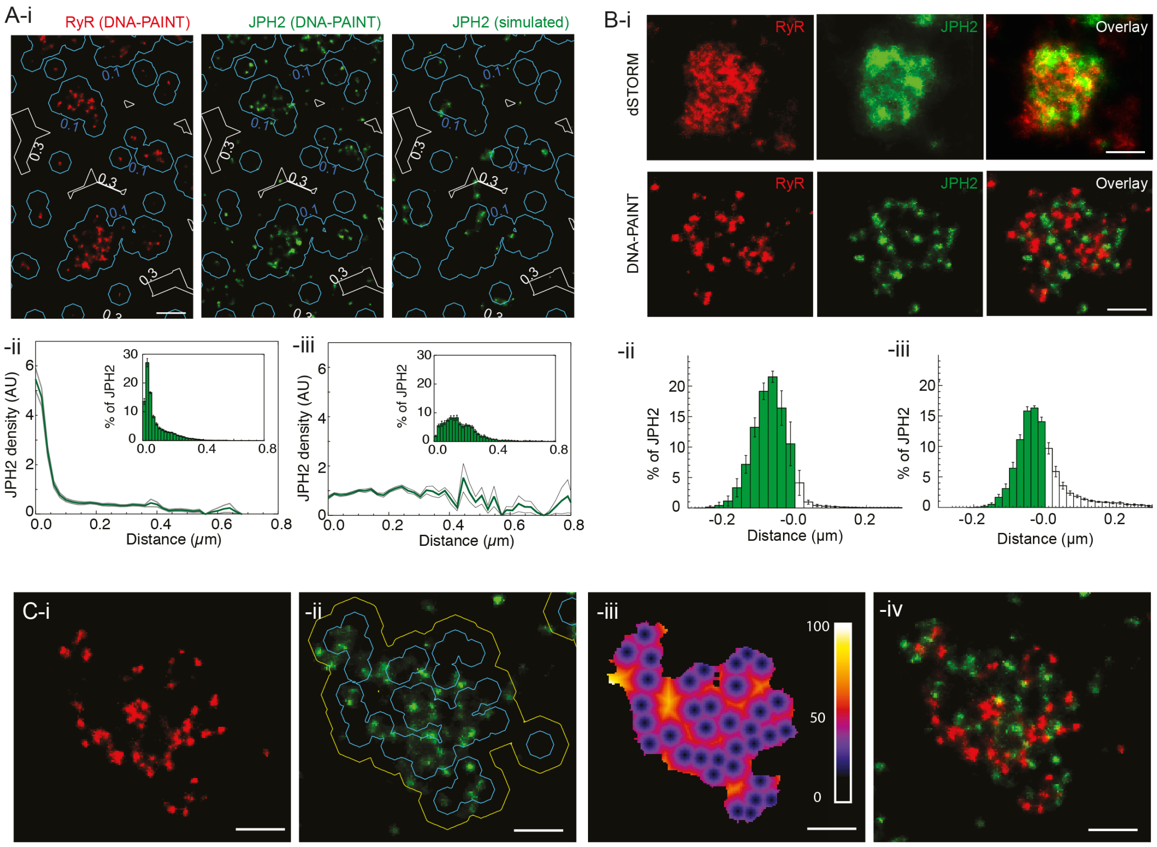

Supplementary Fig S5: Analysis of the nanoscale co-localisation of JPH2 within RyR clusters. Related to Figure 5. (A-i) A distance-based co-localisation was applied (as in Jayasinghe et al. 2012) to quantify the nature of the co-localisation between RyR (left) and JPH (centre). To verify whether the observed JPH2 localisation distribution showed preferential co-clustering with RyR, we also simulated a DNA-PAINT image of a randomly distributed marker population of JPH2 whose 2D position was assigned in the image based on a random uniform distribution (A- right). The Euclidean distance map constructed from the centroids of the RyR puncta (0.1 μm and 0.3 μm contours) were overlaid with the DNA-PAINT images which showed that both RyR (left panel) and experimental JPH2 distributions (middle panel) were intimately co-clustered within 100 nm distance bands while the simulated JPH2 data (right panel) showed this to a lesser extent. **(A-ii)** Histogram analysis of the density of JPH2 labelling as a function of the distance from the centroids of the single RyR puncta shows a high density of JPH2 markers within 50 nm of the RyRs. At distances beyond 100 nm, the residual density was approximately uniform but 5-10 times lower. Analysis of the percentage of the integrated labelling density as a function of this distance scale (inset) showed that >86 % of all the JPH2 events were localised within 200 nm of the RyR puncta. **(A-iii)** The same density-distance analysis performed by replacing the experimental JPH2 image with the simulated JPH2 image where JPH molecules were randomly placed exhibited essentially a uniform density distribution, as expected. The random placement of JPH2 diminished the high percentage of JPH2 organised closely to RyR (only ~58% of the JPH2 labelling localised within 200 nm) confirming that the experimentally observed JPH2 distribution is not compatible with JPH2 random placement relative to RyRs. **(B-i)** From dSTORM (top row) and DNA-PAINT (second row) data of RyR (red) and JPH2 labelling (green; overlays also shown on left), we analysed the percentage of <u>integrated JPH2 labelling</u> as a function of the Euclidean distance from the edge of each RyR-labelled region. Histograms reflect analyses performed on **(B-ii)** dSTORM and **(B-iii)** DNA-PAINT data. The shaded area (distances <0.0) of the percentage histogram of the integrated JPH2 labelling dSTORM data plotted as a function of the distance from the <u>edge</u> of each RyR cluster reports 72.0 ± 2.5% (n=5 cells; 2 animals) of this labelling within the RyR mask (see also previous analysis by Jayasinghe et al. 2012). In the DNA-PAINT analyses the percentage histogram equivalent to above, from an analysis based on the cluster edge, revealed a smaller total fraction (~70%) of JPH2 now co-localising with RyR. This decrease is expected due to the higher resolution in the DNA-PAINT data but we note that the dSTORM data do not detect the increase in JPH2 density close to RyRs, consistent with a lower effective resolution. **(C-i)** Given the non-uniform arrangement of RyR (Fig 3), we examined whether any of the JPH2 densities could be localised to the regions within the RyR cluster that were devoid of RyRs. Shown, are exchange-PAINT images of RyR (red) and **(C-ii)** JPH2 (green) acquired from a rat ventricular myocyte. Overlay of the JPH2 image with a contour map (50 nm in blue; 100 nm in yellow) of the Euclidean distance from the centroids

of RyR puncta illustrates that a large fraction of the JPH2 are localised within the 50 nm bands (i.e. most likely bound to RyRs). A smaller fraction was observed between the yellow and blue contours signifying JPH2 which are resident to the junction, but nevertheless unlikely binding partners of RyR. **(C-iii)** The latter population of JPH align strongly with the yellow regions of the Euclidean distance colour map (scale in nm) reporting continuous regions devoid of RyR. **(C-iv)** Domains of potentially specialised localisation of JPH2 are also notable in the two-colour overlay of DNA-PAINT images unlike the dSTORM images (panel B-i). Scale bars: 200 nm. Error bars: (Aii, iii) SEM for n=8 cells; (Bii) SEM for n=5 cells; (Biii) SEM for n=8 cells.

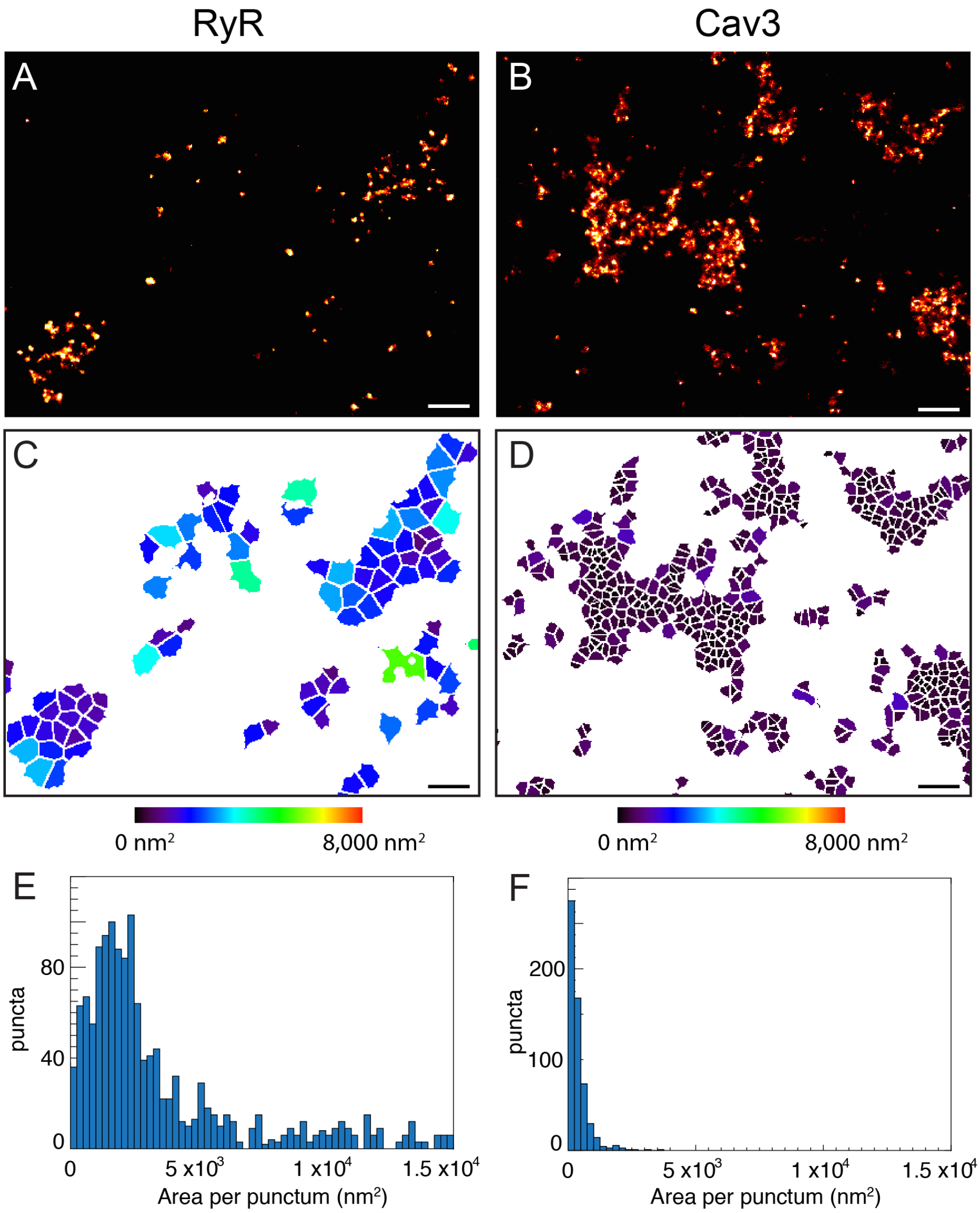

Supplementary Fig S6: The packing distances of antibody labelling densities are not limited by the labelling system. Related to Figure 6. To examine whether the apparent marker binding density or the resolvable marker spacings are compatible with high density protein distributions, we examined DNA-PAINT data labelled against the densely-organised plasma membrane protein Caveolin-3. **(A)** Typical DNA-PAINT images of peripheral cluster labelling of mouse monoclonal anti-RyR2 antibodies. **(B)** DNA-PAINT images of mouse monoclonal anti-Caveolin-3 antibodies in similar regions. Note the punctate labelling morphology in both label distributions. To quantify the proximity of packing, the centroids of the punctate densities were computed (as in Fig S1D) and subjected to Voronoi partitions to visualise the "areas" per punctum for both the **(C)** RyR and **(D)** Caveolin-3 label distribution. Each of the Voronoi partitions is coloured according to the size of the area around a punctum (see calibration). The frequency distributions of the Voronoi partition areas for **(E)** RyR antibodies and **(F)** Caveolin-3 antibodies confirm the smaller partitions in Caveolin-3 images compared to RyR. This observation showed that our methodology was able to detect higher punctate labelling densities than observed with RyR markers and that the observed RyR morphology or distance measurements are unlikely to be limited by marker access to the target or the resolution of the DNA-PAINT images. Average densities of puncta in RyR data was one per 4314.87 ± 1332.47 nm^2 while that in caveolin-3 was one per 1466.95 ± 455.5 nm^2. Scale bars: 50 nm.

Supplementary Tables

DNA-PAINT imaging channel	Imager strand	Docking strand
561 nm excitation/ orange emission for P1 configuration	5'–CTAGATGTAT-3'-ATTO550	C6-amine-5'-TTATACATCTA–3'-Cy5
642 nm excitation/ far red emission for P1 configuration	5'–CTAGATGTAT-ATTO655	C6-amine-5'-TTATACATCTA–3'-Cy3.5
561 nm excitation/ orange emission for P3 configuration	5'–GTAATGAAGA-3'-ATTO550	C6-amine-5'-TTTCTTCATTA–3'-Cy5
642 nm excitation/ far red emission for P3 configuration	5'–GTAATGAAGA-ATTO655	C6-amine-5'-TTTCTTCATTA–3'-Cy3

Table S1: The nucleotide sequence and terminal modifications of the P1 and P3 'imager'/'docking' strand pairs used for specific imaging configurations. Related to Experimental Procedures.

**Parameter**	**dSTORM**	**DNA-PAINT**
Events per pixel	1.0	1.0
Event intensity (photons)	1000	8000
Event background (photons)	200	600
Event duration (frames)	3	6
Events per marker	2	5
Number of frames	30000	10000
Rendering localisation error filter threshold	30 nm	10 nm

Table S2: The parameters adopted for generating synthetic localisation data in Python Microscopy Environment software. Related to Figure 4 and Supplementary Figure 2.

Supplementary experimental details

Animals, sample preparation and immunocytochemistry

Male Wistar rats weighing 250-350 g were euthanised humanely with lethal dose (100 mg per kg of body weight) of sodium pentabarbitone according to a Schedule 1 protocol in compliance with the 1986 Animals (Scientific Procedures) Act, approved by the Animal Ethics Approval Committee of the University of Exeter. The hearts were quickly excised, cannulated at the aorta and retrogradely perfused with calcium (Ca^{2+})-free Tyrode's solution using a Langendorff perfusion protocol described previously (1). After 5 minutes, the perfusate was switched to a Tyrode's solution containing 200 μM $CaCl_2$, 1 mg/mL Collagenase-II (CLS2, Worthington Biochemical, NJ) and 0.1 mg/mL Protease-I (Sigma-Aldrich, MO). Following 10-15 minutes of perfusion, the ventricles were dissected into fresh Tyrode's solution containing 200 μM $CaCl_2$, diced with sharp scissors and gently triturated to liberate isolated ventricular myocytes. The suspension of myocytes was filtered and transferred in aliquots into the custom-made imaging chambers whose bottom consisted of a No 1.5 glass coverslip. Coverslips were previously cleaned with concentrated NaOH in methanol, rinsed with fresh distilled water and coated overnight at room temperature with a laminin solution (11.9 μg/mL, diluted from #23017-015, Life Technologies) to allow strong attachment of myocytes. After incubation in the chambers for 90 minutes at 30° C, the myocytes attached to the coverslip were fixed by replacing the Tyrode's solution and excess cell suspension with 2 % (w/v) paraformaldehyde (Sigma-Aldrich) in phosphate buffered saline (PBS) at pH of 7.4. After incubating in room temperature for 12 minutes, the fixative was replaced with fresh PBS and allowed to wash for another 12 minutes. This washing was repeated once more before storing the chambers in fresh PBS containing 0.1% sodium azide at 4° C until the immunocytochemistry step.

The immuno-labelling of specific targets of fixed cells was performed according to a protocol described previously (1). Fixed myocytes which were pre-adhered to laminin-coated chambers were briefly permeabilised with 0.1% Triton-X-100 (Sigma-Aldrich) diluted (v/v) in PBS for 10 minutes and then blocked with PBS containing 10% normal goat serum (Life Technologies) for 1 hour at room temperature. The primary antibody was diluted in an incubation buffer containing 1x PBS, 0.5% bovine serum albumin (w/v; Sigma-Aldrich) and 1% normal goat serum and applied overnight at 4° C. Samples were washed three times with fresh PBS at 30 minute intervals and incubated for two hours at room temperature. Secondary antibodies were dissolved in PBS containing 1 mM ethylenediaminetetraacetic acid (EDTA) and applied for 2 hours at room temperature before three further washing steps.

DNA-PAINT probe production

Both the 'imager' and the 'docking' strands (nucleotide sequence and terminal modifications detailed in Table S1) were commercially synthesised and HPLC purified using the custom DNA oligos service by Eurofins UK. The two nucleotide designs of the P1 and P3 sequences were obtained from (2).

Rather than using a biotin-streptavidin linker (which is large and can give rise to undesirable non-specific labelling with endogenous biotins in the myocytes) as done in the original DNA-PAINT study (2), we opted for a direct thiolation which links a 5' C6 amine of the docking strands and cysteines of the antibody. In addition, the conjugated docking strands contained a 3' fluorophore which has the advantage that the conjugated antibody markers carry a fluorochrome, allowing us to confirm positive staining. This fluorophore does not interfere with later DNA-PAINT imaging since generally the 3'-fluorophore was selected spectrally distinct from the imager fluorophore. In rare cases where there was overlap between docking strand fluorophore and imager fluorophore emission a period of intense illumination was used to remove any background from docking strand fluorophores by permanent photobleaching before commencing DNA-PAINT acquisition. Respective docking strands were conjugated to either a goat anti-mouse IgG or a goat anti-rabbit IgG secondary antibody (affinity purified, azide-free form from Jackson Immunoresearch, PA) using a Thunder-Link® kit (Innova Biosciences, Cambridge). In some experiments a primary antibody against RyR2, clone C3-33 – see below, was conjugated directly to DNA-PAINT docking strands. The conjugated antibody was purified from unconjugated docking strands by use of a Thunder-Link® kit-based precipitation step and centrifugation at 13,000g. The relative concentration of the antibody and the docking strand (by the 3'-fluorophore) in the conjugate were determined with the use of a NanoDrop 2000 spectrophotometer (Thermo Scientific). Only antibody samples with an oligo : antibody conjugation ratio ≥ 1:1 were used for sample labelling.

Antibodies

The mouse monoclonal anti-ryanodine receptor-2 IgG from Clone C3-33 (Cat# MA3-916; Thermo Scientific, DE) was used for labelling the ryanodine receptors in the fixed cardiomyocytes. This antibody is well characterised for its high specificity of binding(3) and has been established as one of the consistently reliable probes for diffraction-limited (1, 4) and super-resolution microscopies (5, 6) of RyR2. As a qualitative comparison, a monoclonal pan RyR antibody of clone 34C (Cat# MA3-925; Thermo Scientific) was used to obtain independent DNA-PAINT images of peripheral RyR2 distributions in the myocytes. For staining Caveolin-3, a mouse monoclonal antibody

from BD Transduction (Cat# 610421) was used while a custom-made rabbit polyclonal antibody described previously (7) was used against Juctophilin-2 (JPH2). Alexa Fluor 647 (Cat# A-21236; Thermo Scientific) secondary antibodies were used for dSTORM experiments and imaged in PBS based switching buffer containing 10% (w/v) glucose, 100 mM 2-Mercaptoethylamine, Glucose oxidase (0.5 mg/mL) and catalase (0.05 mg/mL), all from Sigma.

Details of imaging apparatus

Both DNA-PAINT and dSTORM images were acquired with a modified Nikon Ti-E inverted fluorescence microscope (Nikon, Japan) and fully-adjustable custom-built optical illumination and detection paths, see also Fig.1-S1. These custom-optics allowed the laser to be conveniently focused into the sample in an angle either exceeding the critical angle (for TIRF imaging) in DNA-PAINT experiments or at a shallower angle for oblique illumination (8) for dSTORM experiments. This produced an ~20 µm-wide illumination spot which was sufficiently large to observe the single molecule events (PAINT or dSTORM) from peripheral couplons near the surface of the cell which was in contact with the coverslip as performed previously (5). The laser was focused into the sample with a 1.49NA 60x oil immersion objective fixed onto a piezo focus z-drive (P-725 Physik Instrumente, Germany) which was mounted rigidly within a custom-made aluminium bracket to minimise any thermal drift of the focal plane.

Illumination for the tracking system that stabilises residual thermal drift was provided by the white light bulb of the microscope condenser illuminator, similar to a method described in (9). A band-pass filter (Semrock FF02-447/60) was inserted to select only the blue light which is decoupled from the fluorescence path by a high-flatness dichroic (Semrock FF660-Di02). During data acquisition, images using the blue transmitted light are captured by a tracking camera (Thorlabs DCC3240N). A matching band-pass filter is inserted in front of the camera to clean up any stray light from the laser. Using image correlation to measure focus changes the focus was kept constant by driving a PiFoc piezo focusing device appropriately (tolerance <= 30 nm). The focus stabilisation system also tracked lateral sample movements which were not actively corrected during acquisition but instead digitally removed during analysis by subtracting the recorded lateral drift time course from event coordinates.

Prior to DNA-PAINT imaging, the presence of the primary and secondary antibodies was confirmed by the fluorescence of the docking-strand fluorophores (Cy5, Cy3 or Cy3.5) with the use of a multi-spectral epi-fluorescence exciter (CoolLED). For PAINT or dSTORM imaging, either a 200 mW 561 nm (Cobalt Jive-200) laser or a 140 mW 642 nm (Omicron) laser were used. The laser excitation intensity was controlled with a computer driven neutral density filter wheel (Thorlabs) and accousto-optic modulators built into the laser modules. Single molecule images were acquired with a scientific-CMOS camera (Andor, UK) operated with integration times of either 100 ms (PAINT experiments) or 25 ms (dSTORM). The image data were acquired and analysed in real-time by a quad-core PC using the open source custom-written Python Microscopy Environment (PyME) software (freely available at http://python-microscopy.org/) Analysis of localisation data from the sCMOS camera was performed with algorithms that correct for non-uniform sCMOS pixel properties which are implemented in PyME as described recently (10).

Image analysis

Basic analysis and greyscale rendering: The frame data of DNA-PAINT single molecule events were analysed in real-time (during acquisition) using the PyME software developed by the consortium of co-authors (11). The analysis included the detection of single molecule events and least-squares fitting of a 2D Gaussian to localise their sub-pixel scale centroid as well as removal of the effect of sCMOS chip pixel non-uniformities by the use of camera-maps (10). The point data of the localised marker positions from a sub-series of 10,000 frames were selected for further analysis and events lasting more than one frame (tracked in consecutive frames to be within the localisation precision) were coalesced to minimise localisation errors – except for qPAINT analysis. For qPAINT analysis, as described in more detail below, un-coalesced single frame events were used. The event positions were then rendered into a 16-bit greyscale TIFF image with a pixel scaling of 1 nm/pixel using an algorithm based on Delaunay triangularisation (12). In these images, the pixel intensity was linearly proportional to the local density of localised markers, i.e. similar in its information content to a typical greyscale fluorescence micrograph albeit at higher spatial resolution.

Analysis of punctate nanoscale densities: The punctate RyR labelling densities in the rendered images were detected using a custom-written analysis algorithm implemented in PyME. This involved a smoothing of the images (kernel size of 3.0 pixels) and a multi-threshold detection of labelled regions. In a second round, an automatically-defined region of interest (ROI) capturing each of the punctate densities was least-squares fitted with an adaptive two-dimensional (2D) Gaussian model to determine the sub-pixel coordinates of the 2D centroid of each punctum. These centroids were used to count the number of observable RyRs within each cluster and to calculate the neighbour distances (e.g. Fig 3) through a Delaunay triangulation implemented with custom-written programs in the

IDL or Python programming language similar to previously described analyses (6). The centroids from RyR images were also used to construct Euclidean distance maps which were the basis for the distance-based density analysis of JPH2 labelling.

Area-based analysis of DNA-PAINT images: The greyscale rendered DNA-PAINT images were also subjected to threshold-based analysis of RyR cluster areas similar to previous dSTORM studies (5, 6). Using custom-written programs implemented in IDL or Python, a global threshold which encapsulated 80% of the total labelling fraction above background was adopted to generate a mask of the RyR labelled area. By filling in all dark 'holes' in connected mask regions that were smaller than 5000 nm^2, we robustly obtained a 2D mask of the overall RyR cluster area. These 2D masks were used for computing the 2D area of RyR clusters and for performing a co-localisation analysis on the overlap between JPH2 and RyR in two-colour DNA-PAINT data based on an algorithm detailed previously in the supporting material of (1). Briefly, this included the computation of two Euclidean distance maps, one containing the Euclidean distances in the regions outside of the RyR cluster (in relation to the cluster edge) and the other in the regions within the RyR cluster mask (also in relation to the cluster edge). Histograms of the integrated labelling of the second channel (e.g. JPH2) show labelling that is localised within the cluster ('negative' distances, shaded) and that outside of the cluster (positive distances, see e.g. supplementary Fig S5-B).

Quantitative analysis of DNA-PAINT data by qPAINT

qPAINT analysis of DNA-PAINT data is based on the idea that the temporal statistics of imager binding contains information about the number of binding sites (docking strands) given that the transient binding between individual imaging and docking strands follows first order binding kinetics. Specifically, the measured dark time τ_D is related to the number of binding sites N_B via

$$N_B = \frac{1}{k_{on} c_i \tau_D}$$

where k_{on} is the on-rate of imager to docking strand binding and c_i is the imager concentration. When using markers that contain α docking strands per marker and β markers bind on average to a single receptor, the number of receptors N_R is also proportional to τ_D^{-1}

$$N_R = \frac{N_B}{\alpha\beta} = \frac{1}{\alpha\beta k_{on} c_i} \frac{1}{\tau_D} = c' q_i$$

where we have lumped all constants into the constant c' and termed the inverse of the dark time the "qPAINT index" $q_i = \tau_D^{-1}$. N_R can be obtained from the uncalibrated qPAINT index q_i if the calibration value q_{i1} can be measured, where q_{i1} is the qPAINT index value for a single receptor. Inspection of the formulae then yields

$$N_R = \frac{q_i}{q_{i1}}$$

We pursued the calibration strategy as derived above to obtain RyR number estimates. The *in-situ* calibration was performed for each DNA-PAINT series since it is difficult to control the imager concentration c_i accurately between experiments. Care was taken to only analyse DNA-PAINT sequences that exhibit a constant event rate over the time window that was analysed for qPAINT quantification.

Measurement of dark times and qPAINT indices. Cluster event segmentation was performed based on cluster segmentation of rendered images. Cluster masks were obtained from rendered images as described in the section "area-based analysis of DNA-PAINT images" above. Events located within the cluster mask of a given cluster were grouped into the event group belonging to that cluster. The event group of a cluster and the associated event time stamps were used to reconstruct the sequence of dark times as contiguous frame times that did not contain an event. All dark times from a cluster were pooled to obtain a normalised cumulative histogram of dark times for the cluster. The resulting dark time histogram was fit with an exponential expression $1\text{-}\exp(-t/\tau_D)$ to estimate the dark time constant τ_D. The inverse of the dark time τ_D^{-1} was calculated for each cluster and stored as the qPAINT index q_i of the cluster.

Calibration of qPAINT data. To obtain estimates of RyR numbers N_R for each cluster, a calibration was performed for each DNA-PAINT series. This was achieved by calculating a histogram of cluster qPAINT indices for very small clusters as identified by a cluster area < 10,000 nm^2. Visually these corresponded to clusters containing one or few puncta. The calibration histogram generally exhibited a prominent peak at a small qPAINT index value q_{i1} and secondary peaks at multiples of this value. The calibration value was determined by a multi-

exponential histogram fit with peaks at multiples of the fitting parameter q_{i1}. qPAINT indices of all clusters were combined with the calibration value q_{i1} to obtain absolute RyR number estimates N_R as the ratio q_i / q_{i1}.

The custom code that implements the calibrated qPAINT analysis is available as part of a supplementary code package compatible with recent versions of PyME. The code package was developed by the Soeller laboratory and can be downloaded at http://bitbucket.org/christian_soeller/pyme-extra.

Simulation of synthetic data

We used an algorithm implemented in the PyME software to generate synthetic single molecule localisation data as performed previously (13). As a first step, model images of punctate RyR labelling densities placed in either (a) a gridded organisation at a fixed spacing (supplementary Fig S2A) or (b) randomly placed and at a variable spacing to the next nearest neighbour as described by a random sample from a normal distribution with a specified σ. The centroids of the puncta were then convolved with a 2D Gaussian model with a σ of 5 nm (i.e. with a full width at half maximum of 12 nm) to smooth the mask. The model was used as a starting template in the PyME software which generated single molecule events within the labelled regions to match the imaging parameters (length of acquisition, event rates, localisation error) observed in the dSTORM and DNA-PAINT experimental data for simulation of dSTORM and DNA-PAINT imaging, respectively. The parameters used for the synthetic data simulation are summarised in Table S2. The generated point data were rendered into 2D greyscale data and subjected to the centroid computation algorithm identical to the experimental analysis protocol described above to closely mimic the processing of the experimental data.

Supplementary References

1. Jayasinghe ID, Cannell MB, & Soeller C (2009) Organization of ryanodine receptors, transverse tubules, and sodium-calcium exchanger in rat myocytes. *Biophysical journal* 97(10):2664-2673.
2. Jungmann R*, et al.* (2014) Multiplexed 3D cellular super-resolution imaging with DNA-PAINT and Exchange-PAINT. *Nature methods* 11(3):313-318.
3. Huang F*, et al.* (2016) Ultra-High Resolution 3D Imaging of Whole Cells. *Cell* 166(4):1028-1040.
4. Soeller C, Crossman D, Gilbert R, & Cannell MB (2007) Analysis of ryanodine receptor clusters in rat and human cardiac myocytes. *Proceedings of the National Academy of Sciences of the United States of America* 104(38):14958-14963.
5. Baddeley D*, et al.* (2009) Optical single-channel resolution imaging of the ryanodine receptor distribution in rat cardiac myocytes. *Proceedings of the National Academy of Sciences of the United States of America* 106(52):22275-22280.
6. Hou Y, Jayasinghe I, Crossman DJ, Baddeley D, & Soeller C (2015) Nanoscale analysis of ryanodine receptor clusters in dyadic couplings of rat cardiac myocytes. *Journal of molecular and cellular cardiology* 80:45-55.
7. Van Oort RJ*, et al.* (2011) Disrupted junctional membrane complexes and hyperactive ryanodine receptors after acute junctophilin knockdown in mice. *Circulation* 123(9):979-988.
8. Tokunaga M, Imamoto N, & Sakata-Sogawa K (2008) Highly inclined thin illumination enables clear single-molecule imaging in cells. *Nature methods* 5(2):159-161.
9. McGorty R, Kamiyama D, & Huang B (2013) Active microscope stabilization in three dimensions using image correlation. *Optical Nanoscopy* 2(1):1-7.
10. Lin R, Clowsley AH, Jayasinghe ID, Baddeley D, & Soeller C (2017) Algorithmic corrections for localization microscopy with sCMOS cameras - characterisation of a computationally efficient localization approach. *Optics Express* 25(10):11701.
11. Baddeley D*, et al.* (2011) 4D super-resolution microscopy with conventional fluorophores and single wavelength excitation in optically thick cells and tissues. *PloS one* 6(5):e20645.
12. Baddeley D, Cannell MB, & Soeller C (2010) Visualization of localization microscopy data. *Microsc Microanal* 16(1):64-72.
13. Hou Y*, et al.* (2014) Super-resolution fluorescence imaging to study cardiac biophysics: alpha-actinin distribution and Z-disk topologies in optically thick cardiac tissue slices. *Prog Biophys Mol Biol* 115(2-3):328-339.
