# Supplementary material for: True Molecular Scale Visualization of Variable Clustering Properties of Ryanodine Receptors
Source: Cell Rep. 2018 Jan 9;22(2):557–67. doi: 10.1016/j.celrep.2017.12.045 (PMC5775502; doi:10.1016/j.celrep.2017.12.045)
Supplement: Document S2. Article plus Supplemental Information [file mmc2.pdf]

Resource

Cell Reports

True Molecular Scale Visualization of Variable Clustering Properties of Ryanodine Receptors

Graphical Abstract

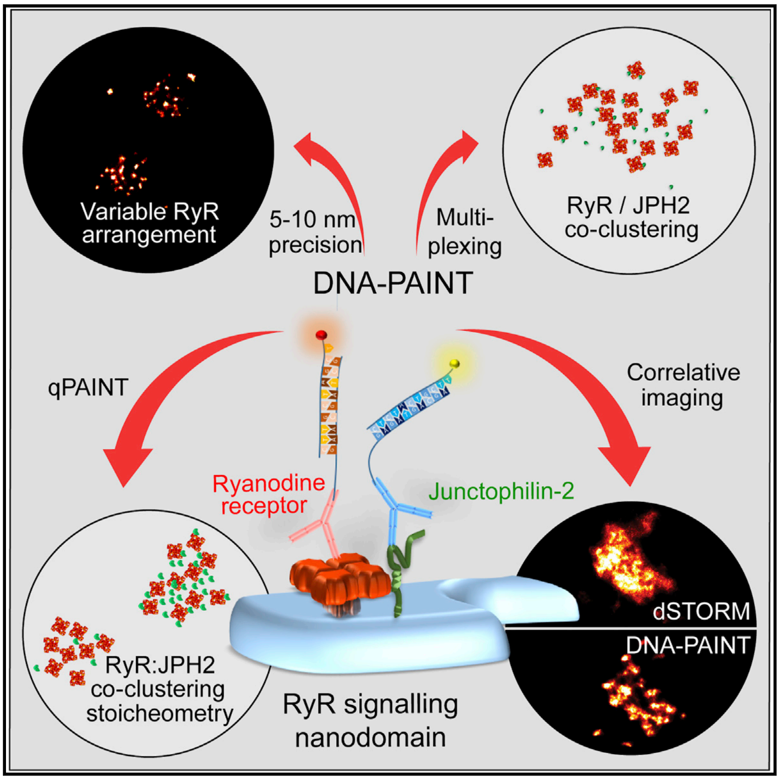

Authors

Izzy Jayasinghe, Alexander H. Clowsley, Ruisheng Lin, ..., David Baddeley, Lorenzo Di Michele, Christian Soeller

Correspondence

c.soeller@exeter.ac.uk

In Brief

Jayasinghe et al. resolve the distribution of single ryanodine receptors (RyRs) within intracellular signaling domains in cardiac myocytes with DNA-PAINT, a super-resolution microscopy approach. Individual RyRs are resolved within irregular cluster arrays. Quantitative imaging reveals significant variation in the co-clustering stoichiometry between RyRs and the regulatory protein junctophilin-2.

Highlights

- With enhanced resolution, individual RyRs were observed within dense clusters
- RyRs appear to assemble randomly and unconstrained
- The ratio of RyR co-clustering with JPH2 varies considerably between nanodomains
- DNA-based super-resolution enables fully quantitative imaging at ∼10 nm resolution

Jayasinghe et al., 2018, Cell Reports *22*, 557–567
January 9, 2018 © 2017 The Author(s).
https://doi.org/10.1016/j.celrep.2017.12.045

Cell Reports
Resource

OPEN ACCESS
CellPress

True Molecular Scale Visualization of Variable Clustering Properties of Ryanodine Receptors

Izzy Jayasinghe,[1,2,5] Alexander H. Clowsley,[2,5] Ruisheng Lin,[2] Tobias Lutz,[2] Carl Harrison,[2] Ellen Green,[2] David Baddeley,[3] Lorenzo Di Michele,[4] and Christian Soeller[2,6,*]
[1]Faculty of Biological Sciences, University of Leeds, Leeds, UK
[2]Living Systems Institute, University of Exeter, Exeter, UK
[3]Auckland Bioengineering Institute, University of Auckland, Auckland, New Zealand
[4]Cavendish Laboratory, University of Cambridge, Cambridge, UK
[5]These authors contributed equally
[6]Lead Contact
*Correspondence: c.soeller@exeter.ac.uk
https://doi.org/10.1016/j.celrep.2017.12.045

SUMMARY

Signaling nanodomains rely on spatial organization of proteins to allow controlled intracellular signaling. Examples include calcium release sites of cardiomyocytes where ryanodine receptors (RyRs) are clustered with their molecular partners. Localization microscopy has been crucial to visualizing these nanodomains but has been limited by brightness of markers, restricting the resolution and quantification of individual proteins clustered within. Harnessing the remarkable localization precision of DNA-PAINT (<10 nm), we visualized punctate labeling within these nanodomains, confirmed as single RyRs. RyR positions within sub-plasmalemmal nanodomains revealed how they are organized randomly into irregular clustering patterns leaving significant gaps occupied by accessory or regulatory proteins. RyR-inhibiting protein junctophilin-2 appeared highly concentrated adjacent to RyR channels. Analyzing these molecular maps showed significant variations in the co-clustering stoichiometry between junctophilin-2 and RyR, even between nearby nanodomains. This constitutes an additional level of complexity in RyR arrangement and regulation of calcium signaling, intrinsically built into the nanodomains.

INTRODUCTION

The advent of single-molecule switching and localization-based PALM, STORM, and related super-resolution microscopies (Betzig et al., 2006; Hess et al., 2006; Rust et al., 2006) has greatly advanced insight in cell biology over the last decade. Significant breakthroughs in visualizing nanostructures within cells include optically resolved nuclear pore complexes (Szymborska et al., 2013), microtubules (Mikhaylova et al., 2015), actin-spectrin scaffolds for membranes (Xu et al., 2013), membrane compartments (Shim et al., 2012), and protein ensembles in signaling nanodomains (Gambin et al., 2013). This includes the visualization of the clusters of the giant (~2 MDa) ryanodine receptor-2 (RyR) Ca^{2+} release channels in cardiomyocytes to characterize the calcium signaling nanodomains, which are the structural units of calcium signaling in cardiac myocytes (Baddeley et al., 2009) using dSTORM (Heilemann et al., 2008).

The cardiac ryanodine receptor, RyR2, is strongly expressed in the heart and brain and provides the molecular basis of a mechanism known as calcium-induced calcium release (CICR), in which RyRs are transiently opened via calcium from adjacent calcium channels (Fabiato, 1985; Franzini-Armstrong and Protasi, 1997). Due to their vital role in physiology and pathology RyRs present a key target for molecular investigations. At the supra-molecular level the clustering of RyRs is of major interest, both because it can dramatically modulate the excitability of RyRs (Walker et al., 2014) and due to their general role in calcium signaling in muscle (Allen et al., 2008; Cannell and Kong, 2012), neurons (Manita and Ross, 2009), and secretory cells like pancreatic beta cells.

RyR clusters are a prototypical system for which the biophysical cluster properties, e.g., cluster excitability, can be directly tied to the spatial cluster arrangement of the RyRs as recently shown (Walker et al., 2014). The regulation of RyR channels is under the acute control of both cytoplasmic and internal store [Ca^{2+}] but is also a product of the local configuration of other molecular components. These include accessory proteins such as junctophilin-2 (JPH2), which are anchored in the intracellular store membrane. Local JPH2 can directly modulate the Ca^{2+} released by RyRs as well as adjusting the physical size of RyR clusters (Munro et al., 2016).

In previous work, application of dSTORM (or stimulated emission depletion [STED]) to study the clustering properties of RyRs within cardiomyocytes achieved effective lateral spatial resolution of 40–60 nm, with quantitative analysis based on the assumption that RyRs are arranged on a regular 30 × 30-nm quasi-crystalline lattice (Baddeley et al., 2009). Electron tomography data have recently challenged this assumption and suggested that the arrangement may be more variable (Asghari et al., 2014); due to the complexities of electron tomography, this has not been replicated in other laboratories to date. To resolve such open questions there is a growing need for

This is an open access article under the CC BY license (http://creativecommons.org/licenses/by/4.0/).

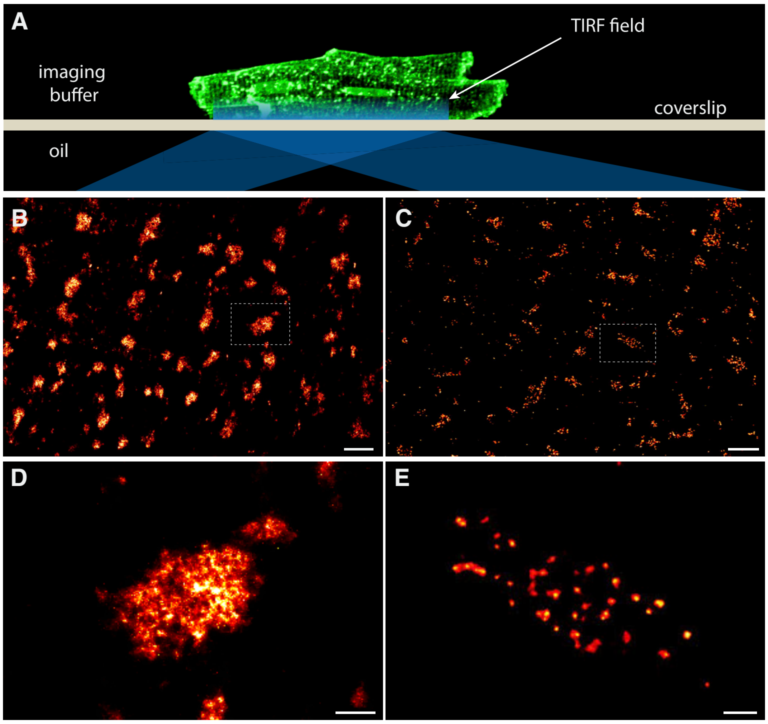

Figure 1. Visualization of RyRs in Peripheral Couplons of Ventricular Myocytes

(A) TIRF illumination of peripheral RyR labels adjacent to the coverslip.

(B and C) Both dSTORM (B) and DNA-PAINT (C) typically show RyR in clustered nanodomains (which are loosely in a transverse striated pattern) facing the surface plasmalemma (parallel to image plane).

(D and E) Magnified views of clusters from these images (boxed regions in B and C) show the unresolved cluster substructure in dSTORM data (D), which contrasts with resolved punctate RyR patterns mapped with DNA-PAINT (E).

Scale bars in (B) and (C), 1 μm, and in (D) and (E), 100 nm.

quantitative super-resolution methods, which can consistently and robustly achieve higher resolution in complex cells.

A recent microscopy approach known as DNA-PAINT utilizes the specificity and predictability of DNA hybridization to localize molecular targets tagged with synthetic single-stranded DNA oligonucleotides (Jungmann et al., 2014). When compared with more conventional localization microscopy approaches using switchable labels DNA-PAINT has two important advantages. It allows the use of extremely bright and stable dyes along with buffer conditions that optimize photon yield (rather than switching performance) leading to significantly improved localization precision. In addition, it is compatible with high accuracy target quantification given suitable calibration (Jungmann et al., 2016).

In this study, we achieve true molecular resolution of RyRs within tightly organized junctional nanodomains, enabled by DNA-PAINT, to understand the *in situ* molecular interactions of RyR and the related junctional molecule JPH2. We developed a nanoscale Monte Carlo simulation of protein cluster assembly, to test hypotheses on mechanisms of protein clustering, and uncertainties in protein labeling. The observed RyR patterns within clusters are consistent with a random and unconstrained cluster assembly process, unlike the crystalline lattices seen in *in vitro* assays and in skeletal muscle cells. Quantitative co-localization analysis of RyRs and JPH2 revealed a fraction of JPH2 within 50 nm of RyRs, consisted with RyR-JPH2 complex formation. Using a qPAINT target counting algorithm, we made the striking observation that the ratio of RyRs co-clustering with its inhibitory partner protein JPH2 can vary manifold. Overall, the data reveal that RyR nanodomains exhibit localized structural variations compatible with extremely different excitability and calcium release properties.

RESULTS

Improved Visualization of RyRs within Nanodomains

To visualize the immuno-labeling of RyRs in peripheral clusters (or "couplons") of rat ventricular myocytes, cells adhered to coverslips were studied in total internal reflection fluorescence (TIRF) mode (Figures 1A and S1). dSTORM images of surface membrane areas attached to the coverslip revealed clusters of RyR labeling loosely organized in a transversely striated pattern (Figure 1B), as seen before (Baddeley et al., 2009). DNA-PAINT imaging of RyR in similar cells was performed in very similar imaging settings allowing single-marker detection as the "imager" oligonucleotides bind to their complementary "docking" strands within the TIRF field (Figures S1A and S1B). Grayscale rendering of the imager localizations (Figure 1C) produced a labeling pattern, which broadly reflects the periodic arrangement of sarcomeres with most RyRs close to the z-lines (Baddeley et al., 2009), similar to the distribution seen with dSTORM.

At closer inspection, however, the DNA-PAINT images reveal very distinctive puncta within the cluster area unlike in the dSTORM images (Figures 1D and 1E). Even in the primary event localization position maps (Figures S1C and S1D), this pattern was clearly discernible. Examination of the reason for this improved resolution of RyR markers revealed a higher photon yield in DNA-PAINT events (Figures S1E), which contributed to an ~6-fold improvement in localization error (Figure S1F).

Reproducibility of RyR Localization with DNA-PAINT

We hypothesized that these puncta reveal individual RyRs within the essentially flat 2D peripheral clusters (Franzini-Armstrong et al., 1999), which dSTORM data could not resolve. We conducted a series of experiments to reconcile this morphology with the previously observed dSTORM images of the same structures as well as experiments to confirm that the morphology observed in the DNA-PAINT data is robust.

Similar results were obtained with directly labeled primary antibodies against RyRs as with the secondary labeling system (Figure S3), due to the relatively large size of RyRs (~30 nm); we therefore adopted the secondary system for most experiments.

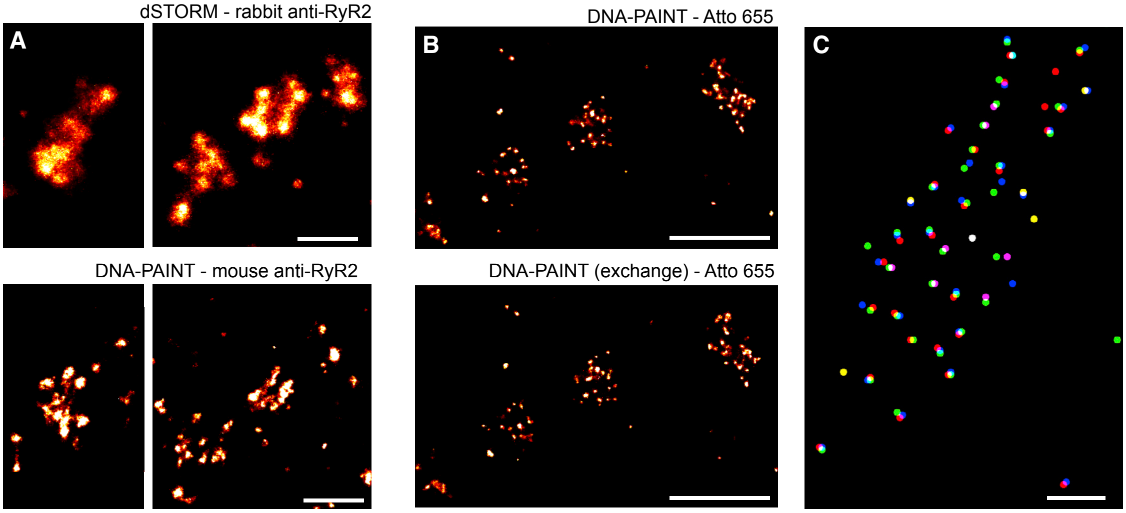

Figure 2. Reproducibility of RyR Cluster Nanostructure with DNA-PAINT Imaging

(A) dSTORM images of RyR clusters (upper) in peripheral nanodomains showed clear visual agreement with correlative DNA-PAINT images of the same clusters (lower).

(B) Sequential exchange-PAINT images acquired of RyR clusters illustrate the high reproducibility of both cluster shapes and punctate morphology.

(C) Overlay of the centroids of each RyR punctum in three independently acquired DNA-PAINT images (red, green, and blue) of the same cell confirms this. Scale bar in (A), 250 nm, in (B), 500 nm, and in (C), 250 nm.

We also assessed the consistency between DNA-PAINT reported RyR clusters and dSTORM cluster data. A correlative imaging experiment where DNA-PAINT (using ATTO 655 imagers) and dSTORM (using Alexa Fluor 647 secondary antibodies) were performed using a mouse monoclonal anti-RyR2 and a rabbit polyclonal anti-RyR2 (previously shown with dSTORM to report >70% spatial agreement [Hou et al., 2015]), respectively. The correlative dSTORM and DNA-PAINT images (Figure 2A) revealed strong spatial agreement of the cluster positions and the shapes. They also confirmed that the punctate RyR labeling in DNA-PAINT images were essentially confined to the cluster area resolved with dSTORM.

DNA-PAINT has "photo-bleaching free" properties due to unlimited replenishment of imagers. We exploited this for sequentially acquiring correlative DNA-PAINT images of the same RyR clusters. This was achieved by washing imager strands matching the RyR secondary antibodies repeatedly in and out again as done in exchange-PAINT (Jungmann et al., 2014) (Figure 2B). These experiments established that the locations and the morphology of the puncta were robustly reproduced with a median error (displacement of a given punctum in a pairwise comparison of images) of 0.94 nm (47 clusters; 5 cells). 94.2% ± 0.9% (mean ± SEM, 5 cells) of puncta locations were reproduced within 10 nm (80.9% within a stringency of 5 nm) across three sets of exchange-PAINT images sets of DNA-PAINT images in the correlated areas (47 clusters; 5 cells). An example of this reproducibility is illustrated in Figure 2C where localized and detected puncta positions from three independent exchange-PAINT repetitions (red, blue, and green dots) confirm strong alignment and reproducibility. These results show that the puncta are a robust feature of the DNA-PAINT RyR data.

Given the localization precision (5–10 nm), individual puncta represent locations of single RyRs as it is unlikely that markers bound to the same RyR can be resolved into distinguishable puncta. As a consistency check, we performed a simulation that confirmed that a regular grid of RyRs spaced as close as 30 nm can be resolved and detected at event densities similar to those shown in our data (e.g., Figure S2A). This is also broadly consistent with imaging of a test sample made from DNA origami on our system that clearly resolved 40-nm distant puncta using localization data with a similar precision as in our RyR data (Figures S3C and S3D).

The above tests hence confirmed that marker positions are robustly reproduced over several DNA-PAINT imaging repetitions and puncta occurred at a spacing compatible with detection based on the high localization precision data.

RyR Clustering and Quantitative Analysis by *In Situ* Calibrated qPAINT

The reproducibility and the static positions of the punctate event densities observed in DNA-PAINT data are strong evidence that these reflect the positions of RyRs within the underlying cluster. The resolved RyR positions were useful in investigating clustering patterns and spatial densities of RyR arrangement, which critically determine their cross signaling via calcium (Walker et al., 2014). RyR numbers detected as in Figures S1D within 2,062 clusters (17 cells) segmented clusters (red outlines in Figure 3A), revealing a frequency histogram of cluster size with an approximately exponential distribution (Figure 3B). This distribution, in shape, was similar to that constructed previously with dSTORM data (Baddeley et al., 2009); however, with a smaller average size of 8.8 ± 0.86 (n = 17 cells) RyRs per cluster. To analyze clustering pattern, we measured the nearest neighbor center-to-center distance (NND) for each RyR punctum in clusters with $\geq$2 RyRs. From 21 cells analyzed, a near-Gaussian distribution with a mean of 40.1 ± 0.9 nm (Figure 3B inset; red) was

OPEN
ACCESS
CellPress

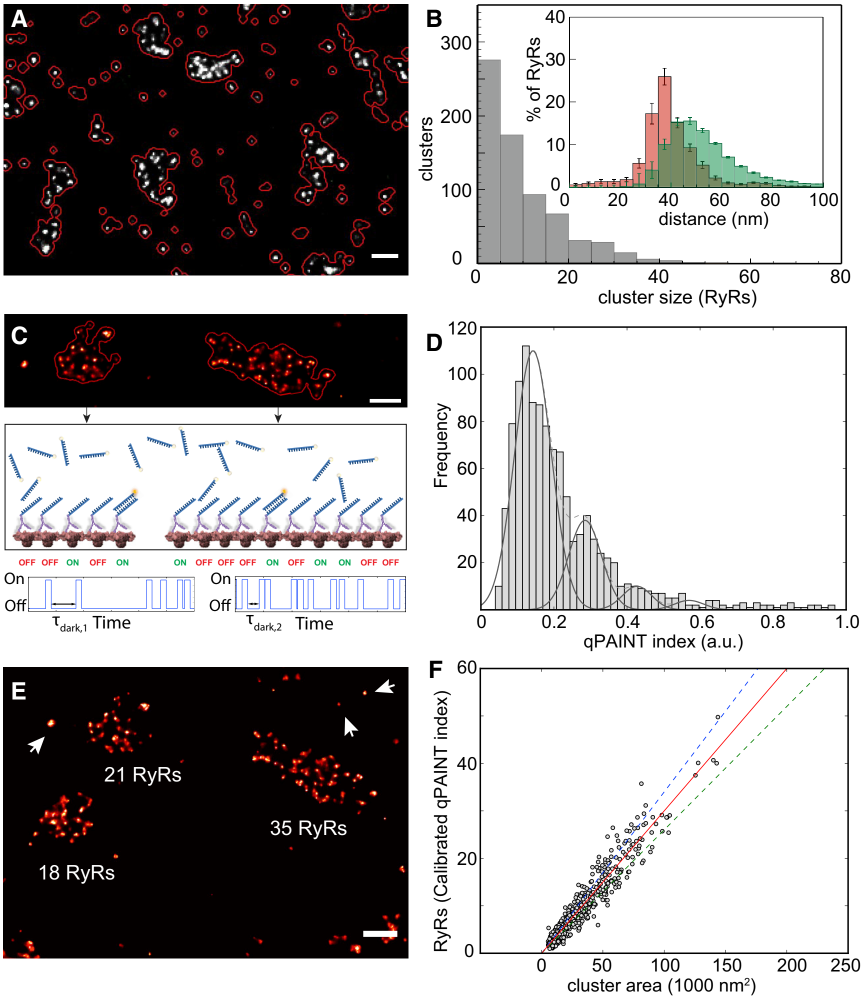

Figure 3. Quantitative Analysis of RyR Cluster Properties

(A) Clusters were segmented using an algorithm that contours the image based on local event density (red lines).

(B) A frequency histogram of RyR puncta counted within segmented peripheral RyR clusters typically consisted of fewer than 40 RyRs (mean ± SD = 8.81 ± 3.56 RyR/cluster; n = 17 cells, 10 animals); the distribution of nearest neighbor puncta centroid distances within clusters with 5 or more RyRs (see inset; red) showed a mean of 40.1 ± 0.9 nm (mean ± SEM; n = 1,802 clusters, 8 animals). The average of the 4 nearest neighbor distances (green) was a right-shifted distribution (58.9 ± 0.9 nm). Error bars (inset): SEM for n = 17 cells.

(C) qPAINT estimates the number of docking sites by analyzing the temporal sequence of single-molecule event detections recorded at a given cluster. Two clusters are shown (upper panel), one larger than the other. The rate at which imagers bind to RyRs in a cluster is proportional to the number of RyRs in the cluster (middle and lower panels).

(D) The "qPAINT indices" of small clusters containing ~1 punctum provided a calibration to "count RyRs"; its histogram exhibits equidistant peaks characteristic of quantal increments in imager strand binding sites with a primary maximum of 0.14 qPAINT index units, equivalent to a single RyR.

(E) Examples of the RyR counts estimated from calibrating the qPAINT index values for three nearby large clusters show the close correspondence with counted puncta numbers.

(F) Scatterplot of cluster area versus qPAINT estimated RyR counts. A linear regression (solid line) of the scattergram provides an estimate of the two-dimensional area of packing as 1 RyR per 3,300 nm^2 for this dataset from one cell (391 clusters). Dashed lines indicate 15% variation of slope. Scale bars in (A) and (E), 200 nm.

observed. In a nanodomain with variable RyR spacing, typified by the DNA-PAINT images, the average of the distances to each of the surrounding neighbors would describe the typical calcium diffusion distances relevant to RyR-RyR communication. The average 4-neighbor distance (4ND) for these data (for clusters with $\geq$5 RyRs) had a mean of 58.9 ± 0.9 nm and a mode at ~47 nm (Figure 3B inset; green).

We subjected the same RyR data to an algorithm called qPAINT, which quantifies protein numbers from DNA-PAINT data (Jungmann et al., 2014), as a second, largely independent analysis from the puncta counting approach. This method utilizes the average "off" times absent of an imager binding event within a cluster or nanodomain area (schematically shown in Figure 3C), under fixed imaging conditions. This off-time is proportional to the number of docking sites, i.e., the number of markers (see Figure S4 and Supplemental Experimental Procedures). We conducted the qPAINT analysis on a per-cluster basis (e.g., Figure 3A) to plot cumulative histograms of the dark time durations and obtain the average dark time between binding events unique to each cluster (e.g., Figure S4D). Due to the first-order binding kinetics between imager strands and docking strands, the inverse of the measured dark time, which we term the "qPAINT index," provides a measure directly proportional to the number of docking strands in the cluster region (for details, see Experimental Procedures). Figure 3D shows a histogram of the qPAINT index of small clusters that were selected based on their measured geometrical area and visually contain ~1 punctum when rendered (Figures S4E and S4F). The qPAINT index histogram of these small clusters exhibits a number of peaks that are a hallmark of "quantal" behavior representing one, two, three, etc., units characteristic of single RyRs. The mean qPAINT index for single RyR obtained in this way, e.g., 0.14 in Figure 3D, was used to calibrate the cluster qPAINT indices in the corresponding image and turn them into absolute RyR number estimates. Figure 3E illustrates RyR number estimates for 3 larger clusters. The estimated RyR numbers closely agree with the counted number of puncta in these clusters as shown in Figure S4G, further supporting our hypothesis that individual puncta correspond to individual RyRs. When the estimated RyR numbers of each cluster are plotted against the geometrical area of clusters (Figure 3F), a linear relationship was obtained that was used to estimate the apparent RyR density within clusters. The slope of

OPEN
ACCESS
CellPress

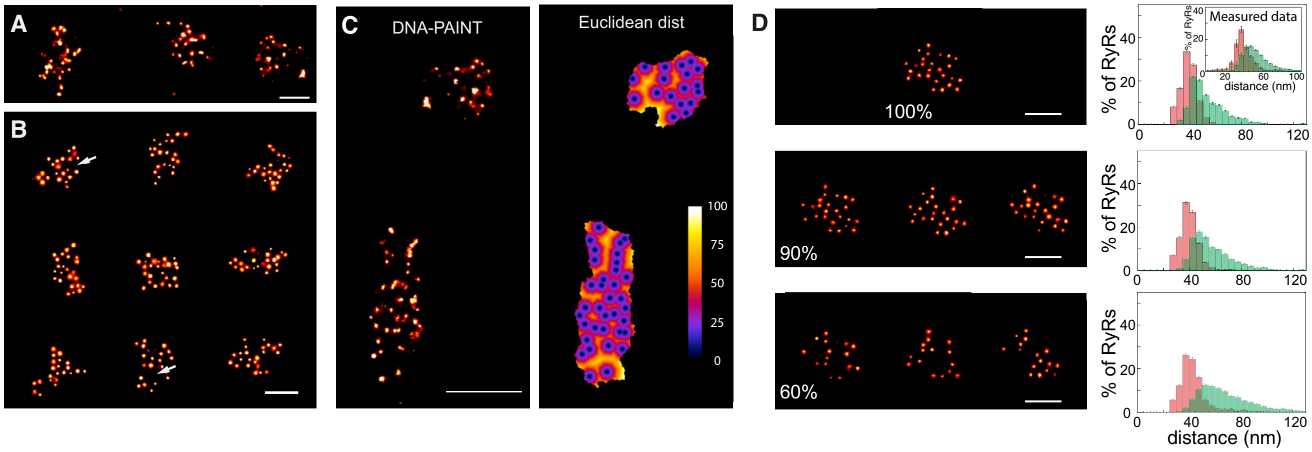

Figure 4. Morphology of RyR Organization within Clusters

(A) DNA-PAINT example of three adjacent peripheral RyR clusters with their typical irregular cluster shapes and puncta arrangement.

(B) Simulated super-resolution images with similar morphology were reproduced in an *in silico* Monte Carlo simulation of directionally unconstrained RyR cluster self-assembly. Note the gaps (arrows) within the arrays that naturally appeared within the cluster due to the random self-assembly process.

(C) Experimental data (left) were further analyzed for the Euclidean distances (color maps in nm) between the centroids of RyR labeling densities. Regions colored in yellow-white were sub-nanodomain boundaries within the RyR cluster where the gaps exceeded 100 nm.

(D) Simulation of effect of binding analysis by reducing the detection fraction from top to bottom panel 100%, 90%, and 60% (examples of iterations on left) revealed minor changes to the observed nearest-neighbor (red) and average-4-neighbor distance (green) histograms. Experimentally measured distance distributions are shown in inset. Error bars: SEM for n = clusters; (inset) SEM for n = 17 cells.

Scale bars in (A) and (B), 200 nm, and (C), 400 nm, (D), 150 nm.

this relationship was 0.32 ± 0.05 RyR / 1,000 nm^2, (mean ± SEM, 1,507 clusters, 6 cells from 3 animals) equivalent to a mean linear distance between RyRs of ~56 nm, similar to the distance obtained above as the 4ND (~59 nm, see above).

This quantitative analysis concludes that in the high-quality DNA-PAINT data we can see markers identifying the location of individual RyRs. It also confirms that RyRs occur at a density within clusters that is lower than that expected in a close packed arrangement of RyRs with a limiting density of ~1 RyR / 1,000 nm^2, as seen *in vitro* (Yin and Lai, 2000).

Analysis of the Organization of RyRs within Clusters

The clustering pattern of cardiac RyRs within their nanodomains is thought to be an important aspect determining the "excitability" of the nanodomain as a whole, and as the diffusion of Ca^{2+} between RyRs, which steeply depends on distance from open RyRs. The puncta positions analyzed above suggest that the spacings between RyRs are not uniform. This pattern is visually observable in DNA-PAINT data (Figure 3A). We sought to simulate clustering patterns that best captured these patterns. Experimental clusters could be closely mimicked by assembling simulated clusters according to a rule that constructed clusters by placing new RyRs in a random direction and with a distance that varied slightly around a mean distance of 40 nm according to a Gaussian distribution with a sigma of 7.4 nm (Figure 4B), a distribution that closely matches the observed distance distribution in mean and width (see also Figure 3B). This self-assembly process led to the appearance of some larger "gaps" in the clusters similar to those observed in our data as highlighted in distance maps between RyRs in experimentally observed clusters as shown in Figure 4C.

This simulation allowed us to investigate additional considerations in analyzing RyR patterns with a method such as DNA-PAINT. For example, the binding of antibodies to RyRs may be incomplete, which could affect the interpretation of the data. We sought to put bounds on these effects to inform data interpretation. Comparison of simulations based on clusters generated with the random direction cluster model and assuming detection levels of 100%, 90%, and 60% are shown in Figure 4D (extended in Figure S2B). Increasingly lower detection fractions shift the average of local distances to longer values and especially give rise to a marked tail in the 4ND distribution. By contrast, the NND stays approximately constant. Comparison suggests that our observations are consistent with a model where we detect >80% of all RyRs as otherwise gaps and 4NDs would markedly increase. The simulated clusters, the shapes, and modes of their histograms also confirmed that the 40-nm RyR spacing we observed in the experimental data (Figure 3B inset) could not be the result of lower detection efficiency (50%) of more closely spaced RyRs (30 nm). See Figure S2C.

Investigation of RyR-JPH Co-clusters with Exchange-PAINT

The apparent irregular organization of RyRs within clusters prompted us to investigate how this nanostructure was compatible with co-clustering with JPH2, a regulator of RyR opening as well as a key molecular tether of junctions. Applying exchange-PAINT (i.e., sequential imaging of two populations of docking-strand markers) (Jungmann et al., 2014) revealed JPH2 within the same nanodomains as RyR clusters (Figure 5A, left), in co-clustered regions of JPH2 labeling (Figure 5A, mid). Overlay of the two images confirmed intimate tessellation of both RyR

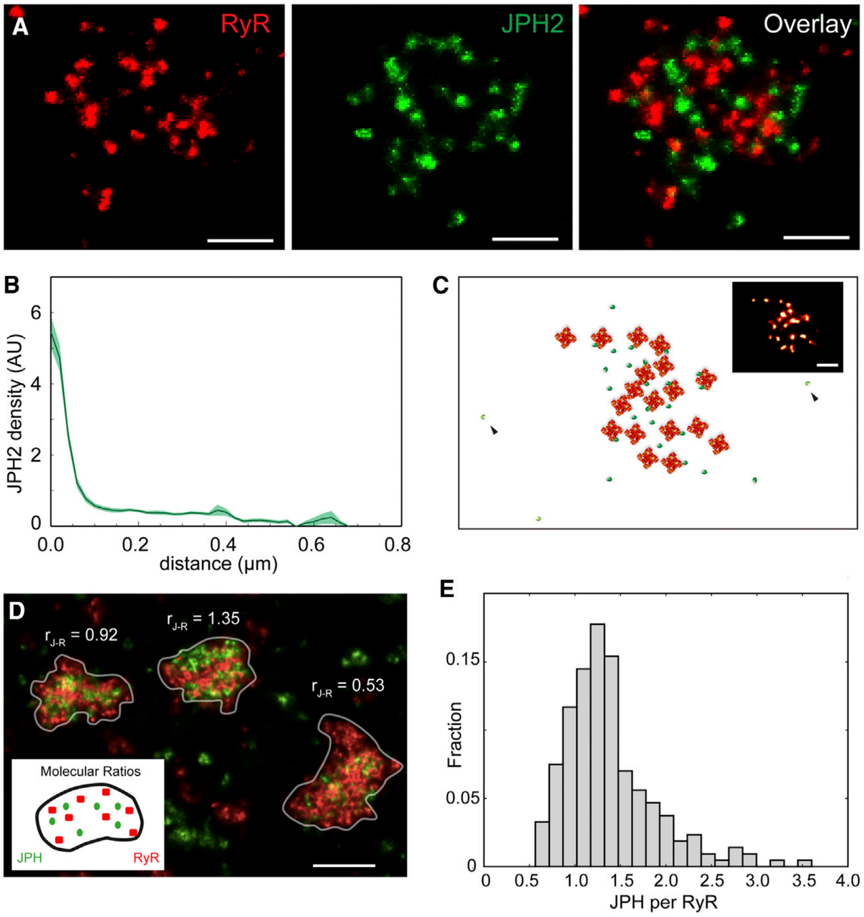

Figure 5. Exchange-PAINT of JPH2 Interaction with RyR in Peripheral Clusters

(A) Example exchange-PAINT images of RyR (red), JPH2 (green), and their overlay (right).

(B) Analysis of the JPH2 labeling density as a function of the distance from the centroids of RyR puncta resolved a high density of JPH2 markers within 50 nm of the RyRs. Error bars: SEM for n = 8 cells.

(C) A schematic reconstruction of protein arrangements compatible with the DNA-PAINT data (inset): an irregular RyR array (orange) and populations of densely co-localized or bound JPH2 (dark green) and sparsely distributed JPH2 (light green).

(D) Molecular ratios by exchange qPAINT of RyR and JPH2 determined from calibrated qPAINT data of RyR-JPH2 co-clusters (inset shows schematic), shown in three adjacent clusters exhibiting ratios between 0.53 and 1.35 JPH2/RyR.

(E) Histogram illustrating the distribution of JPH2 to RyR ratios (mean 1.38, mode at 1.25, and a width of 0.5 (SD), n = 250 clusters (containing $\geq$15 RyRs), 3 cells, 2 animals).

Scale bar in (A), 200 nm, (C), 100 nm, and (D) 250 nm.

and JPH2 labeling densities (Figure 5A, right). Analysis of a large ensemble of clusters exhibited a high density of JPH2 labeling within just 50 nm of the centroids of RyR puncta (Figure 5B). By superimposing the RyR data with simulated DNA-PAINT images that contained a random spatially uniform distribution of JPH2 labeling, we confirmed that this increased density of JPH2 in the immediate regions adjacent to RyRs reflects a preferential (non-random) co-clustering behavior (Figure S5A). Visual comparisons (Figure S5B) were striking how this pattern of molecular arrangement was not apparent from dSTORM data. When investigated within cluster boundaries, as expected, dSTORM and exchange PAINT data reported arrangements between RyR and JPH2 that were quantitatively consistent. For example, the mean percentage of JPH2 overlapping within the area of the RyR cluster was similar between dSTORM and DNA-PAINT images (72.0% versus 70.3%; df = 11; $p = 0.49$ in Student's t test).

Further to the high-density fraction of JPH2s close to RyRs (<50 nm), a non-zero JPH2 density was present at distances of >50 nm in Figure 5B. Examining the DNA-PAINT data with a Euclidean distance analysis revealed that this sparse subset of JPH2 occupied some of the gaps in the RyR arrays illustrated in Figure 4C (see Figure S5C for analysis). Taken together, these observations are highly suggestive of a sub-population of JPH2 molecules located within RyR clusters that are unlikely to be directly bound to RyRs (labeling at distances >50 nm), as shown schematically in Figure 5C, while another sub-population is close enough to be conceivably in a molecular complex with individual RyRs.

The high degree of co-clustering motivated an additional analysis that was enabled by the quantitative nature of qPAINT. By combining qPAINT analysis with the exchange-PAINT of RyR and JPH2, we determined molecular ratios of JPH2 to RyRs on a per cluster basis. The analysis was conducted for larger RyR clusters (>15 RyRs) that are likely located in larger junctions between surface and SR membranes. Figure 5D shows three typical clusters with ratios $r_{J\text{-}R}$ of JPH2 to RyR between $r_{J\text{-}R} = 0.53$ and $r_{J\text{-}R} = 1.35$. It is notable that clusters with considerably different molecular ratios are observed in close proximity. An ensemble analysis (214 large clusters, n = 3 cells) revealed a distribution in which ratios varied between ~0.5 and 3.5 with a mean at 1.38 JPH2 per RyR, a mode at 1.25 and a histogram width of 0.5 (quantified by the SD) as shown in Figure 5E. The molecular ratio determined *in situ* using qPAINT analysis, presents a measurement that is spatially more sensitive, particularly for analysis of nanodomains, than bulk (*in vitro*) biochemical analysis (e.g., immuno-co-precipitation).

DISCUSSION

The use of DNA-PAINT-based super-resolution imaging enabled fully quantitative imaging of clusters of RyRs in peripheral couplings of ventricular myocytes with molecular resolution.

Table 1. RyR Parameter Properties Estimated from DNA-PAINT Data

RyR cluster size[a,b]	8.81 ± 3.56 (n = 17 cells, 10 animals)
Nearest neighbor distance[a,c]	40.1 ± 0.9 nm (n = 1,802 clusters, 8 animals)
Average of distances to 4 nearest neighbors[a,c]	58.9 ± 0.9 nm (n = 1,802 clusters, 8 animals)
RyR packing density within cluster[a,d]	3,300 nm^{-2} (n = 391 clusters)
JPH2/RyR ratio[e,f]	1.38 ± 0.5 (n = 250 clusters, 2 animals)

[a]All measurements shown as mean ± SEM.
[b]Estimated from counting punctate densities of all RyR-labeled regions in TIRF DNA-PAINT images.
[c]From clusters containing ≥5 RyR.
[d]From the slope of the linear slope in scatterplot of cluster area versus qPAINT estimated RyR counts.
[e]Shown as mean ± SD of histogram.
[f]From qPAINT analysis of RyR and JPH2 exchange-PAINT data.

Improved localization precisions were critical in resolving individual RyRs and revealing clustering patterns at an unprecedented level of spatial detail. The data revealed apparently irregular arrangements of RyRs with considerable gaps within clusters and an apparent RyR density lower than expected for close-packed RyR arrays. The improved resolution showed that a sub-population of JPH2 was in molecular proximity to RyRs, compatible with being in a complex with individual RyRs, while per-cluster ratios of JPH2 to RyRs are highly variable.

Clusters of RyRs at True Molecular Resolution

The data presented here show individually resolved RyRs and their arrangements into clusters using purely optical methods. Previous super-resolution imaging using dSTORM (Baddeley et al., 2009) and STED (Walker et al., 2014) had been able to resolve cluster outlines, but the lower resolution (~50–60 nm full width at half maximum [FWHM]) prohibited determination of the density and arrangement of RyRs within individual clusters. Using DNA-PAINT, distinct puncta were observed that appear to arise from markers bound to individual RyRs. The puncta were highly reproducible in location and number; their observed distance is compatible with the expected ability of our DNA-PAINT imaging system to resolve objects ≤30 nm apart. With our labeling system and since RyRs as homo-tetramers provide several binding sites for antibodies (ABs), individual puncta may arise from more than one marker. A single RyR should not give rise to more than one punctum that can be spatially resolved since RyRs are less than 30 nm in diameter (Peng et al., 2016); if markers were bound in different places of a single RyR, these would be unlikely to be resolved into several puncta to give rise to the observed ≥40-nm neighbor spacings. The idea of each punctum representing a RyR is further supported by a second analysis that used the recent qPAINT analysis method (Jungmann et al., 2016). The qPAINT calibration measurements (Figure S4H) are compatible with a quantized distribution of groups of docking sites. The size of the quantal groups corresponded closely with individual puncta, compatible with a scenario where each punctum arises from markers complexed with a single RyR.

DNA-PAINT Achieves Molecular Resolution with Relatively Low Experimental Complexity

Our data confirm that DNA-PAINT can provide very high spatial localization, with comparatively low optical complexity and demands on dye photo-physics, so that we could achieve high precision routinely in relatively complex biological preparations. As shown recently (Dai et al., 2016; Jungmann et al., 2014), the photon yields for imaging with DNA-PAINT improve the localization precision to <10 nm, and provided drift can be compensated, which we achieved with a transmitted light tracking system.

We made some modifications to the labeling system as described by Jungmann et al. (2014), notably by adding dyes to the docking strands so that successful staining and determination of suitable imaging areas was as straightforward as with conventional immuno-fluorescence imaging. This increased throughput and success rates in our experimental processing pipeline.

Another important advantage of DNA-PAINT is the ability to quantify the number of binding sites and calibrate the number of RyRs. qPAINT as described by Jungmann et al. (2016) relies only on binding between complementary DNA strands rather than models of dye photo-physics, which can be a source of uncertainty. We adapted qPAINT as an independent counting approach for RyRs and, in combination with exchange-PAINT, extended it to determine protein ratios between RyRs and JPH2. The DNA-based technology makes the scheme very flexible and delivered a robust quantitative super-resolution approach. The quantitative ratio analysis performed here, benefiting from the resolution to accurately recognize discrete nanodomains, provides information well beyond the sensitivity of classical analyses such as co-immunoprecipitation.

RyR Cluster Properties

The key properties of RyR molecular expression as revealed by our data are summarized in Table 1. Both puncta counting and qPAINT analysis provided estimates of an RyR density within clusters of ~0.3 RyRs/1,000 nm^2. This is about 3 times lower than the dense packing in artificial lipid membranes where RyR density was ~1 per 31.5^2 nm^2 = 1 per 992 nm^2 (Yin and Lai, 2000). This is consistent with the apparent distance between RyR puncta in our data, which was >40 nm, rather than ~32 nm as expected for dense packing. While it is possible that some RyRs were not detected because no markers were bound, such an effect is unlikely to explain the differences. We have verified that the marker system can detect proteins at considerably higher density, as demonstrated with CAV3, which was detected at a >3-fold higher density (Figure S6). In addition, we carried out simulations that suggest that our detection efficiency is high (discussed below).

Consistent with the lower RyR density the average size of RyR clusters was smaller (~64%) than earlier estimates, although the reduction was not in direct proportion to the lower density. This could result from over-estimates of cluster areas due to lower resolution in the original dSTORM data (Baddeley et al., 2009).

RyR Cluster Morphology and RyR Biophysics

The distance between RyRs within clusters and the spatial arrangement of RyRs, the "cluster morphology," is thought to play a major role for the excitability of a cluster by calcium (Walker et al., 2014, 2015). While EM methods can provide data with sufficient resolution, limits of contrast and throughput have meant that only a small number of clusters could be studied in this way (e.g., Asghari et al., 2014). The data provided here allow for the investigation of many clusters and their detailed RyR arrangement. Our analysis exploited the feature that peripheral couplings run parallel to the surface membrane (Franzini-Armstrong et al., 1999), which is attached to the coverslip and therefore the clusters are essentially flat in 2D. By restricting TIRF excitation to <100 nm from the coverslip interface, we ensured that deeper clusters with more complex orientations were excluded.

The distribution of puncta appeared irregular and could be mimicked using a model in which clusters were generated using placement of new RyRs in random directions and at a variable distance around a mean of ~40 nm. Indeed, puncta NNDs, important in determining the ability of an open RyR to open adjacent RyRs (Groff and Smith, 2008; Winslow et al., 2006), had a strong mode at ~40 nm, larger than the distance of ~32 nm expected for dense packing of RyRs. This is consistent with recent electron tomography data, which showed that RyRs in clusters are not homogeneously packed.

A complication in analyzing distances between RyRs could arise from marker jitter, since we localize dye molecules conjugated to antibodies rather than the RyRs themselves. This could lead to an offset between reported marker positions and actual RyR locations that may be between 0 and ~12.5 nm (Mikhaylova et al., 2015). Since >1 primary antibody may bind to a single RyR, the center of the resulting punctum may be quite close to the center of the RyR. As a result, the marker jitter may be small, but it is difficult to conclusively identify regularly packed subgroups of RyRs. Notably, directly labeled primary antibodies do not resolve the jitter as primary antibodies may bind to RyRs with a variable offset depending on the location of epitopes on the large ~27 × 27-nm protein. For this reason, our analysis focuses on NNDs as a robust lower limit estimate as marker jitter would only reduce the measured NNDs as compared to the true value.

The robustness of the NND estimate (with a prominent mode at ~40 nm) is also largely unaffected by missed RyRs (Figures S2B and S2C). Simulations suggested that a large fraction of missed RyRs would be expected to increase the tail of the 4ND histogram substantially, more than we observed, our data being consistent with a >80% RyR detection. In support of this estimate, we confirmed that labeling conditions maximized RyR labeling: experiments with additional permeabilization with Triton X did not increase observed RyR density and we chose a saturating concentration of primary antibodies in the experiments.

The observations based on our data have several consequences for RyR cluster biophysics. The density of RyRs within clusters is lower than expected for close packing, which reduces the cross-signaling between RyRs. In addition, there are sizable gaps in RyR clusters, up to 150 nm. Our phenomenological cluster assembly model showed such gaps can arise randomly and do not require any templating mechanisms. Such gaps can dramatically alter the diffusion patterns of the cytoplasmic Ca^{2+} experienced by RyRs, which dictate the probability of concerted cluster activation (Walker et al., 2014, 2015) and termination of local Ca^{2+} release (Laver et al., 2013). Walker et al. (2014) predicted that cluster excitability may be lowered in clusters containing gaps of ~50% of their internal area. Mathematical models need to be refined to capture the effect of such gaps on excitability using the data provided here.

Molecular Mapping of JPH2-RyR Co-clusters

The ability to conduct exchange-PAINT enabled high-resolution imaging of the relative location of RyRs and the accessory protein JPH2. JPH2 has been shown to be important for the maintenance of the junctional membrane geometry (Takeshima et al., 2000). In addition, it has been proposed that JPH2 can bind to RyRs and modulate their gating (Beavers et al., 2013). Exchange-PAINT provided a method to image both targets with the same dye but using orthogonal docking and imager pairs so that chromatic aberrations can be eliminated. Distance-based analysis of the exchange-PAINT data showed that a sub-population of JPH2 was within molecular distances to RyRs, as seen by a >5-fold increase in JPH2 densities in areas ≤50 nm around RyR puncta. Such an increase would not be expected if JPH2 were randomly distributed across RyR clusters (Figure S5A). In conjunction with previous immuno-precipitation studies (Beavers et al., 2013), this observation is compatible with some JPH2 bound to RyRs. Direct comparison with dSTORM data showed that this observation critically depended on the higher resolution of DNA-PAINT.

We also found that JPH2 proteins were present in some of the larger gaps observed within RyR clusters, showing that the gaps are not merely reflecting a topological boundary but are genuinely part of peripheral junctions.

With the ability to quantify markers and proteins using calibrated qPAINT analysis, we also conducted qPAINT analysis of exchange-PAINT data to estimate JPH2-RyR protein ratios. The use of qPAINT for protein ratio analysis is a natural combination of the exchange-PAINT and qPAINT concepts. The images show that adjacent clusters often exhibit considerably different ratios. The variable RyR:JPH2 ratios from cluster to cluster add further complexity to regulating units of calcium signaling. Our observations provide a molecular basis for a mechanism in which RyR clusters can be locally regulated by varying the abundance of adjacent structural and inhibitory proteins, i.e., cluster level regulation for signaling in complex cells.

The relative variation between adjacent cluster ratios is clearly visually apparent (Figure 5D) and is independent of antibody binding efficiency. Similarly, the shape of the histogram shown in Figure 5E is an invariant feature of our data regardless of corrections for JPH2 antibody binding efficiencies. In general, the estimation of local protein ratios in structures of biological interest, here co-clusters of the two proteins, lends itself more readily to biological interpretation than often difficult to interpret colocalization values. The ratio of JPH2 to RyRs was critical in a JPH2 overexpression model in which RyR cluster sizes were

OPEN
ACCESS
CellPress

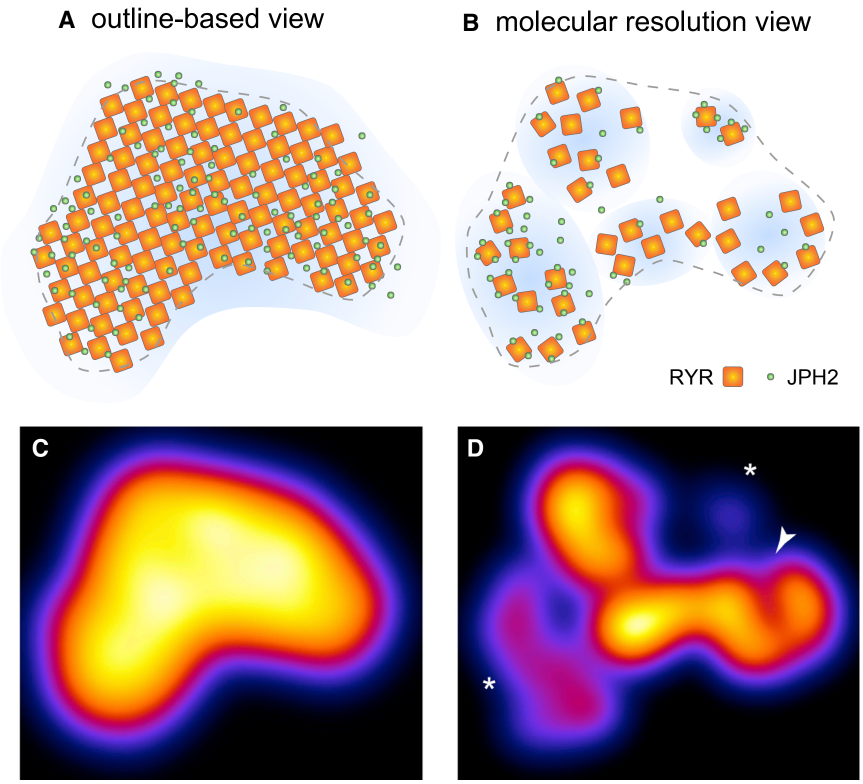

Figure 6. Schematic Comparison of Outline-Based and Molecular Scale Views of RyR Clusters

(A and B) Schematic comparison of the outline-based view of RyR clusters (A) and the molecular-scale maps (B). The latter account for variable arrangements of RyRs, gaps in arrays and differential co-clustering with JPH2.

(C) Schematic activation probability maps of clusters highlight uniform cluster activation probability in the regularly filled array compared to the heterogeneous activation of clusters (asterisks) and expected intra-nanodomain Ca^{2+} gradients (arrow) in the molecular-scale model.

greatly increased but excitability was stabilized by JPH2 (Munro et al., 2016).

The picture of RyR clusters and their regulation emerging from our data is schematically summarized in Figure 6. Where dSTORM informed outline-based views assumed a well-filled regular cluster with largely uniform activation properties, the molecular view shows a heterogeneous organization with differential inhibition by JPH2, compatible with a more complex regulation of RyR cluster activity.

Limitations

The size and binding location of markers on RyRs currently limit the precision with which we can judge the arrangement of RyRs and identify close packed areas unambiguously; the development of small markers binding to well-characterized locations of RyRs should be assisted by recently improved 3D structures of the cardiac ryanodine receptor (Peng et al., 2016).

Conclusions

The application of refined DNA-PAINT approaches to the molecular characterization of RyR clusters and JPH2-RyR co-clusters demonstrates the potential for molecular resolution quantitative imaging in complex biological samples. Using all optical methods, it is now possible to obtain data previously thought to be limited to the realm of electron microscopy. The optical data revealed that the density of RyRs in clusters is lower than expected for dense packing. Clusters follow an irregular assembly pattern compatible with spontaneous self-assembly and sizable gaps in clusters that will affect their calcium signaling. The high resolution of DNA-PAINT data also revealed a fraction of JPH2 in molecular proximity of RyRs and presents a super-resolution approach to *in situ* biochemistry to probe binding candidates in cells and tissues.

EXPERIMENTAL PROCEDURES

Further methods details are supplied in the Supplemental Experimental Procedures.

Cell Preparation

Live myocytes were isolated from hearts freshly dissected from wild-type male Wistar rats according to a protocol approved by the Animal Ethics Approval Committee of the University of Exeter. The suspension of myocytes was filtered and allowed to attach within glass coverslip chambers prior to fixation with 2% paraformaldehyde (w/v) and immunocytochemistry.

DNA-PAINT Probe Production

Both the "imager" and the "docking" strands (nucleotide sequence and terminal modifications specified in Table S1) were commercially synthesized by Eurofins UK. The two nucleotide designs of the P1 and P3 sequences were obtained from Jungmann et al. (2014). We opted for a direct thiolation to link a 5′ C6 amine of the docking strands and cysteines of the antibody. Respective docking strands were conjugated to either a goat anti-mouse immunoglobulin G (IgG) or a goat anti-rabbit IgG secondary antibody (Jackson ImmunoResearch, PA) or a primary antibody against RyR2, clone C3-33, using a Thunder-Link kit (Innova Biosciences, Cambridge) and spectrophotometric analysis to ensure an oligo: antibody conjugation ratio $\geq$1:1.

Imaging Probes, Reagents, and DNA-PAINT Protocol

All primary antibodies used here were previously characterized for both immunofluorescence imaging and *in vitro* analyses of the targets RyR2, JPH2, and CAV3. The RyR2 antibody was a mouse monoclonal IgG (catalog no. MA3-916; Thermo Scientific, DE). See details on antibodies under Supplemental Experimental Procedures.

For dSTORM imaging, a secondary antibody conjugated to Alexa Fluor 647 was used and imaged in switching buffer. In DNA-PAINT imaging, the sample was immersed in a buffer ("buffer C" as in Jungmann et al., 2014) containing typically 200 pM of either an ATTO 655 or ATTO 550 imager strand, complementary to the docking strand linked to the secondary antibody that we aimed to image. The imager strands reversibly bind to the complementary docking strands. Using TIRF illumination the fluorophores of hybridized imager strands were imaged, while transiently immobilized and appeared in the image as transient fluorescent spots whose shape matches the point spread function (PSF)

of the microscope in its focal plane. These events were recorded as a series of image frames (Figure S1).

Image Acquisition

Both DNA-PAINT and dSTORM images were acquired with a modified Ti-E inverted fluorescence microscope (Nikon, Japan) and fully adjustable custom-built optical illumination and detection paths optimized for TIRF and single-molecule localization. The focus was actively stabilized with a feedback system that used transmitted light imaging and image correlation to keep the focus constant by driving a piezo focusing device appropriately (tolerance $\leq$ 30 nm), similar to a method described in (McGorty et al., 2013). This stabilization system also tracked lateral sample movement that was digitally removed during analysis by correcting event coordinates. The image data were acquired and analyzed in real-time by a quad-core PC using the open source Python Microscopy Environment (PyME) software. Analysis of localization data from the sCMOS camera was performed with algorithms that correct for non-uniform sCMOS pixel properties as described recently (Lin et al., 2017).

Image Analysis

Basic Analysis and Grayscale Rendering

The frame data were analyzed in real-time (during acquisition) using the freely available PyME (http://python-microscopy.org/) developed by the consortium of co-authors. Localized event positions were rendered into a 16-bit grayscale tagged image file format (TIFF) image with a pixel scaling of 1 nm/pixel using an algorithm based on Delaunay triangularization (Baddeley et al., 2010) such that the pixel intensity was linearly proportional to the local density of localized markers, i.e., similar in its information content to a typical grayscale fluorescence micrograph albeit at higher spatial resolution.

Analysis of Punctate Nanoscale Densities

The punctate RyR labeling densities in the rendered images were detected using a custom-written analysis algorithm implemented in PyME. The centroids of puncta were used to count the number of observable RyRs within each cluster and to calculate the neighbor distances (e.g., Figure 3) through a Delaunay triangulation and to construct Euclidean distance maps, which were the basis for the distance-based density analysis of JPH2 labeling.

Area-Based Analysis of DNA-PAINT Images

The grayscale rendered DNA-PAINT images were also subjected to threshold-based analysis of RyR cluster areas as per previous dSTORM studies (Baddeley et al., 2009; Hou et al., 2015). 2D masks of the labeled regions were constructed and were used for computing the 2D area of RyR clusters and for performing a co-localization analysis between JPH2 and RyR in two-color DNA-PAINT data.

Statistical Methods

All mean and SDs of measurements presented in the manuscript were calculated using standard statistics routines in NumPy or Excel.

Quantification Using qPAINT

qPAINT Adaptation

qPAINT analysis was used as a second approach to estimate the number of RyRs underlying the recorded DNA-PAINT signals by statistical analysis of the fluorescence time series data as described recently (Jungmann et al., 2016). Temporal fluorescence time courses were reconstructed from the localized event data after segmenting events based on clustering. qPAINT analysis exploits the fact that due to the reversible binding between docking and imaging strands, the number of docking sites in a region is inversely proportional to the mean "dark time," i.e., the mean time between imager binding events, estimated through a histogram approach (Figure S4). A qPAINT index (i.e., inverse of the mean dark time) was estimated for each cluster or region, which were then calibrated by determining the qPAINT index of small clusters that contained only one or a few puncta.

Measurement of Protein Co-clustering Ratios in DNA Exchange-PAINT Series by qPAINT Analysis

The number N_R of RyRs and N_J of JPH proteins for each cluster by qPAINT analysis was estimated for larger RyR clusters (>15 RyRs, using the RyR cluster mask for segmentation of both RyR and JPH event data) in exchange-PAINT data. This included per channel calibrations of the RyR and JPH signals, respectively. The ratio $r_{J\text{-}R} = N_R / N_J$ was calculated for each large cluster in this way.

Simulation of Synthetic Data

Centroids of punctate RyR labeling densities were placed iteratively in either (1) a gridded organization at a fixed spacing (Figure S2A) or (2) randomly placed and at a variable spacing to the next nearest neighbor as described by a random sample from a normal distribution with a specified σ. The centroids of the puncta were then convolved with a 2D Gaussian model with a σ of 5 nm. The model was then used in the PyME software to generate single-molecule events within the labeled regions and rendered as grayscale images to match the imaging and localization parameters observed in the dSTORM and DNA-PAINT experimental data for simulation of dSTORM and DNA-PAINT imaging, respectively (Table S2).

SUPPLEMENTAL INFORMATION

Supplemental Information includes Experimental Procedures, six figures, and two tables and can be found with this article online at https://doi.org/10.1016/j.celrep.2017.12.045.

ACKNOWLEDGMENTS

The work was supported by the funding from the Human Frontier Science Program (RGP0027/2013), the Engineering and Physical Sciences Research Council of the UK (EP/N008235/1 awarded to C.S.), the Wellcome Trust (207684/Z/17/Z to I.J.), and the Royal Society of the UK (RG201601 to I.J.). We also thank Dr. Mike Deeks for helpful feedback on the manuscript.

AUTHOR CONTRIBUTIONS

Conceptualization, C.S., I.J., and L.D.M.; Methodology, I.J., A.H.C., L.D.M., and C.S.; Validation, I.J., A.H.C., T.L., and R.L.; Formal Analysis, I.J., A.H.C., and C.S.; Investigation, I.J. and A.H.C.; Resources, D.B., R.L., E.G., and C.H.; Writing, I.J., A.H.C., and C.S.; Visualization, I.J., C.S., and A.H.C.; Supervision, C.S.; Funding Acquisition, C.S. and I.J.

DECLARATION OF INTERESTS

The authors declare no competing interests.

Received: September 27, 2017
Revised: November 17, 2017
Accepted: December 12, 2017
Published: January 9, 2018

REFERENCES

Allen, D.G., Lamb, G.D., and Westerblad, H. (2008). Impaired calcium release during fatigue. J. Appl. Physiol. *104*, 296–305.

Asghari, P., Scriven, D.R., Sanatani, S., Gandhi, S.K., Campbell, A.I., and Moore, E.D. (2014). Nonuniform and variable arrangements of ryanodine receptors within mammalian ventricular couplons. Circ. Res. *115*, 252–262.

Baddeley, D., Jayasinghe, I.D., Lam, L., Rossberger, S., Cannell, M.B., and Soeller, C. (2009). Optical single-channel resolution imaging of the ryanodine receptor distribution in rat cardiac myocytes. Proc. Natl. Acad. Sci. USA *106*, 22275–22280.

Baddeley, D., Cannell, M.B., and Soeller, C. (2010). Visualization of localization microscopy data. Microsc. Microanal. *16*, 64–72.

Beavers, D.L., Wang, W., Ather, S., Voigt, N., Garbino, A., Dixit, S.S., Landstrom, A.P., Li, N., Wang, Q., Olivotto, I., et al. (2013). Mutation E169K in junctophilin-2 causes atrial fibrillation due to impaired RyR2 stabilization. J. Am. Coll. Cardiol. *62*, 2010–2019.

Betzig, E., Patterson, G.H., Sougrat, R., Lindwasser, O.W., Olenych, S., Bonifacino, J.S., Davidson, M.W., Lippincott-Schwartz, J., and Hess, H.F. (2006). Imaging intracellular fluorescent proteins at nanometer resolution. Science *313*, 1642–1645.

Cannell, M.B., and Kong, C.H. (2012). Local control in cardiac E-C coupling. J. Mol. Cell. Cardiol. *52*, 298–303.

Dai, M., Jungmann, R., and Yin, P. (2016). Optical imaging of individual biomolecules in densely packed clusters. Nat. Nanotechnol. *11*, 798–807.

Fabiato, A. (1985). Time and calcium dependence of activation and inactivation of calcium-induced release of calcium from the sarcoplasmic reticulum of a skinned canine cardiac Purkinje cell. J. Gen. Physiol. *85*, 247–289.

Franzini-Armstrong, C., and Protasi, F. (1997). Ryanodine receptors of striated muscles: a complex channel capable of multiple interactions. Physiol. Rev. *77*, 699–729.

Franzini-Armstrong, C., Protasi, F., and Ramesh, V. (1999). Shape, size, and distribution of Ca(2+) release units and couplons in skeletal and cardiac muscles. Biophys. J. *77*, 1528–1539.

Gambin, Y., Ariotti, N., McMahon, K.A., Bastiani, M., Sierecki, E., Kovtun, O., Polinkovsky, M.E., Magenau, A., Jung, W., Okano, S., et al. (2013). Single-molecule analysis reveals self assembly and nanoscale segregation of two distinct cavin subcomplexes on caveolae. eLife *3*, e01434.

Groff, J.R., and Smith, G.D. (2008). Ryanodine receptor allosteric coupling and the dynamics of calcium sparks. Biophys. J. *95*, 135–154.

Heilemann, M., van de Linde, S., Schüttpelz, M., Kasper, R., Seefeldt, B., Mukherjee, A., Tinnefeld, P., and Sauer, M. (2008). Subdiffraction-resolution fluorescence imaging with conventional fluorescent probes. Angew. Chem. Int. Ed. Engl. *47*, 6172–6176.

Hess, S.T., Girirajan, T.P.K., and Mason, M.D. (2006). Ultra-high resolution imaging by fluorescence photoactivation localization microscopy. Biophys. J. *91*, 4258–4272.

Hou, Y., Jayasinghe, I., Crossman, D.J., Baddeley, D., and Soeller, C. (2015). Nanoscale analysis of ryanodine receptor clusters in dyadic couplings of rat cardiac myocytes. J. Mol. Cell. Cardiol. *80*, 45–55.

Jungmann, R., Avendaño, M.S., Woehrstein, J.B., Dai, M., Shih, W.M., and Yin, P. (2014). Multiplexed 3D cellular super-resolution imaging with DNA-PAINT and Exchange-PAINT. Nat. Methods *11*, 313–318.

Jungmann, R., Avendaño, M.S., Dai, M., Woehrstein, J.B., Agasti, S.S., Feiger, Z., Rodal, A., and Yin, P. (2016). Quantitative super-resolution imaging with qPAINT. Nat. Methods *13*, 439–442.

Laver, D.R., Kong, C.H., Imtiaz, M.S., and Cannell, M.B. (2013). Termination of calcium-induced calcium release by induction decay: an emergent property of stochastic channel gating and molecular scale architecture. J. Mol. Cell. Cardiol. *54*, 98–100.

Lin, R., Clowsley, A.H., Jayasinghe, I.D., Baddeley, D., and Soeller, C. (2017). Algorithmic corrections for localization microscopy with sCMOS cameras - characterisation of a computationally efficient localization approach. Opt. Express *25*, 11701–11716.

Manita, S., and Ross, W.N. (2009). Synaptic activation and membrane potential changes modulate the frequency of spontaneous elementary Ca2+ release events in the dendrites of pyramidal neurons. J. Neurosci. *29*, 7833–7845.

McGorty, R., Kamiyama, D., and Huang, B. (2013). Active microscope stabilization in three dimensions using image correlation. Opt. Nanoscopy *2*, 1–7.

Mikhaylova, M., Cloin, B.M., Finan, K., van den Berg, R., Teeuw, J., Kijanka, M.M., Sokolowski, M., Katrukha, E.A., Maidorn, M., Opazo, F., et al. (2015). Resolving bundled microtubules using anti-tubulin nanobodies. Nat. Commun. *6*, 7933.

Munro, M.L., Jayasinghe, I.D., Wang, Q., Quick, A., Wang, W., Baddeley, D., Wehrens, X.H.T., and Soeller, C. (2016). Junctophilin-2 in the nanoscale organisation and functional signalling of ryanodine receptor clusters in cardiomyocytes. J. Cell Sci. *129*, 4388–4398.

Peng, W., Shen, H., Wu, J., Guo, W., Pan, X., Wang, R., Chen, S.R.W., and Yan, N. (2016). Structural basis for the gating mechanism of the type 2 ryanodine receptor RyR2. Science *354*, aah5324.

Rust, M.J., Bates, M., and Zhuang, X. (2006). Sub-diffraction-limit imaging by stochastic optical reconstruction microscopy (STORM). Nat. Methods *3*, 793–795.

Shim, S.H., Xia, C., Zhong, G., Babcock, H.P., Vaughan, J.C., Huang, B., Wang, X., Xu, C., Bi, G.Q., and Zhuang, X. (2012). Super-resolution fluorescence imaging of organelles in live cells with photoswitchable membrane probes. Proc. Natl. Acad. Sci. USA *109*, 13978–13983.

Szymborska, A., de Marco, A., Daigle, N., Cordes, V.C., Briggs, J.A., and Ellenberg, J. (2013). Nuclear pore scaffold structure analyzed by super-resolution microscopy and particle averaging. Science *341*, 655–658.

Takeshima, H., Komazaki, S., Nishi, M., Iino, M., and Kangawa, K. (2000). Junctophilins: a novel family of junctional membrane complex proteins. Mol. Cell *6*, 11–22.

Walker, M.A., Williams, G.S., Kohl, T., Lehnart, S.E., Jafri, M.S., Greenstein, J.L., Lederer, W.J., and Winslow, R.L. (2014). Superresolution modeling of calcium release in the heart. Biophys. J. *107*, 3018–3029.

Walker, M.A., Kohl, T., Lehnart, S.E., Greenstein, J.L., Lederer, W.J., and Winslow, R.L. (2015). On the Adjacency Matrix of RyR2 Cluster Structures. PLoS Comput. Biol. *11*, e1004521.

Winslow, R.L., Tanskanen, A., Chen, M., and Greenstein, J.L. (2006). Multi-scale modeling of calcium signaling in the cardiac dyad. Ann. N Y Acad. Sci. *1080*, 362–375.

Xu, K., Zhong, G., and Zhuang, X. (2013). Actin, spectrin, and associated proteins form a periodic cytoskeletal structure in axons. Science *339*, 452–456.

Yin, C.-C., and Lai, F.A. (2000). Intrinsic lattice formation by the ryanodine receptor calcium-release channel. Nat. Cell Biol. *2*, 669–671.

Cell Reports, Volume *22*

Supplemental Information

True Molecular Scale Visualization of Variable Clustering Properties of Ryanodine Receptors

Izzy Jayasinghe, Alexander H. Clowsley, Ruisheng Lin, Tobias Lutz, Carl Harrison, Ellen Green, David Baddeley, Lorenzo Di Michele, and Christian Soeller

Supplementary Material

Supplementary Figures

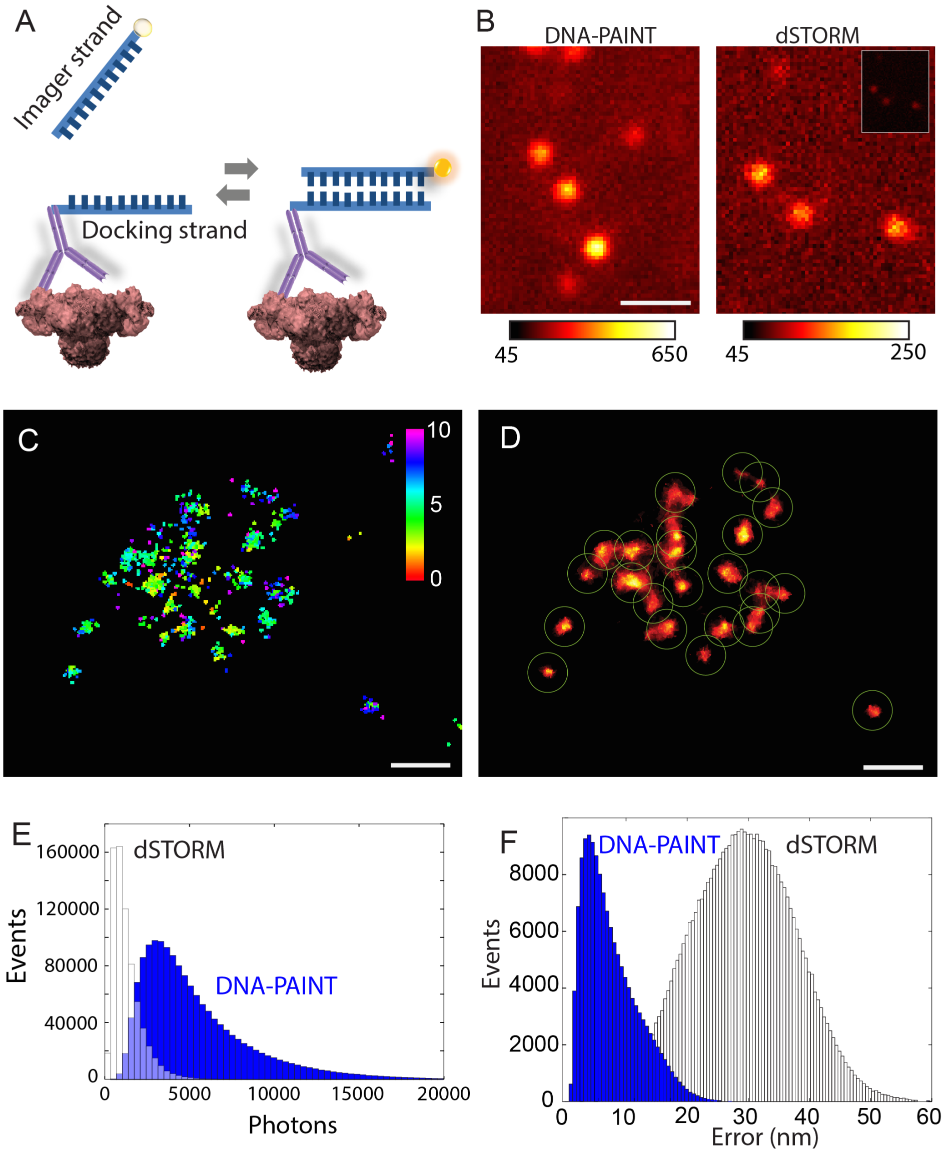

Supplementary Fig S1: DNA-PAINT implementation for improved RyR imaging. Related to Figure 1. (A) The DNA-PAINT principle works by the thermally-driven stochastic and transient hybridisation of the imager strand oligonucleotide conjugated to a fluorophore to the docking strand oligonucleotide marking the target RyR within the TIRF excitation field. **(B)** Binding events were visualised in a series of TIRF images as bright fluorescent spots (single molecule events, left) which were similar in their two-dimensional size and shape to dSTORM events (right), albeit brighter (detected photons per event). For comparison, a facsimile of a dSTORM frame is shown in the inset with the same colour-scaling as the DNA-PAINT image. **(C)** These events were used for localising the marker positions into a series of points whose spatial patterns were characteristic of nanometre-scale punctate structures (localisation error shown in colour-coded bar; scale in nm). **(D)** The point data were rendered into a 2D greyscale 32-bit image which shows the punctate morphology. To discretise these punctate densities, the centroids of each punctum were calculated with an automated puncta detection algorithm. Each detected punctum is indicated with centred circles of 50 nm radii. **(E)** A frequency histogram analysis of typical DNA-PAINT and dSTORM datasets revealed a 3-10 fold higher mode in the estimated total photons collected per event in DNA-PAINT. **(F)** The higher event intensity contributed to a reduced event localisation error in DNA-PAINT events as shown in the frequency histogram from a corresponding image series. Scale bars: (B) 1 µm, (C & D) 50 nm.

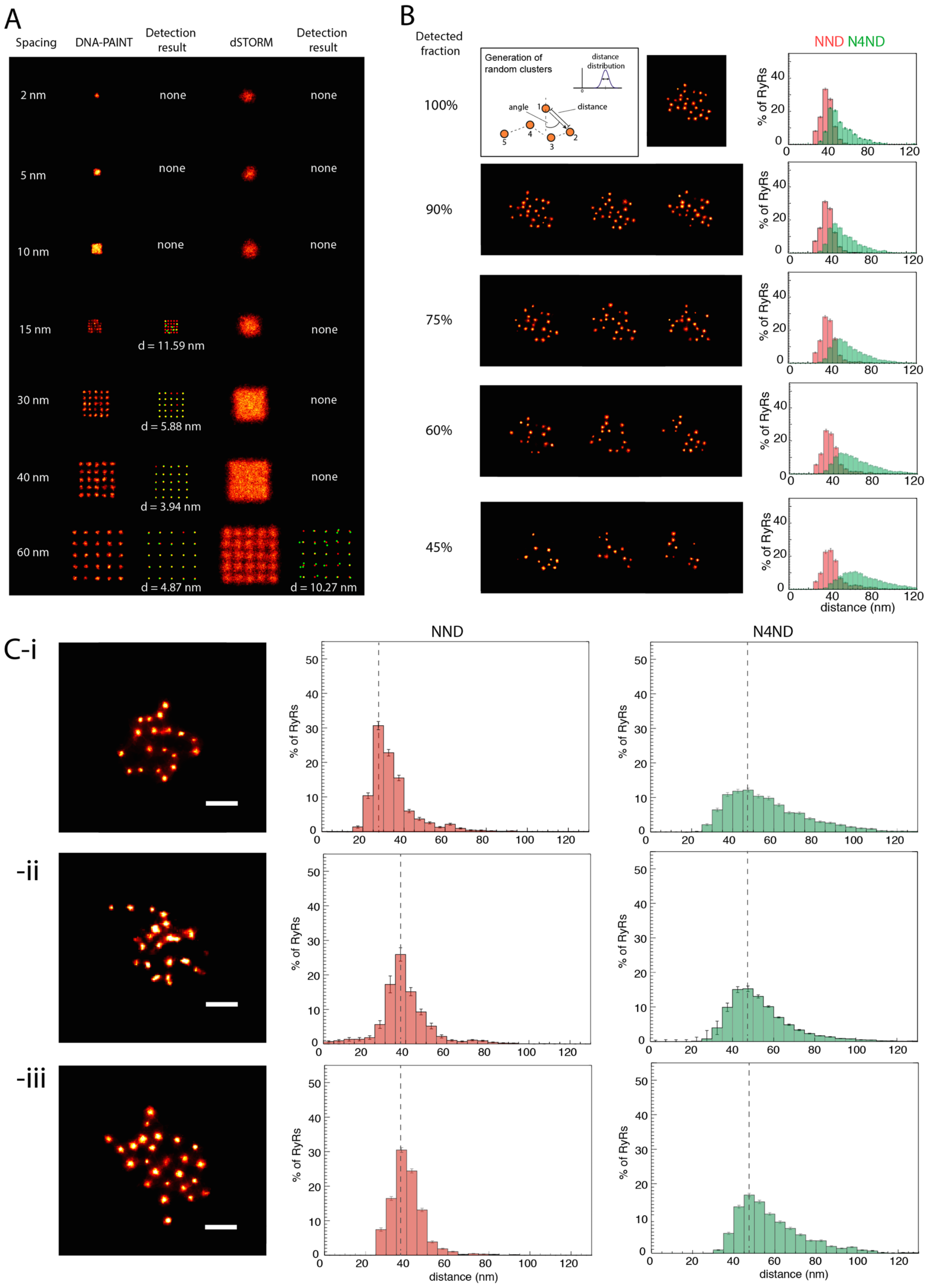

Supplementary Fig S2: Simulation of DNA-PAINT images of RyR clusters as a test model. Related to Figure 4. (A) Investigating the ability to resolve the protein distribution in clusters as a function of the localisation precision and associated imaging parameters utilising simulated target spacing patterns. Greyscale, simulated super-resolution DNA-PAINT (first column) and dSTORM (third) images of a cluster with targets on a square grid at regular spacings (specified on left) were simulated using imaging parameters as in Table S2. The square outline of the cluster is faithfully reported when the target spacing is >5x5 nm for DNA-PAINT parameters and >30x30 nm for dSTORM imaging parameters. The centroids of the punctate marker densities were determined with the method shown in Fig S1 (**green** circles) and overlaid with the centroids used for the simulation (**red**), 2nd and 4th columns. The markers are only resolvable when the marker spacing is >10x10 nm for DNA-PAINT and reliably detectable (efficiency ~84%) when the spacing is >15x15 nm. In dSTORM data, markers within the cluster are not resolvable until spacing is ~60x60 nm, yet including errors in centroid detection. The mean distance *d* between original versus detected target centroid is stated below each panel. **(B)** To investigate whether incomplete labelling of RyRs within a given cluster can distort the measured distribution, clusters with 25 RyRs at variable detection efficiencies (right

column) and nearest neighbour distances (NND) mean = 40 nm, sigma = 7.4 nm (second panel on top row), were simulated. Examples of Monte Carlo simulations with RyR detection efficiency of 90 %, 75 %, 60 % and 45 % are shown in middle panels. The corresponding NND (**red**) and the average of the distances to the nearest four neighbours (N4ND; **green**) are shown on right. Note how the reduction in the detection efficiency hardly altered the detected NND; however, a progressive broadening of the N4ND occurs with the mode being right shifted from 45 nm to 50, 60 and 65 nm, respectively. **(C-i)** To investigate the ability to distinguish between narrow RyR spacing and low labelling efficiency versus wider spacing but high labelling efficiency, a simulated cluster (left panel) from an ensemble generated with a mean RyR spacing of 30 nm and a detection efficiency of 50 % is shown. The NND distribution (middle panel) is only weakly affected by missed RyRs, the mode is still at ~30 nm. The N4ND distance measure is more sensitive and right-shifted. **(C-ii)** By contrast, our measurements of actual clusters exhibit a NND mode at ~40 nm, and N4ND mode at ~50 nm. **(C-iii)** The experimental observations are consistent with a cluster model that has a mean distance between nearest RyRs of 40 nm (middle panel), an example cluster is shown in the left panel. The N4ND (right panel) agrees well with experimental observations when a large detection efficiency >80% is assumed. Scale bars: 100 nm. Error bars: (B, C) NND & N4ND histograms – SEM for n=10 simulated clusters.

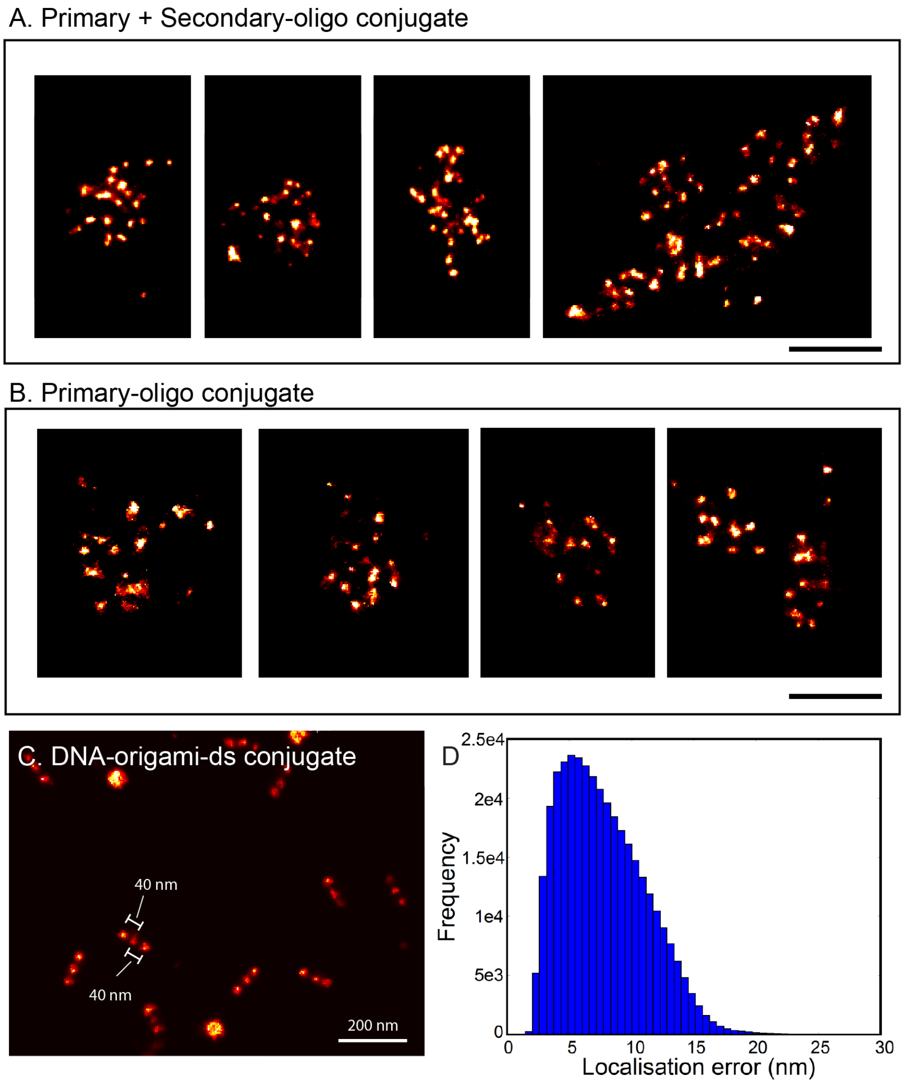

Supplementary Fig S3: Control experiments with DNA-PAINT samples. Related to Figure 2. To verify whether the morphology of the RyR cluster can be reproduced independently of the nature of the marker of the target protein (i.e. whether the morphology was dependent on the primary and secondary antibody interaction), a comparison was made between two labelling methods: **(A)** A gallery of RyR clusters resolved with DNA-PAINT where the RyRs were labelled with a monoclonal anti-RyR IgG primary antibody and then an anti-IgG secondary antibody conjugate of the docking strand. **(B)** A similar series of images of RyR clusters where the RyRs were labelled with the anti-RyR primary antibody directly conjugated to the docking strand revealed a similar punctate morphology, confirming that the punctate labelling densities observed in the two labelling approaches are independent of the size of the labelling complexes. **(C)** To demonstrate the resolvability of targets with 40 nm spacings with the DNA-PAINT protocol, we imaged DNA-origami molecular rulers in a Gattaquant 40R-HiRes slide. The larger round structures are additional, structurally different DNA-origami structures provided for drift correction. Drift correction was performed using transmitted light as described in the methods. **(D)** The estimated localisation errors of the events in the frame data used to generate these images were similar in magnitude to those in the experimental data in myocytes. Scale bars: 200 nm.

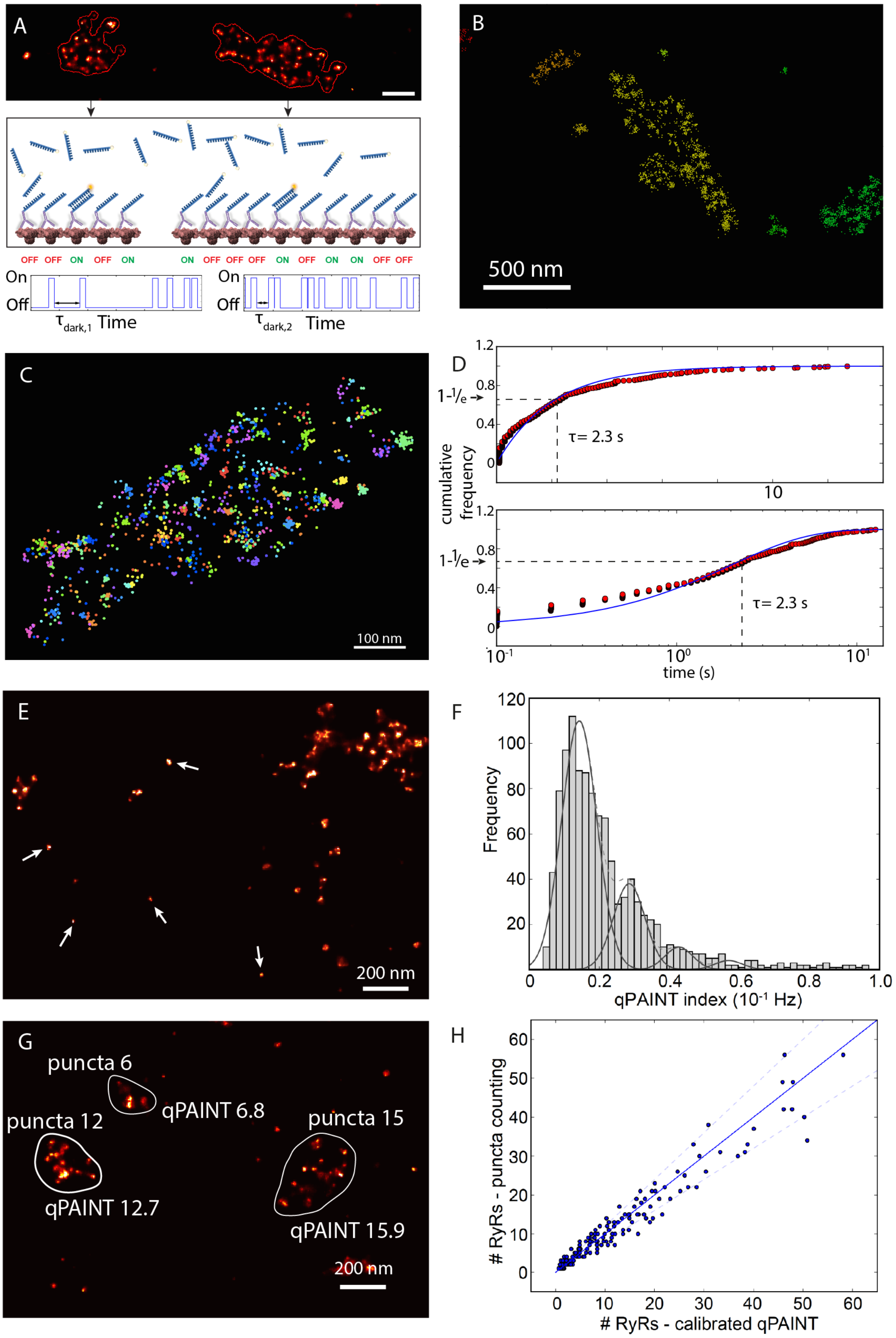

Supplementary Fig S4: Methodology of modified qPAINT analysis to verify RyR cluster size independently of the morphological analysis of the punctate labelling densities. Related to Figure 3. (A) qPAINT analyses the temporal properties of single molecule event detections recorded at a given cluster. Two clusters are shown, one larger than the other. The rate at which imagers bind to RyRs in a cluster is proportional to the number of RyRs in the cluster; the average dark time between detected binding events is measured (lower panels) which shortens as more labelled RyRs provide binding sites for imagers in solution. The RyR numbers are proportional to the inverse of the dark time but to obtain absolute numbers a calibration must be performed to determine the constant of

proportionality. **(B)** A view of detected events (dots) in a section of a DNA-PAINT data set. Events are segmented and coloured according to the cluster they belong to. **(C)** A single cluster is shown; events belonging to this cluster are coloured according to the time at which they were detected. **(D)** From the events belonging to a cluster and their time stamps (frame number) a cumulative histogram of dark times is reconstructed (red dots) which is fit with the expected distribution for a first order binding reaction (see Methods). The average dark time is located at the 1-1/e point of the curve, here $\tau = 2.3$ s. Top panel: linear time scale; bottom panel: logarithmic time scale. **(E)** Small clusters, visually containing only one or a few puncta (arrows), were identified by apparent cluster area (cluster area < 10,000 nm^2) and their dark times and qPAINT indices were determined. **(F)** The qPAINT index (inverse of the measured dark time) histogram exhibited peaks at multiples of a qPAINT index of $0.14 \cdot 10^{-1}$ Hz. We visually confirmed that the clusters corresponding to the fundamental peak typically contained a single punctum. The calibration value was used to calibrate the corresponding data set and obtain estimated RyR numbers for each cluster as the ratio of the cluster qPAINT index q_i and the calibration value q_{i1}. Calibrations were conducted for each data set using a similar in-situ calibration. **(G)** A small section of a DNA-PAINT image of RyR labelling shows three clusters and the comparison of RyR number estimates obtained by puncta counting and calibrated qPAINT analysis indicates good agreement. **(H)** The correlation between RyR cluster sizes determined by puncta counting and qPAINT analysis is relatively tight. The solid line is the line of identity, the dashed lines show ratios of 1.2 and 0.8, respectively. Each data point represents a single cluster.

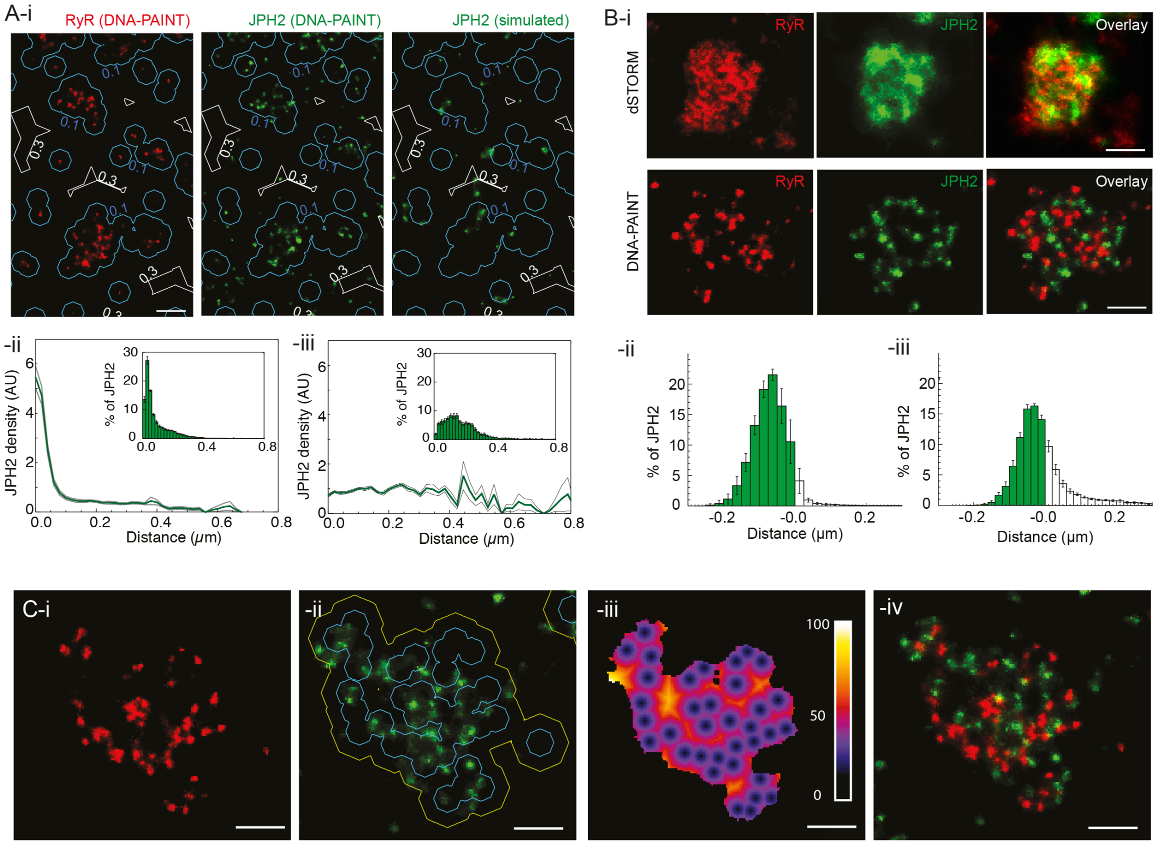

Supplementary Fig S5: Analysis of the nanoscale co-localisation of JPH2 within RyR clusters. Related to Figure 5. (A-i) A distance-based co-localisation was applied (as in Jayasinghe et al. 2012) to quantify the nature of the co-localisation between RyR (left) and JPH (centre). To verify whether the observed JPH2 localisation distribution showed preferential co-clustering with RyR, we also simulated a DNA-PAINT image of a randomly distributed marker population of JPH2 whose 2D position was assigned in the image based on a random uniform distribution (A- right). The Euclidean distance map constructed from the centroids of the RyR puncta (0.1 µm and 0.3 µm contours) were overlaid with the DNA-PAINT images which showed that both RyR (left panel) and experimental JPH2 distributions (middle panel) were intimately co-clustered within 100 nm distance bands while the simulated JPH2 data (right panel) showed this to a lesser extent. **(A-ii)** Histogram analysis of the density of JPH2 labelling as a function of the distance from the centroids of the single RyR puncta shows a high density of JPH2 markers within 50 nm of the RyRs. At distances beyond 100 nm, the residual density was approximately uniform but 5-10 times lower. Analysis of the percentage of the integrated labelling density as a function of this distance scale (inset) showed that >86 % of all the JPH2 events were localised within 200 nm of the RyR puncta. **(A-iii)** The same density-distance analysis performed by replacing the experimental JPH2 image with the simulated JPH2 image where JPH molecules were randomly placed exhibited essentially a uniform density distribution, as expected. The random placement of JPH2 diminished the high percentage of JPH2 organised closely to RyR (only ~58% of the JPH2 labelling localised within 200 nm) confirming that the experimentally observed JPH2 distribution is not compatible with JPH2 random placement relative to RyRs. **(B-i)** From dSTORM (top row) and DNA-PAINT (second row) data of RyR (red) and JPH2 labelling (green; overlays also shown on left), we analysed the percentage of integrated JPH2 labelling as a function of the Euclidean distance from the edge of each RyR-labelled region. Histograms reflect analyses performed on **(B-ii)** dSTORM and **(B-iii)** DNA-PAINT data. The shaded area (distances <0.0) of the percentage histogram of the integrated JPH2 labelling dSTORM data plotted as a function of the distance from the edge of each RyR cluster reports 72.0 ± 2.5% (n=5 cells; 2 animals) of this labelling within the RyR mask (see also previous analysis by Jayasinghe et al. 2012). In the DNA-PAINT analyses the percentage histogram equivalent to above, from an analysis based on the cluster edge, revealed a smaller total fraction (~70%) of JPH2 now co-localising with RyR. This decrease is expected due to the higher resolution in the DNA-PAINT data but we note that the dSTORM data do not detect the increase in JPH2 density close to RyRs, consistent with a lower effective resolution. **(C-i)** Given the non-uniform arrangement of RyR (Fig 3), we examined whether any of the JPH2 densities could be localised to the regions within the RyR cluster that were devoid of RyRs. Shown, are exchange-PAINT images of RyR (red) and **(C-ii)** JPH2 (green) acquired from a rat ventricular myocyte. Overlay of the JPH2 image with a contour map (50 nm in blue; 100 nm in yellow) of the Euclidean distance from the centroids

of RyR puncta illustrates that a large fraction of the JPH2 are localised within the 50 nm bands (i.e. most likely bound to RyRs). A smaller fraction was observed between the yellow and blue contours signifying JPH2 which are resident to the junction, but nevertheless unlikely binding partners of RyR. **(C-iii)** The latter population of JPH align strongly with the yellow regions of the Euclidean distance colour map (scale in nm) reporting continuous regions devoid of RyR. **(C-iv)** Domains of potentially specialised localisation of JPH2 are also notable in the two-colour overlay of DNA-PAINT images unlike the dSTORM images (panel B-i). Scale bars: 200 nm. Error bars: (Aii, iii) SEM for n=8 cells; (Bii) SEM for n=5 cells; (Biii) SEM for n=8 cells.

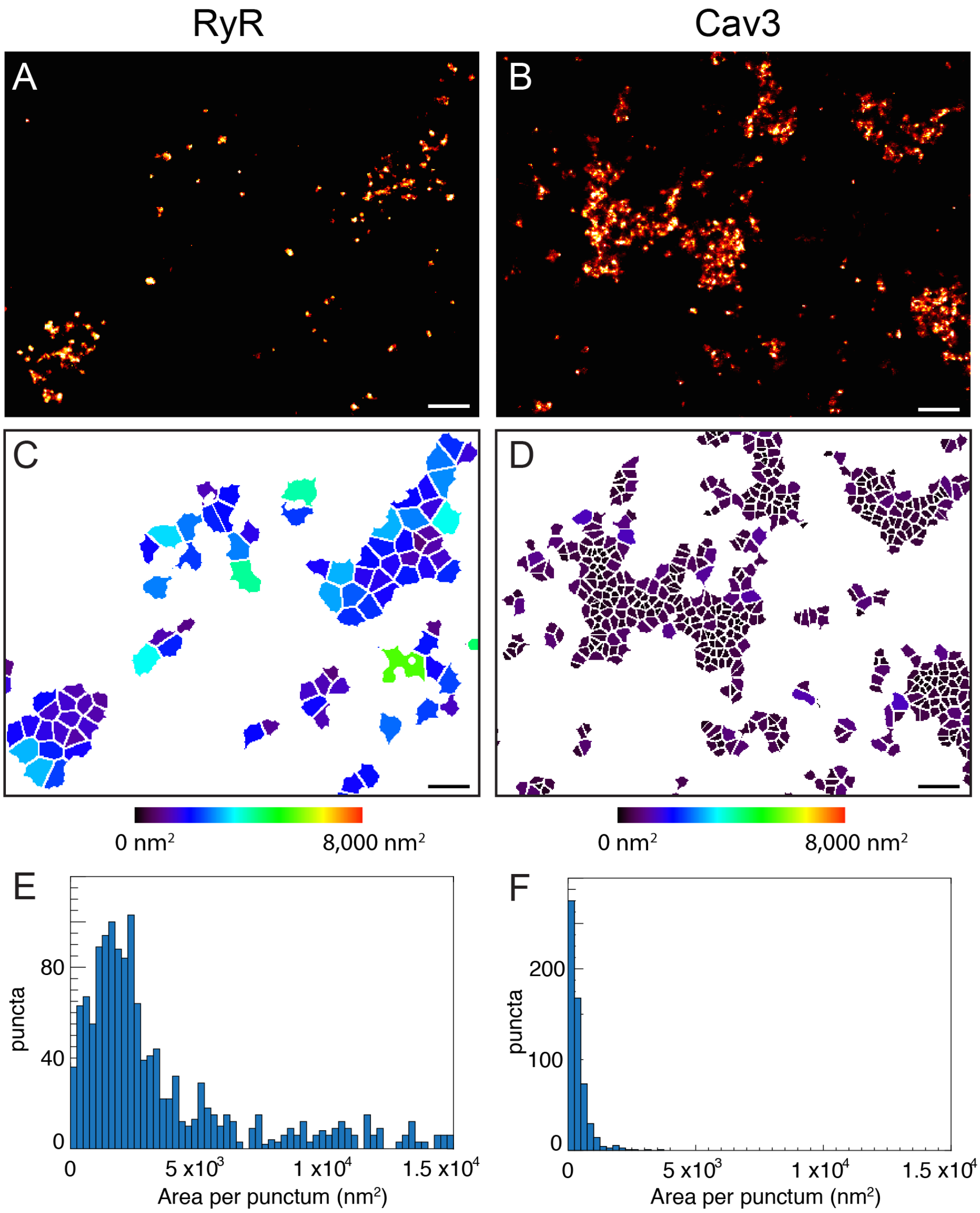

Supplementary Fig S6: The packing distances of antibody labelling densities are not limited by the labelling system. Related to Figure 6. To examine whether the apparent marker binding density or the resolvable marker spacings are compatible with high density protein distributions, we examined DNA-PAINT data labelled against the densely-organised plasma membrane protein Caveolin-3. **(A)** Typical DNA-PAINT images of peripheral cluster labelling of mouse monoclonal anti-RyR2 antibodies. **(B)** DNA-PAINT images of mouse monoclonal anti-Caveolin-3 antibodies in similar regions. Note the punctate labelling morphology in both label distributions. To quantify the proximity of packing, the centroids of the punctate densities were computed (as in Fig S1D) and subjected to Voronoi partitions to visualise the "areas" per punctum for both the **(C)** RyR and **(D)** Caveolin-3 label distribution. Each of the Voronoi partitions is coloured according to the size of the area around a punctum (see calibration). The frequency distributions of the Voronoi partition areas for **(E)** RyR antibodies and **(F)** Caveolin-3 antibodies confirm the smaller partitions in Caveolin-3 images compared to RyR. This observation showed that our methodology was able to detect higher punctate labelling densities than observed with RyR markers and that the observed RyR morphology or distance measurements are unlikely to be limited by marker access to the target or the resolution of the DNA-PAINT images. Average densities of puncta in RyR data was one per 4314.87 ± 1332.47 nm^2 while that in caveolin-3 was one per 1466.95 ± 455.5 nm^2. Scale bars: 50 nm.

Supplementary Tables

DNA-PAINT imaging channel	Imager strand	Docking strand
561 nm excitation/ orange emission for P1 configuration	5'–CTAGATGTAT-3'-ATTO550	C6-amine-5'-TTATACATCTA–3'-Cy5
642 nm excitation/ far red emission for P1 configuration	5'–CTAGATGTAT-ATTO655	C6-amine-5'-TTATACATCTA–3'-Cy3.5
561 nm excitation/ orange emission for P3 configuration	5'–GTAATGAAGA-3'-ATTO550	C6-amine-5'-TTTCTTCATTA–3'-Cy5
642 nm excitation/ far red emission for P3 configuration	5'–GTAATGAAGA-ATTO655	C6-amine-5'-TTTCTTCATTA–3'-Cy3

Table S1: The nucleotide sequence and terminal modifications of the P1 and P3 'imager'/'docking' strand pairs used for specific imaging configurations. Related to Experimental Procedures.

**Parameter**	**dSTORM**	**DNA-PAINT**
Events per pixel	1.0	1.0
Event intensity (photons)	1000	8000
Event background (photons)	200	600
Event duration (frames)	3	6
Events per marker	2	5
Number of frames	30000	10000
Rendering localisation error filter threshold	30 nm	10 nm

Table S2: The parameters adopted for generating synthetic localisation data in Python Microscopy Environment software. Related to Figure 4 and Supplementary Figure 2.

Supplementary experimental details

Animals, sample preparation and immunocytochemistry

Male Wistar rats weighing 250-350 g were euthanised humanely with lethal dose (100 mg per kg of body weight) of sodium pentabarbitone according to a Schedule 1 protocol in compliance with the 1986 Animals (Scientific Procedures) Act, approved by the Animal Ethics Approval Committee of the University of Exeter. The hearts were quickly excised, cannulated at the aorta and retrogradely perfused with calcium (Ca^{2+})-free Tyrode's solution using a Langendorff perfusion protocol described previously (1). After 5 minutes, the perfusate was switched to a Tyrode's solution containing 200 μM $CaCl_2$, 1 mg/mL Collagenase-II (CLS2, Worthington Biochemical, NJ) and 0.1 mg/mL Protease-I (Sigma-Aldrich, MO). Following 10-15 minutes of perfusion, the ventricles were dissected into fresh Tyrode's solution containing 200 μM $CaCl_2$, diced with sharp scissors and gently triturated to liberate isolated ventricular myocytes. The suspension of myocytes was filtered and transferred in aliquots into the custom-made imaging chambers whose bottom consisted of a No 1.5 glass coverslip. Coverslips were previously cleaned with concentrated NaOH in methanol, rinsed with fresh distilled water and coated overnight at room temperature with a laminin solution (11.9 μg/mL, diluted from #23017-015, Life Technologies) to allow strong attachment of myocytes. After incubation in the chambers for 90 minutes at 30° C, the myocytes attached to the coverslip were fixed by replacing the Tyrode's solution and excess cell suspension with 2 % (w/v) paraformaldehyde (Sigma-Aldrich) in phosphate buffered saline (PBS) at pH of 7.4. After incubating in room temperature for 12 minutes, the fixative was replaced with fresh PBS and allowed to wash for another 12 minutes. This washing was repeated once more before storing the chambers in fresh PBS containing 0.1% sodium azide at 4° C until the immunocytochemistry step.

The immuno-labelling of specific targets of fixed cells was performed according to a protocol described previously (1). Fixed myocytes which were pre-adhered to laminin-coated chambers were briefly permeabilised with 0.1% Triton-X-100 (Sigma-Aldrich) diluted (v/v) in PBS for 10 minutes and then blocked with PBS containing 10% normal goat serum (Life Technologies) for 1 hour at room temperature. The primary antibody was diluted in an incubation buffer containing 1x PBS, 0.5% bovine serum albumin (w/v; Sigma-Aldrich) and 1% normal goat serum and applied overnight at 4° C. Samples were washed three times with fresh PBS at 30 minute intervals and incubated for two hours at room temperature. Secondary antibodies were dissolved in PBS containing 1 mM ethylenediaminetetraacetic acid (EDTA) and applied for 2 hours at room temperature before three further washing steps.

DNA-PAINT probe production

Both the 'imager' and the 'docking' strands (nucleotide sequence and terminal modifications detailed in Table S1) were commercially synthesised and HPLC purified using the custom DNA oligos service by Eurofins UK. The two nucleotide designs of the P1 and P3 sequences were obtained from (2).

Rather than using a biotin-streptavidin linker (which is large and can give rise to undesirable non-specific labelling with endogenous biotins in the myocytes) as done in the original DNA-PAINT study (2), we opted for a direct thiolation which links a 5' C6 amine of the docking strands and cysteines of the antibody. In addition, the conjugated docking strands contained a 3' fluorophore which has the advantage that the conjugated antibody markers carry a fluorochrome, allowing us to confirm positive staining. This fluorophore does not interfere with later DNA-PAINT imaging since generally the 3'-fluorophore was selected spectrally distinct from the imager fluorophore. In rare cases where there was overlap between docking strand fluorophore and imager fluorophore emission a period of intense illumination was used to remove any background from docking strand fluorophores by permanent photobleaching before commencing DNA-PAINT acquisition. Respective docking strands were conjugated to either a goat anti-mouse IgG or a goat anti-rabbit IgG secondary antibody (affinity purified, azide-free form from Jackson Immunoresearch, PA) using a Thunder-Link® kit (Innova Biosciences, Cambridge). In some experiments a primary antibody against RyR2, clone C3-33 – see below, was conjugated directly to DNA-PAINT docking strands. The conjugated antibody was purified from unconjugated docking strands by use of a Thunder-Link® kit-based precipitation step and centrifugation at 13,000g. The relative concentration of the antibody and the docking strand (by the 3'-fluorophore) in the conjugate were determined with the use of a NanoDrop 2000 spectrophotometer (Thermo Scientific). Only antibody samples with an oligo : antibody conjugation ratio ≥ 1:1 were used for sample labelling.

Antibodies

The mouse monoclonal anti-ryanodine receptor-2 IgG from Clone C3-33 (Cat# MA3-916; Thermo Scientific, DE) was used for labelling the ryanodine receptors in the fixed cardiomyocytes. This antibody is well characterised for its high specificity of binding(3) and has been established as one of the consistently reliable probes for diffraction-limited (1, 4) and super-resolution microscopies (5, 6) of RyR2. As a qualitative comparison, a monoclonal pan RyR antibody of clone 34C (Cat# MA3-925; Thermo Scientific) was used to obtain independent DNA-PAINT images of peripheral RyR2 distributions in the myocytes. For staining Caveolin-3, a mouse monoclonal antibody

from BD Transduction (Cat# 610421) was used while a custom-made rabbit polyclonal antibody described previously (7) was used against Juctophilin-2 (JPH2). Alexa Fluor 647 (Cat# A-21236; Thermo Scientific) secondary antibodies were used for dSTORM experiments and imaged in PBS based switching buffer containing 10% (w/v) glucose, 100 mM 2-Mercaptoethylamine, Glucose oxidase (0.5 mg/mL) and catalase (0.05 mg/mL), all from Sigma.

Details of imaging apparatus

Both DNA-PAINT and dSTORM images were acquired with a modified Nikon Ti-E inverted fluorescence microscope (Nikon, Japan) and fully-adjustable custom-built optical illumination and detection paths, see also Fig.1-S1. These custom-optics allowed the laser to be conveniently focused into the sample in an angle either exceeding the critical angle (for TIRF imaging) in DNA-PAINT experiments or at a shallower angle for oblique illumination (8) for dSTORM experiments. This produced an ~20 µm-wide illumination spot which was sufficiently large to observe the single molecule events (PAINT or dSTORM) from peripheral couplons near the surface of the cell which was in contact with the coverslip as performed previously (5). The laser was focused into the sample with a 1.49NA 60x oil immersion objective fixed onto a piezo focus z-drive (P-725 Physik Instrumente, Germany) which was mounted rigidly within a custom-made aluminium bracket to minimise any thermal drift of the focal plane.

Illumination for the tracking system that stabilises residual thermal drift was provided by the white light bulb of the microscope condenser illuminator, similar to a method described in (9). A band-pass filter (Semrock FF02-447/60) was inserted to select only the blue light which is decoupled from the fluorescence path by a high-flatness dichroic (Semrock FF660-Di02). During data acquisition, images using the blue transmitted light are captured by a tracking camera (Thorlabs DCC3240N). A matching band-pass filter is inserted in front of the camera to clean up any stray light from the laser. Using image correlation to measure focus changes the focus was kept constant by driving a PiFoc piezo focusing device appropriately (tolerance <= 30 nm). The focus stabilisation system also tracked lateral sample movements which were not actively corrected during acquisition but instead digitally removed during analysis by subtracting the recorded lateral drift time course from event coordinates.

Prior to DNA-PAINT imaging, the presence of the primary and secondary antibodies was confirmed by the fluorescence of the docking-strand fluorophores (Cy5, Cy3 or Cy3.5) with the use of a multi-spectral epi-fluorescence exciter (CoolLED). For PAINT or dSTORM imaging, either a 200 mW 561 nm (Cobalt Jive-200) laser or a 140 mW 642 nm (Omicron) laser were used. The laser excitation intensity was controlled with a computer driven neutral density filter wheel (Thorlabs) and accousto-optic modulators built into the laser modules. Single molecule images were acquired with a scientific-CMOS camera (Andor, UK) operated with integration times of either 100 ms (PAINT experiments) or 25 ms (dSTORM). The image data were acquired and analysed in real-time by a quad-core PC using the open source custom-written Python Microscopy Environment (PyME) software (freely available at http://python-microscopy.org/) Analysis of localisation data from the sCMOS camera was performed with algorithms that correct for non-uniform sCMOS pixel properties which are implemented in PyME as described recently (10).

Image analysis

Basic analysis and greyscale rendering: The frame data of DNA-PAINT single molecule events were analysed in real-time (during acquisition) using the PyME software developed by the consortium of co-authors (11). The analysis included the detection of single molecule events and least-squares fitting of a 2D Gaussian to localise their sub-pixel scale centroid as well as removal of the effect of sCMOS chip pixel non-uniformities by the use of camera-maps (10). The point data of the localised marker positions from a sub-series of 10,000 frames were selected for further analysis and events lasting more than one frame (tracked in consecutive frames to be within the localisation precision) were coalesced to minimise localisation errors – except for qPAINT analysis. For qPAINT analysis, as described in more detail below, un-coalesced single frame events were used. The event positions were then rendered into a 16-bit greyscale TIFF image with a pixel scaling of 1 nm/pixel using an algorithm based on Delaunay triangularisation (12). In these images, the pixel intensity was linearly proportional to the local density of localised markers, i.e. similar in its information content to a typical greyscale fluorescence micrograph albeit at higher spatial resolution.

Analysis of punctate nanoscale densities: The punctate RyR labelling densities in the rendered images were detected using a custom-written analysis algorithm implemented in PyME. This involved a smoothing of the images (kernel size of 3.0 pixels) and a multi-threshold detection of labelled regions. In a second round, an automatically-defined region of interest (ROI) capturing each of the punctate densities was least-squares fitted with an adaptive two-dimensional (2D) Gaussian model to determine the sub-pixel coordinates of the 2D centroid of each punctum. These centroids were used to count the number of observable RyRs within each cluster and to calculate the neighbour distances (e.g. Fig 3) through a Delaunay triangulation implemented with custom-written programs in the

IDL or Python programming language similar to previously described analyses (6). The centroids from RyR images were also used to construct Euclidean distance maps which were the basis for the distance-based density analysis of JPH2 labelling.

Area-based analysis of DNA-PAINT images: The greyscale rendered DNA-PAINT images were also subjected to threshold-based analysis of RyR cluster areas similar to previous dSTORM studies (5, 6). Using custom-written programs implemented in IDL or Python, a global threshold which encapsulated 80% of the total labelling fraction above background was adopted to generate a mask of the RyR labelled area. By filling in all dark 'holes' in connected mask regions that were smaller than 5000 nm^2, we robustly obtained a 2D mask of the overall RyR cluster area. These 2D masks were used for computing the 2D area of RyR clusters and for performing a co-localisation analysis on the overlap between JPH2 and RyR in two-colour DNA-PAINT data based on an algorithm detailed previously in the supporting material of (1). Briefly, this included the computation of two Euclidean distance maps, one containing the Euclidean distances in the regions outside of the RyR cluster (in relation to the cluster edge) and the other in the regions within the RyR cluster mask (also in relation to the cluster edge). Histograms of the integrated labelling of the second channel (e.g. JPH2) show labelling that is localised within the cluster ('negative' distances, shaded) and that outside of the cluster (positive distances, see e.g. supplementary Fig S5-B).

Quantitative analysis of DNA-PAINT data by qPAINT

qPAINT analysis of DNA-PAINT data is based on the idea that the temporal statistics of imager binding contains information about the number of binding sites (docking strands) given that the transient binding between individual imaging and docking strands follows first order binding kinetics. Specifically, the measured dark time τ_D is related to the number of binding sites N_B via

$$N_B = \frac{1}{k_{on} c_i \tau_D}$$

where k_{on} is the on-rate of imager to docking strand binding and c_i is the imager concentration. When using markers that contain α docking strands per marker and β markers bind on average to a single receptor, the number of receptors N_R is also proportional to τ_D^{-1}

$$N_R = \frac{N_B}{\alpha\beta} = \frac{1}{\alpha\beta k_{on} c_i} \frac{1}{\tau_D} = c' q_i$$

where we have lumped all constants into the constant c' and termed the inverse of the dark time the "qPAINT index" $q_i = \tau_D^{-1}$. N_R can be obtained from the uncalibrated qPAINT index q_i if the calibration value q_{i1} can be measured, where q_{i1} is the qPAINT index value for a single receptor. Inspection of the formulae then yields

$$N_R = \frac{q_i}{q_{i1}}$$

We pursued the calibration strategy as derived above to obtain RyR number estimates. The *in-situ* calibration was performed for each DNA-PAINT series since it is difficult to control the imager concentration c_i accurately between experiments. Care was taken to only analyse DNA-PAINT sequences that exhibit a constant event rate over the time window that was analysed for qPAINT quantification.

Measurement of dark times and qPAINT indices. Cluster event segmentation was performed based on cluster segmentation of rendered images. Cluster masks were obtained from rendered images as described in the section "area-based analysis of DNA-PAINT images" above. Events located within the cluster mask of a given cluster were grouped into the event group belonging to that cluster. The event group of a cluster and the associated event time stamps were used to reconstruct the sequence of dark times as contiguous frame times that did not contain an event. All dark times from a cluster were pooled to obtain a normalised cumulative histogram of dark times for the cluster. The resulting dark time histogram was fit with an exponential expression $1-\exp(-t/\tau_D)$ to estimate the dark time constant τ_D. The inverse of the dark time τ_D^{-1} was calculated for each cluster and stored as the qPAINT index q_i of the cluster.

Calibration of qPAINT data. To obtain estimates of RyR numbers N_R for each cluster, a calibration was performed for each DNA-PAINT series. This was achieved by calculating a histogram of cluster qPAINT indices for very small clusters as identified by a cluster area < 10,000 nm^2. Visually these corresponded to clusters containing one or few puncta. The calibration histogram generally exhibited a prominent peak at a small qPAINT index value q_{i1} and secondary peaks at multiples of this value. The calibration value was determined by a multi-

exponential histogram fit with peaks at multiples of the fitting parameter q_{i1}. qPAINT indices of all clusters were combined with the calibration value q_{i1} to obtain absolute RyR number estimates N_R as the ratio q_i / q_{i1}.

The custom code that implements the calibrated qPAINT analysis is available as part of a supplementary code package compatible with recent versions of PyME. The code package was developed by the Soeller laboratory and can be downloaded at http://bitbucket.org/christian_soeller/pyme-extra.

Simulation of synthetic data

We used an algorithm implemented in the PyME software to generate synthetic single molecule localisation data as performed previously (13). As a first step, model images of punctate RyR labelling densities placed in either (a) a gridded organisation at a fixed spacing (supplementary Fig S2A) or (b) randomly placed and at a variable spacing to the next nearest neighbour as described by a random sample from a normal distribution with a specified σ. The centroids of the puncta were then convolved with a 2D Gaussian model with a σ of 5 nm (i.e. with a full width at half maximum of 12 nm) to smooth the mask. The model was used as a starting template in the PyME software which generated single molecule events within the labelled regions to match the imaging parameters (length of acquisition, event rates, localisation error) observed in the dSTORM and DNA-PAINT experimental data for simulation of dSTORM and DNA-PAINT imaging, respectively. The parameters used for the synthetic data simulation are summarised in Table S2. The generated point data were rendered into 2D greyscale data and subjected to the centroid computation algorithm identical to the experimental analysis protocol described above to closely mimic the processing of the experimental data.

Supplementary References

1. Jayasinghe ID, Cannell MB, & Soeller C (2009) Organization of ryanodine receptors, transverse tubules, and sodium-calcium exchanger in rat myocytes. *Biophysical journal* 97(10):2664-2673.
2. Jungmann R*, et al.* (2014) Multiplexed 3D cellular super-resolution imaging with DNA-PAINT and Exchange-PAINT. *Nature methods* 11(3):313-318.
3. Huang F*, et al.* (2016) Ultra-High Resolution 3D Imaging of Whole Cells. *Cell* 166(4):1028-1040.
4. Soeller C, Crossman D, Gilbert R, & Cannell MB (2007) Analysis of ryanodine receptor clusters in rat and human cardiac myocytes. *Proceedings of the National Academy of Sciences of the United States of America* 104(38):14958-14963.
5. Baddeley D*, et al.* (2009) Optical single-channel resolution imaging of the ryanodine receptor distribution in rat cardiac myocytes. *Proceedings of the National Academy of Sciences of the United States of America* 106(52):22275-22280.
6. Hou Y, Jayasinghe I, Crossman DJ, Baddeley D, & Soeller C (2015) Nanoscale analysis of ryanodine receptor clusters in dyadic couplings of rat cardiac myocytes. *Journal of molecular and cellular cardiology* 80:45-55.
7. Van Oort RJ*, et al.* (2011) Disrupted junctional membrane complexes and hyperactive ryanodine receptors after acute junctophilin knockdown in mice. *Circulation* 123(9):979-988.
8. Tokunaga M, Imamoto N, & Sakata-Sogawa K (2008) Highly inclined thin illumination enables clear single-molecule imaging in cells. *Nature methods* 5(2):159-161.
9. McGorty R, Kamiyama D, & Huang B (2013) Active microscope stabilization in three dimensions using image correlation. *Optical Nanoscopy* 2(1):1-7.
10. Lin R, Clowsley AH, Jayasinghe ID, Baddeley D, & Soeller C (2017) Algorithmic corrections for localization microscopy with sCMOS cameras - characterisation of a computationally efficient localization approach. *Optics Express* 25(10):11701.
11. Baddeley D*, et al.* (2011) 4D super-resolution microscopy with conventional fluorophores and single wavelength excitation in optically thick cells and tissues. *PloS one* 6(5):e20645.
12. Baddeley D, Cannell MB, & Soeller C (2010) Visualization of localization microscopy data. *Microsc Microanal* 16(1):64-72.
13. Hou Y*, et al.* (2014) Super-resolution fluorescence imaging to study cardiac biophysics: alpha-actinin distribution and Z-disk topologies in optically thick cardiac tissue slices. *Prog Biophys Mol Biol* 115(2-3):328-339.
